# The screening for marine fungal strains with high potential in alkaloids production by *in situ* colony assay and LC-MS/MS based secondary metabolic profiling

**DOI:** 10.3389/fmicb.2023.1144328

**Published:** 2023-05-03

**Authors:** Tiantian Lu, Yayue Liu, Longjian Zhou, Qingnan Liao, Yingying Nie, Xingyuan Wang, Xiaoling Lei, Pengzhi Hong, Yan Feng, Xueqiong Hu, Yi Zhang

**Affiliations:** ^1^Guangdong Provincial Key Laboratory of Aquatic Product Processing and Safety, Guangdong Provincial Engineering Technology Research Center of Seafood, Shenzhen Institute of Guangdong Ocean University, College of Food Science and Technology, Guangdong Ocean University, Zhanjiang, China; ^2^Southern Marine Science and Engineering Guangdong Laboratory (Zhanjiang), Zhanjiang, China; ^3^Provincial Ministry Collaborative Innovation Center for Key Technologies of Marine Food Finishing and Deep Processing, Dalian Polytechnic University, Dalian, China

**Keywords:** alkaloids, *in situ* colony screening, LC-MS/MS, *Penicillium mallochii*, azaphilone, anti-neuroinflammatory

## Abstract

**Background:**

Alkaloids are the second primary class of secondary metabolites (SMs) from marine organisms, most of which have antioxidant, antitumor, antibacterial, anti-inflammatory, and other activities. However, the SMs obtained by traditional isolation strategies have drawbacks such as highly reduplication and weak bioactivity. Therefore, it is significantly important to establish an efficient strategy for screening strains and mining novel compounds.

**Methods:**

In this study, we utilized *in situ* colony assay combined with liquid chromatography-tandem mass spectrometry (LC-MS/MS) to identify the strain with high potential in alkaloids production. The strain was identified by genetic marker genes and morphological analysis. The secondary metabolites from the strain were isolated by the combine use of vacuum liquid chromatography (VLC), ODS column chromatography, and Sephadex LH-20. Their structures were elucidated by 1D/2D NMR, HR-ESI-MS, and other spectroscopic technologies. Finally, these compounds bioactivity were assay, including anti-inflammatory and anti-β aggregation.

**Results:**

Eighteen marine fungi were preliminarily screened for alkaloids production by *in situ* colony assay using Dragendorff reagent as dye, and nine of them turned orange, which indicated abundant alkaloids. By thin-layer chromatography (TLC), LC-MS/MS, and multiple approaches assisted Feature-Based Molecular Networking (FBMN) analysis of fermentation extracts, a strain ACD-5 (*Penicillium mallochii* with GenBank accession number OM368350) from sea cucumber gut was selected for its diverse alkaloids profiles especially azaphilones. In bioassays, the crude extracts of ACD-5 in Czapek–dox broth and brown rice medium showed moderate antioxidant, acetylcholinesterase inhibitory, anti-neuroinflammatory, and anti-β aggregation activities. Three chlorinated azaphilone alkaloids, **compounds 1–3** (sclerotioramine, isochromophilone VI, and isochromophilone IX, respectively), were isolated from the fermentation products of ACD-5 in brown rice medium guided by bioactivities and mass spectrometry analysis. **Compound 1** had shown remarkable anti-neuroinflammatory activity in liposaccharide induced BV-2 cells.

**Conclusion:**

In summary, *in situ* colony screening together with LC-MS/MS, multi-approach assisted FBMN can act as an efficient screening method for strains with potential in alkaloids production.

## Graphical Abstract

A comprehensive strategy “multi-approach assisted FBMN” for secondary metabolomic annotation combining FBMN, MSDIAL, MSFINDER, CFM-ID 3.0, and biological sources retrievement. 
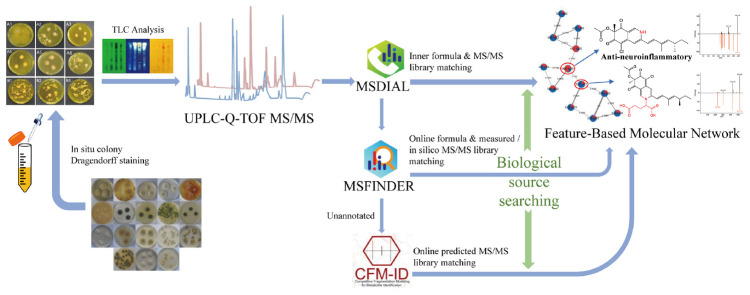

## 1. Introduction

With the depletion of terrestrial resources and the lack of inspiration in synthesizing effective novel drugs, marine-originated drugs attract more researchers’ attention based on their novel structures and biological activities (Haque et al., 2022). Particularly, discovering new marine natural products (MNPs) from fungi have outpaced discoveries from all the other marine phyla in the last few years (Carroll et al., 2021). Sourced from diverse habitats, fungi are “talented” organisms with the capacity to produce compounds with unique physiological activities. Fungal MNPs mainly include polyketones, peptides, alkaloids, terpenoids, etc. Among these classes, alkaloids are one of the most diverse groups (Yin et al., 2023). The current research on marine fungal alkaloids is mainly focused on the discovery of novel structures and their versatile biological activities. Despite the remarkable progress in marine alkaloids research, the emergence of new bioactive molecules still cannot fulfill the demand for drug discovery using traditional methods. New mining techniques for alkaloids from marine fungi will likely lead to new space for marine alkaloids chemical diversity.

Qualitative screening methods directly performed on microbial colonies are always favored because they can intuitionally reflect the potential of microorganisms in producing specific substances. Quite a few methods have been reported for such *in situ* studies using plate assays. Sawant reported a method using Gram’s iodine assay solution on fucoidan plates to observe the transparent circles to screen the alginate degrading enzymes-producing strains (Sawant et al., 2015). Except for the detection of alginate lyase, *in situ* colony assay was also reported that could be used to screen the fungi with antibacterial activity (Guan, 2019). However, *in situ* colony screening of alkaloid-producing strains has not been reported. The traditional screening of alkaloid-producing strains is performed by fermentation of all the tested strains, metabolites extraction, preliminary thin-layer chromatographic (TLC) colorization analysis using dyes like Dragendorff reagent, and in-depth diversity analysis using high performance liquid chromatography (HPLC), liquid chromatography-mass spectrometry (LC-MS), or direct compound isolation and structural elucidation (Yin and Sun, 2011; Han et al., 2015; Munakata et al., 2022). This pipeline is laborious, time-consuming, long-period, and unpredictable to the structural information. Inspired by the other plate assays, we attempted to develop an *in situ* colony screening method performed directly on agar plates for marine fungal alkaloid producers. This strategy could quickly identify the potential strains, slash workload from the begin and elevate the efficiency compared with the traditional screening pipeline.

To reliably recognize the alkaloids (or nitrogen-containing molecules) in crude extracts, LC-MS/MS instruments with high mass resolution like ultra-high-performance liquid chromatography quadrupole time-of-flight mass spectrometry (UPLC-QTOF-MS) are usually adopted due to its sensitivity and the ability to provide molecular formula and fragmentation information. For example, Li set up a mining strategy for rapid discovery and identification of lycorine-type alkaloids (Li et al., 2019). However, such a strategy relies on an in-depth understanding for the specific type of alkaloids and is not suitable for microbial strains with unclear background of metabolites. As a rising tool, Global Natural Products Social Molecular Networking (GNPS) allows the visualization of all the molecular ions detected in LC-MS/MS, and reveals their structural relationships based on MS/MS similarity. Besides, it also can present possible annotations by comparison with the MS/MS spectral records in metabolite databases even the researchers have little information on the strain metabolite background and fragmentation rules (Wang et al., 2016, 2022; Nie et al., 2020). Guided by GNPS, many new alkaloids like lipopeptides microcolins E–M and pyrrole-derived alkaloids phallusialides A–E have been reported to be isolated from different marine microorganisms (Yu et al., 2019; Zhang et al., 2019).

On the basis of classic GNPS, an updated version called Feature-Based Molecular Networking (FBMN) has been developed to enhance its ability to recognize different adduct ion forms for the same compound and discriminate isomers with different retention times (Nothias et al., 2020). Besides, the formula and MS^2^ information obtained from LC-MS/MS for FBMN construction can also be used to annotate the possible metabolites by automated MSDIAL-MSFINDER searching in multi-databases such as HDMB, YMDB, COCONUT, KNApSAcK, Natural Product Atlas, Drug Bank, PubChem, etc., through formula matching and comparison between measured MS/MS spectra and reference MS/MS (or even *in silico* calculated MS/MS spectra) (Lai et al., 2018; Tsugawa et al., 2019). Some new tools like CFM-ID version 3.0 (or 4.0) can assist in manually identifying structures via similar ways but with stronger ability to compare measured/predicted MS/MS spectra under multiple collision energies and annotate ion fragments (Djoumbou-Feunang et al., 2019; Wang et al., 2021). However, the above-mentioned tools also have their shortcomings if applied individually. For example, FBMN is not easy to compare with compounds without experimental MS/MS, MSDIAL is not convenient to match multiple databases, MSFINDER annotation does not consider the internal relationships between the metabolites in the sample, and all these tools do not give direct biological source annotations to the candidates. Thus, it is common for compounds to be annotated inappropriately.

In the present study, to screen fungal alkaloid producers intuitively and efficiently, the authors established an *in situ* colony screening method using Dragendorff reagent as dye in combined use with LC-MS/MS and FBMN assisted by multiple approaches (MSDIAL-MSFINDER, biological source annotation, and CFM-ID). This pipeline can quickly identify the potential alkaloid producer strains at the early step, greatly reduce the workload of fermentation-extraction, and display the diversity of alkaloid metabolite for background unclear strains. In this paper, we reported the *in situ* colony dyeing screening on marine fungal strains, diversity displaying of alkaloids by TLC colorization, LC-MS/MS profile, and multi-approach assisted FBMN networking, and the biological identification of the most fertile alkaloid producer strain ACD-5. The isolation and elucidation of three representative metabolites chlorinated azaphilones from this strain, as a proof for the effectiveness of this method, was also reported with related bioactivity study.

## 2. Materials and methods

### 2.1. Materials

Eighteen fungal strains from different hosts (seaweeds, coral, and sea cucumber) were previously isolated by our team and used for this study (Table 1). Four culture mediums were used for fermentation including Czapek–Dox Broth (CDB), Potato Sucrose Broth (PSB), Malt Extract Broth (MEB), and Brown Rice Medium (BRM). The mediums used for morphological identification of the strain ACD-5 included Potato Dextrose Agar (PDA), Blakeslee’s Malt Extract Agar (MEA), Czapek Yeast Agar (CYA), and Yeast Extract Sucrose Agar (YES). Czapek–Dox Agar (CDA) was used for the *in situ* colony screening experiment. 20.0 g/L sea salts were added to all the above culture media. The details of all the cultural mediums involved in this study were listed in Supplementary Table 1.

**TABLE 1:** Strains information used in this study.

Strain	Host	Collection place
S5-2-1	*Codium fragile*	Yangmeikeng, Shenzhen
S5-2-2	*Codium fragile*	Yangmeikeng, Shenzhen
S5-3	*Codium fragile*	Yangmeikeng, Shenzhen
S5-5-1	*Codium fragile*	Yangmeikeng, Shenzhen
S5-5-2	*Codium fragile*	Yangmeikeng, Shenzhen
S6-3 Y	*Sargassum henslowianum*	Yangmeikeng, Shenzhen
S6-3 Y2	*Sargassum henslowianum*	Yangmeikeng, Shenzhen
S7-1	*Grateloupia turuturu*	Yangmeikeng, Shenzhen
S7-1-1	*Grateloupia turuturu*	Yangmeikeng, Shenzhen
S7-3	*Grateloupia turuturu*	Yangmeikeng, Shenzhen
C23-1	*Pavona lamarck*	Coral reserve, Zhanjiang
C23-3	*Pavona lamarck*	Coral reserve, Zhanjiang
CD-1	*Holothuria scabra*	Meizhen sea cucumber breeding factory, Zhanjiang
NY-8	*Holothuria scabra*	Meizhen sea cucumber breeding factory, Zhanjiang
ACD-2	*Holothuria scabra*	Meizhen sea cucumber breeding factory, Zhanjiang
ACD-5	*Holothuria scabra*	Meizhen sea cucumber breeding factory, Zhanjiang
ACD-6	*Holothuria scabra*	Meizhen sea cucumber breeding factory, Zhanjiang
ACD-12	*Holothuria scabra*	Meizhen sea cucumber breeding factory, Zhanjiang

The 1D/2D Nuclear Magnetic Resonance (NMR) spectra were recorded on a Bruker Advance 700 MHz spectrometer (^1^H/^13^C, 700/175 MHz). LC-MS/MS data were recorded on a Waters Acquity UHPLC-DAD-Xevo G2-XS Q-Tof liquid chromatography-mass spectrometer. High resolution electron spray ionization mass spectrometry (HR-ESI-MS) data for pure compound were recorded on a Bruker Daltonics maXis impact mass spectrometer. Circular dichroism (CD) spectrum was recorded on a Chirascan spectropolarimeter (Applied Photophysics). Optical rotation was measured using an Anton Paar MCP–500 (Anton Paar, Graz, Austria). A 96-well microplate reader (Bio-Tek Epoch 2) was used for spectrophotometric measurements. A Di B60F microscope and a TESCAN MIRA 3 scanning electron microscope were used for fungal morphological observation. Solvent was evaporated through a EYELA rotary evaporator with a vacuum pump. Column chromatographies (CC) were performed on 200–300 mesh silica gel purchased from Qingdao Marine Chemical Factory and Sephadex LH-20 (Amersham Pharmacia). Precoated silica gel plates purchased from Qingdao Kai Bang Chemical Group Co (G60, F-254) were used for TLC analysis.

### 2.2. *In situ* colony assay

The activated fungal strains on slants were inoculated onto the CDA petri dishes with diameter of 9 cm at 28°C for 4 days. And then the strains were dyed with Dragendorff reagent to cover the surface of the strain colonies for 10 min. The formula and preparation of the Dragendorff reagent was [liquid A: bismuth nitrate 0.52 g, glacial acetic acid 6 mL, and pure water 24 mL. liquid B: potassium iodide 12 g and pure water 30 mL. After mixing liquid A and B, pure water 120 mL and glacial acetic acid 160 mL were added]. After the dyeing, the chromogenic agent was poured out and the color change of the colonies was observed. The colonies turned orange were selected as positive ones since Dragendorff reagent can form orange complex with alkaloids (Rubia and Gomez, 1977). Blank agar plates were used to monitor the sterile condition. All experiments were carried out in two repeats after a pilot study.

### 2.3. Preparation of strain fermentation crude extracts

The positive strains which had shown obvious yellow to orange colored colonies in the above *in situ* dyeing screening were picked out for further fermentation. They were inoculated into CDB, PSB, MEB, and BRM for a 3 weeks static fermentation, respectively. The fermentation was performed in 150 mL conical flasks filled with 30 mL of liquid or solid culture medium. The contaminated cultures would be replaced in time and a blank control was set to prevent the loss caused by fungal contamination. When the cultivation was finished, the fermentation broth was extracted with equal volume of ethyl acetate. Then, the broth extracts were concentrated under vacuum. Mycelium was extracted using 30 mL ethyl acetate: methanol (v/v = 1:1) assisted with ultrasonic for 30 min, and then concentrated. The broth and mycelium extracts for each culture were combined and evaporated to dryness. As for the solid cultures on BRM, 30 mL of methanol was used for 30 min ultrasonic assisted extraction, and the extraction was repeated for three times to collect filtrate which was concentrated to dryness. The crude extracts were dissolved with methanol to a concentration of 10 mg/mL and kept in a refrigerator at 4°C until use.

### 2.4. TLC analysis

The mobile phase for TLC was dichloromethane: methanol (v/v = 5:1) and the sample volume was 10 μL. The plates were observed under 254 and 365 nm UV lamps. Then, the plates were evenly sprayed with Dragendorff reagent and observed for the colorization of the spots. All experimental results were recorded by photography. Two known fungal products butyrolactone-I and epi-aszonalenin A were used as quality controls for non-alkaloids and alkaloids, respectively.

### 2.5. LC-MS/MS analysis

The selected extract samples were prepared as solutions with the concentration of 50 μg/mL using LC-MS pure methanol and pretreated with Agilent SPE column before LC-MS/MS analysis.

The LC-MS/MS analyses were run on a Waters Acquity UHPLC-DAD-Xevo G2-XS Q-Tof liquid chromatography-mass spectrometer. Waters ACQUITY UPLC BEH RP18 (2.1 × 50 mm, 1.7 μm) column was used for the analysis. The sample injection volume was 1.0 μL. The aqueous solution containing 0.1% formic acid was used as mobile phase A, and mobile phase B was acetonitrile. The gradient elution condition was as follows: 30% B (0–0.5 min), 30–80% B (0.5–4.0 min), 80% B (4.0–7.0 min), 80–30% B (7.0–7.2 min), 30% B (7.2–8.5 min) at a flow rate of 0.3 mL/min. The range of MS scan was set to *m/z* 50–2,000, electrospray ionization, and positive ion mode. The MS parameters were as follows: ion source temperature, 120°C; Capillary, 2 KV; Sampling Cone, 40 V; Source Offset, 80 V; Desolvation temperature, 450°C; Cone Gas, 50 L/h; Desolvation Gas, 700 L/h.

### 2.6. Multi-approach assisted FBMN analyses

After LC-MS/MS data acquisition, compounds feature table in the crude extracts was exported by upload converted raw data to MSDIAL Version 4.80 (The table information for features: Retention time; Precursor *m/z*; Adduct ion type; MS type; MS^1^ isotopic patterns; MS^2^ spectra). During the MSDIAL alignment running, an inner authentic standards database “MSMS_Public_EXP_Pos_VS17” from MSDIAL platform^1^ was also matched based on precursor mass and MS/MS similarity. The feature table was then automatically compared with different databases such as Drug Bank, PubChem, NANPDB, COCONUT, KNApASck, ChEBI, and UNPD by MSFINDER Version 3.52 to annotate compounds that each feature may represent. Furthermore, the alkaloids production in different culture mediums were analyzed and compared by FBMN using the above mentioned feature table and following the workflow on GNPS platform (Nothias et al., 2020). When the job was done, exported the result and visualized it using Cytoscape Version 3.70. All annotations from MSDIAL, MSFINDER, and FBMN were traced for their biological sources in important natural product databases (PubChem, COCONUT, LOTUS, NPA, DNP, CMNPD, NMRDATA, and others from which the candidates were matched) or original literature to verify their reasonability. If the candidates were from unreasonable organisms (e.g., structures typically from plants or animals but not from fungi) or the above three tools failed to annotate the metabolites, the MS^1^ and MS^2^ information will be submitted for structure identification onto CFM-ID 3.0 online platform^2^ (since its 4.0 version is yet not stable enough to visit) by comparison with the predicted MS^2^ spectra in 11 small molecule databases (HMDB, KEGG, MassBank, NIST, DrugBank, PhytoHub, FiehnLib, ContaminantDB, iTree, CASMI2016, and MetaboBASE). The resulting candidates were then traced for biological source again. Finally, the metabolites in the FBMN clusters were annotated on comprehensive account of the above approaches.

### 2.7. Bioassay

The crude extracts of the selected strain, or the metabolites that were subsequently isolated, were screened for the following activities.

#### 2.7.1. DPPH free radicals scavenging assay

The 1,1-diphenyl-2-picrylhydrazyl (DPPH) free radical scavenging potentials of crude extracts were evaluated in 96-well plates according to the manufacturer’s kit instruction. A total of 200 μL of the reaction mixture was composed of 100 μL DPPH and 100 μL sample with different concentrations in methanol (A_*sample*_). The reaction mixture was incubated for 30 min in darkness at room temperature and the absorbance was measured at 517 nm on the microplate reader. Meanwhile, A_*control*_ represented control group consisting of 100 μL samples and 100 μL methanol, A_*blank*_ represented blank group consisting of 100 μL DPPH and 100 μL methanol. Vitamin C was taken as a positive control. DPPH free radicals scavenging capacity was calculated by the formula:


$$\mathrm{Scavenging}\mathrm{capacity}\left(\%\right)=\left(1-\frac{A_{\mathrm{sample}}-A_{\mathrm{control}}}{A_{\mathrm{blank}}}\right)\times 100\%$$

Besides, an antioxidant TLC bioautography using DPPH as dye was also performed to display the diversity of antioxidant constituents in the extracts following our previously reported method (Nie et al., 2020; Wang et al., 2022).

#### 2.7.2. Acetylcholinesterase inhibition assay

The electric eel acetylcholinesterase (AChE) (Sigma-Aldrich, St. Louis, MO, USA) inhibitory activities were measured in 96-well plates by modified Ellman’s et al., 1961 methods. The concentration range of the positive control (donepezil) and the tested samples were set to be 0.01, 0.10, 0.25, 0.50, and 1.00 mg/mL (in DMSO). Likewise, an AChE inhibitory TLC bioautography was performed to show the diversity of AChE inhibitors following our previous reports (Nie et al., 2020; Wang et al., 2022).

#### 2.7.3. Cell viability assay

BV-2 cells were cultured in dulbecco’s modified eagle’s medium (DMEM) (GIBCO, Billings, MT, USA) supplemented with 1% of penicillin/streptomycin and 5% fetal bovine serum (FBS) (ZETA, Spring House, PA, USA) at 37°C in a moist 5% CO_2_ incubator. The cytotoxicity of the samples in different concentrations on cells were measured after 24 h incubation by MTT assay as previously described (Qian et al., 2012; Zhang et al., 2018).

#### 2.7.4. Nitric oxide (NO) production determination

To examine the inhibitory effect of the samples on the production of NO, BV-2 cells were seeded at density 2 × 10^4^ cells/well in 96-well plates. The cells were pre-treated with samples in different concentrations for 1 h and were subsequently activated by lipopolysaccharide (LPS) (Sigma-Aldrich, St. Louis, MO, USA) (1 μg/mL) for 24 h. The production of NO was measured using the Griess Reagent System according to the previous study (Ryu et al., 2010). The absorbance was measured at 540 nm by microplate reader.

#### 2.7.5. Gold nanoparticle-mediated anti-aggregation of amyloid-β

The preparation of gold nanoparticles (GNPs) was using Chah’s et al., 2005 method. Briefly, the particles were prepared by boiling 100 mL of aqueous 0.03% (w/w) hydrogen tetrachloroaurate (III) trihydrate (HAuCl_4_⋅3H_2_O) in a flask with constant vigorous stirring and then adding 3 mL of 3% (w/w) sodium citrate (Na_3_C_6_H_5_O_7_⋅2H_2_O). When the solution became almost opaque, heating was stopped and the solution was let cool to room temperature for 30 min. This GNPs solution was stored at 4°C for 24 h before use.

10 μL of Aβ solution consisting of 10 μM Aβ_1–40_ (Rhawn, China) and 10 μM Aβ_1–42_ (Yuanye, China) with a ratio of 9:1 (v/v) was first incubated with 10 μL of samples (20, 100, and 500 μg/mL) for 10 min. Then, this 20 μL solution was mixed with 90 μL of GNPs together with 10 μL of 5 mM Cu^2+^ and 10 μL of 30 mM HCl. After 5 min’s incubation, the absorbances were measured at 520 nm (as an intrinsic GNPs peak) and 650 nm (as an indicator peak of large fibrillar aggregation), respectively using a microplate reader (Kim et al., 2017). For sample testing, the final concentration of DMSO (co-solvent) was 5% in the testing system.

### 2.8. Strain identification by genetic marker genes

The genomic DNA was extracted from the strain grown on PDA using the Genomic DNA Extraction Kit (Solarbio, Beijing, China) following the manufacturer’s protocol. The internal transcribed spacer (ITS) was amplified and sequenced with primers ITS1 (5′-TCCGTAGGTGAACCTGCGG–3′) and ITS4 (5′-TCCTCCGCTTATTGATATGC–3′), while *BenA* (β-tubulin gene) with primers Bt2a (5′- GGTAACCAAATCGGTGCTGCTTTC –3′) and Bt2b (5′- ACCCTCAGTGTAGTGACCCTTGGC -3′). The PCR reactions were performed in 50 μL reaction mixtures containing 5 μL genomic DNA, 25 μL 2 × Phanta Max Buffer, 1 μL dNTP Mix (10 mM each), 1 μL Phanta Max Super-Fidelity DNA Polymerase, 2 μL each of the upstream and downstream primers and ddH_2_O using reagents from Vazyme (China) and primers from Sangon Biotech (China). The PCR parameters for ITS and *BenA* were pre-denaturation at 95°C for 3 min; denaturation at 95°C for 15 s, annealing at 56°C for 15 s, extension at 72°C for 90 s, 30 cycles, and complete extension at 72°C for 5 min. The PCR products were visualized by gel electrophoresis in a 1% agarose gel containing Ultra GelRed. The PCR amplification products were purified by gel cutting and ligated with T4 DNA ligase to pGM-T vector (Tiangen, China) overnight at 16°C. The pGM-T vector containing the target fragment was heat-stripped into sensitive *E. coli* TOP10 and incubated at 37°C for 45 min. The bacterial solution was spread onto LB agar plates containing ampicillin supplemented with X-gal and IPTG and incubated for 12-16 h at 37°C. The white clones were picked to identify whether the recombination was successful by direct PCR, and the positive PCR products were sequenced by Beijing Liuhe Huada Gene Technology Co., Ltd. (Beijing, China).

The sequencing results were compared with other known strains on the NCBI^3^ for gene sequence alignment to determine the strain genus. A phylogenetic tree was constructed by the neighbor-joining method using MEGA Version 11.0. The confidence value of each clade was assessed using bootstrap analyses based on 1,000 replications. The sequence was finally deposited in the GenBank database.

### 2.9. Morphological analysis

The strain ACD-5 was cultured on MEA, CYA, CDA, YES, and PDA agar plates, respectively, at 25°C for 7 days, with two parallels for each plate after a pilot study. Blank agar plates were used to monitor the sterile condition. The colony morphology of the strains was observed and recorded by camera. For microscopic observation, the strain was additionally inoculated on the PDA plates inserted with sterile coverslips at 45° obliquely near the inoculation spots at 25°C for 7 days. The coverslips with mycelium growth were pulled out after 4 days, fixed in 2.5% glutaraldehyde at 4°C for 2 h, rinsed in PBS for 15 min, dehydrated in gradients of 30, 50, 70, 90, and 100% ethanol for 15 min each, and dried naturally. The sample slides were placed in patches, sprayed with gold at 1.5 kV and 10 mA for 400 s. Finally, the fungal morphological structures were observed under the scanning electron microscope, photographed, and recorded. Besides, the coverslips with fresh mycelia were directly observed under optical microscope.

### 2.10. Fungal fermentation and characterization of the main products

The fungus ACD-5 was cultured on the PDA plate at 28°C for 4 days as seed culture. Then, the mycelium was inoculated into sixty–five 1 L-Erlenmeyer flasks, each containing 120 g of brown rice and 120 mL 2% artificial seawater. The culture was incubated at room temperature for 30 days. After culture, the rice with mycelium was extracted with ultrasonic for 30 min in methanol for three times, and the filtrate was collected and concentrated to dryness. A total of 80 g crude extracts were obtained.

Then the crude extracts were subjected to silica gel vacuum liquid chromatography (VLC) column, eluting with a gradient of *n*-hexane: ethyl acetate: methanol (v/v/v, 1:0:0, 1:1:0, 0:1:0, 0:1:1, 0:0:1, and successively) to obtain 5 fractions (Fr.1–Fr.5). Fr.3 was further purified by a silica gel VLC column eluted with *n*-hexane: ethyl acetate: methanol (v/v/v, 15:1:0 to 1:5:0 gradually, 0:1:0, 0:1:1, 0:0:1, and successively) to give 9 sub-fractions (SFr.3-1–SFr.3-9). SFr.3-5 was subjected to VLC eluted with dichloromethane: methanol (v/v, 80:1, 40:1) to give 9 subfractions (SFr.3-5-1–SFr.3-5-9). SFr.3-5-4 was recrystallized and repurified with ODS column chromatography eluted with 80% methanol/H_2_O to yield **compound 1** (35 mg). SFr.3-6 was purified by Sephadex LH-20 (dichloromethane-methanol, v/v, 1:1) to give 4 sub-fractions (SFr.3-6-1–SFr.3-6-4). SFr.3-6-3 was separated using ODS column (methanol/H_2_O, v/v, 4:1) and Sephadex LH-20 (dichloromethane-methanol, v/v, 1:1) to yield **compound 2** (13.6 mg). SFr3-7 was purified by VLC eluted with dichloromethane: methanol (v/v, 40:1 to 5:1 gradually) to give 5 sub-fractions (SFr.3-7-1–SFr.3-7-5). SFr3-7-3 was separated sequentially using ODS column (methanol/H_2_O, v/v, 7:3), Sephadex LH-20 (dichloromethane-methanol, v/v, 1:1), and ODS column (methanol/H_2_O, v/v, 4:1) to yield **compound 3** (12 mg).

The planar structures of **compounds 1–3** were elucidated by comparison of ^1^H and/or ^13^C NMR spectra with the report data in literature, and supported by high resolution mass spectrometry. And the stereo-structures were defined by comparison of the specific optical rotation and CD spectrum with the literature data.

### 2.11. Statistical analysis

The data were expressed by the GraphPad Prism Version 8.0.1 as the means ± SD (*n* = 3). A value of *p* < 0.05 was considered statistically significant. Differences between groups were calculated by One-way analysis of variance (ANOVA). For DPPH scavenging and AChE inhibitory assays, ^****^p < 0.0001 compared with positive control of the same concentration. For NO production determination, ^**^p < 0.01, ^****^p < 0.0001 compared with the LPS group. For cell viability assay, ^****^p < 0.0001, compared with the control group.

## 3. Results

### 3.1. *In situ* colony assay

This study focused on establishing an efficient alkaloid screening pipeline, of which the first step was an *in situ* colony dyeing screening. The ingredients of culture medium Czapek–Dox Agar (CDA) were mainly inorganic salts such as sodium nitrate, dipotassium hydrogen phosphate, and magnesium sulfate. It was free of alkaloid-like substances that may interfere with the results. Besides, the colorless and transparent plate made of CDA was conductive to the observation of color change. Thus, CDA was selected for *in situ* screening of strain producing alkaloids.

**FIGURE 1:**
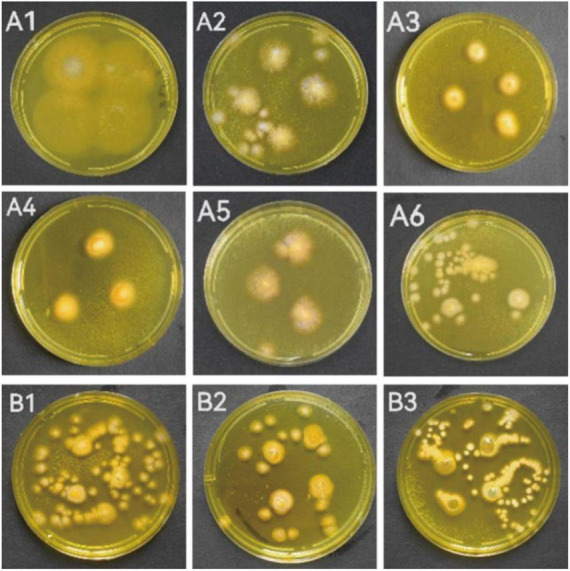
The strains showed alkaloid positive reaction in the *in situ* colony screening by Dragendorff reagent. **(A1–A6)** Strains from seaweeds; **(B1–B3)**: Strains from sea cucumber. (**A1**, S5-2-1; **A2**, S5-2-2; **A3**, S5-3; **A4**, S5-5-1; **A5**, S6-3 Y; **A6**, S7-3; **B1**, CD-1; **B2**, ACD-2; **B3**, ACD-5).

When the individual colony diameter reached 2–3 cm after 4 days, the colonies were dyed with Dragendorff reagent to discriminate the alkaloid producers. Among the 18 tested marine fungal strains, 9 strains (S5-2-1; S5-2-2; S5-3; S5-5-1; S6-3 Y; S7-3; CD-1; ACD-2 and ACD-5) showed obvious orange color on colonies surface compared with other strains, which indicated presumable alkaloids production (Figure 1 and Supplementary Figure 1). To perform this *in situ* colony screening, it is important to dye the colonies before they grow too old, otherwise some colonies would produce pigments that may interfere with the judgment.

### 3.2. TLC analysis

**FIGURE 2:**
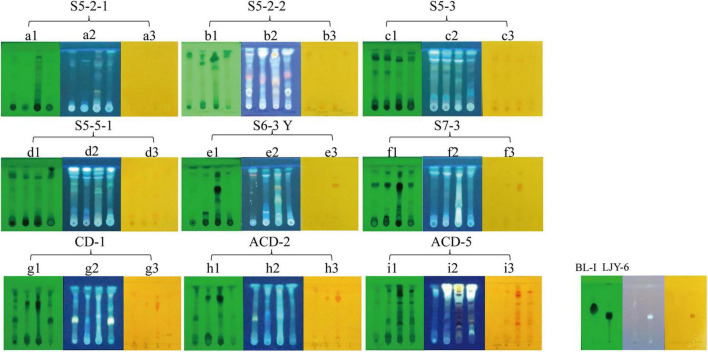
The TLC analysis of the positive strains. **(A1–I1)** UV pattern at 254 nm; **(A2–I2)** fluorescence pattern at 365 nm; **(A3–I3)** the results of Dragendorff reagent staining. The order of spotting on each plate from left to right is PSB, MEB, BRM, and CDB (representing different cultural medium used). BL-I was as a negative result and LJY-6 was as a positive result in TLC analysis.

To confirm the alkaloid producing ability of the positive strains selected by *in situ* colony screening, they were cultivated in four different media including CDB, PSB, MEB, and BRM, respectively. After 3 weeks of cultivation, their metabolites were extracted by organic solvents and analyzed on TLC plates stained with Dragendorff reagents to observe the presence of typical orange spots caused by alkaloids (Rubia and Gomez, 1977). As shown in Figure 2, two previously isolated compounds Butyrolactone I (BL-I, non-alkaloid) (Zhang et al., 2018) and epi-aszonalenin A (LJY-6, alkaloid) (Liu et al., 2022) from our laboratory were used as the controls and their respective negative and positive colorizations ensured the reliability of the TLC staining results. The crude extracts S5-3-PSB (means the fermentation extract of strain S5-3 in culture medium PSB, the next sample numbers follow the same rule), S5-3-BRM, S5-5-1-PSB, S5-5-1-BRM, S6-3Y-BRM, S7-3-BRM, CD-1-BRM, CD-1-MEB, ACD-2-BRM, ACD-2-MEB, ACD-5-BRM, and ACD-5-CDB presented significant alkaloid spots on TLC plates. Among them, ACD-5-BRM and ACD-5-CDB displayed the most abundant alkaloids. To have an insight to the alkaloid metabolites of the strain ACD-5 and their biological potential, this strain was selected for further secondary metabolic profile investigation by LC-MS/MS.

### 3.3. LC-MS/MS analysis of ACD-5

To investigate the metabolites diversity especially for alkaloids, the extract samples ACD-5-BRM and ACD-5-CDB were analyzed by LC-MS/MS and successive multi-approach assisted FBMN networking.

**FIGURE 3:**
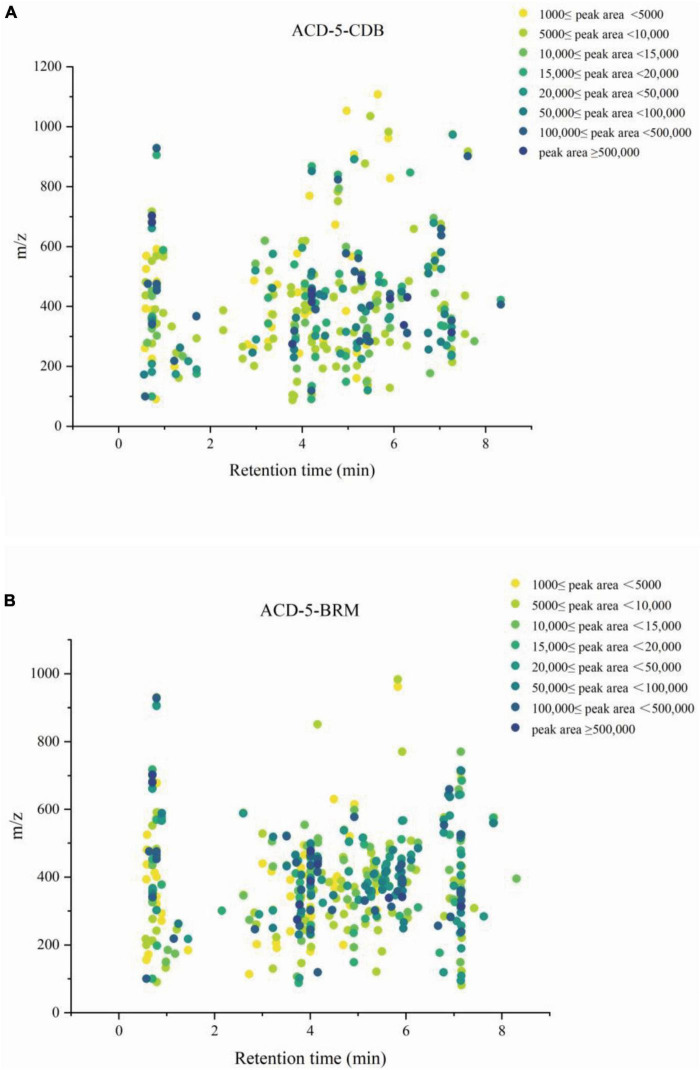
The metabolic profile of the features in extracts ACD-5-CDB **(A)** and ACD-5-BRM **(B)** showing their retention times, precursor ion *m/z* values, and intensities (exported from MSDIAL).

Firstly, MSDIAL-generated 2D peak spot heatmaps (Figure 3) showed the metabolic profiles of the two samples including the precursor *m/z* values, retention times, and peak areas of all the features and they were annotated primarily by matching the internally loaded database. The exported aligned feature information was then used for FBMN networking and MSFINDER multi-database searching, respectively. Their annotations were successively checked for biological source reasonability in several important natural product databases as described above in the (section of “2. Materials and methods”). The features unannotated by MSDIAL/FBMN/MSFINDER or the ones with obviously unreasonable biological resource were individually submitted to CFM-ID 3.0 for more opportunity of annotation by matching the plenty of *in silico* MS/MS spectra in databases. These CFM-ID annotations were then checked for biological source again. The final annotations to the FBMN network were synthetically determined on the basis of the above MSDIAL/FBMN/MSFINDER/CFM-ID—Biological Source Investigation pipeline, during which the structural relationships between features revealed by FBMN were also taken into crucial account.

**FIGURE 4:**
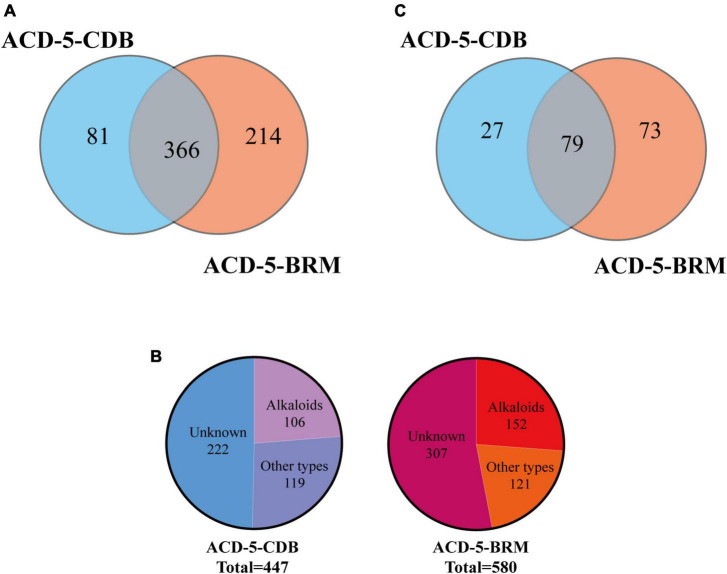
The statics of the numbers of the total features **(A)**, the annotated and unknown features **(B)**, and the alkaloids **(C)** detected in the two crude extracts of strain ACD-5.

**FIGURE 5:**
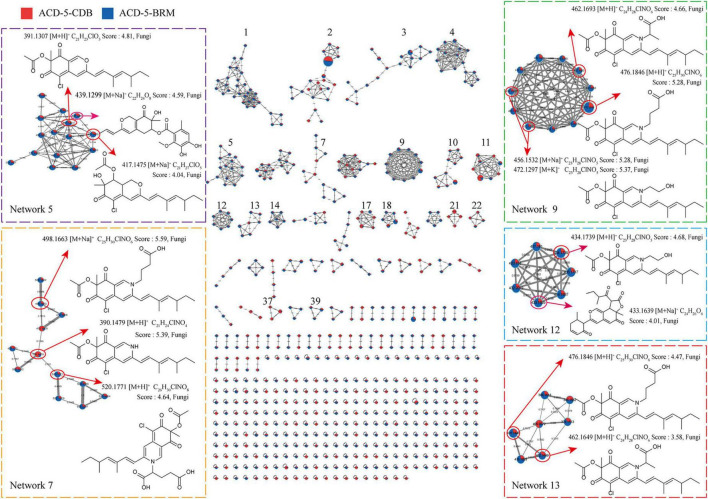
The FBMN molecular network based on positive ion MS/MS spectral similarity, showing a selection of amplified clusters. In the FBMN network, each node represents a feature marked with the mean *m/z* value of the parent ion, and the similarity of the secondary mass spectra between compounds was expressed by the cosine value, which was proportional to the similarity. The different colors of sections in the nodes represent different samples, i.e.,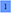 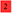 : respectively, ACD-5-BRM and ACD-5-CDB. The thickness of the connecting lines between nodes was positively correlated with the cosine value. The node size reflects the feature abundance (ion intensity).

Totally, 661 features were recognized by MSDIAL including 580 from ACD-5-BRM and 447 from ACD-5-CDB and 366 from both conditions (Figure 4A). In FBMN molecular networking, these 661 features from the two conditions formed 372 networks including 81 ones with more than 2 nodes (features) inside (Figure 5). By the above-mentioned multi-approach assisted FBMN annotation strategy, 227 metabolites including 106 alkaloids (or nitrogen-containing compounds) from ACD-5-CDB and 273 metabolites including 152 alkaloids from ACD-5-BRM were annotated, and 220 and 307 remained as unknown ones for the two conditions, respectively, (Figure 4B). Thus, this strain was confirmed as a fertile producer of alkaloids.

Intriguingly, a series of features (a total of 13 compounds) were annotated as plausible azaphilones most of which were nitrogenated ones as highlighted in Networks 5, 7, 9, 12, and 13 in the FBMN map (Figure 5 and Supplementary Table 1). They all showed high MSFINDER scores usually close to or even higher than 5 with fungal origins, and were mutually annotated by MSDIAL and FBMN (GNPS platform). As a sound proof, the ongoing isolation work has already yielded three **compounds 1–3** from ACD-5-BRM and their structures were elucidated by NMR, HR-ESI-MS, and CD (see detailed below in “Section 3.7. Isolation and identification of azaphilones”), which confirmed the result of LC-MS/MS based metabolic investigation. Judged from the parent ion intensity as indicated by the sector proportions in the nodes, the most of the azaphilones present higher yields in culture medium BRM than in CDB.

In addition to these azaphilones, this fungus can also produce a number of other types of alkaloids containing diverse nitrogenated moieties like diketopiperazines, tetrazoles, thiazoles, thiadiazolo, indoles, amines and acyl amines, purines, pyrimidines, isoquinolines, quinolines, quinazoline, pyridines, piperidines, amino acid residues, carbapenems, plumerans, pyrrolizine, diazepine, naphthyridine, sphingosine, cytisine, etc., and various molecular scaffolds (Supplementary Figure 2 and Supplementary Tables 1, 2). Similar to the distribution of azaphilones, this fungus also produced more alkaloids species in culture medium BRM than in CDB (Figure 4C).

Besides, this strain also produced other types of non-nitrogenated compounds, for example, high yields of long-chain lipids, steroids, phenols, and glycosides (Supplementary Tables 1, 2).

### 3.4. Bioactivities of the strain ACD-5’s fermentation extracts

As Alzheimer’s disease (AD) is among the most severe threats to human health and alkaloids usually cover related bioactivities, the fermentation extracts of strain ACD-5 were screened for related antioxidant, AChE inhibitory, anti-neuroinflammatory, and anti-Aβ aggregation activities.

**FIGURE 6:**
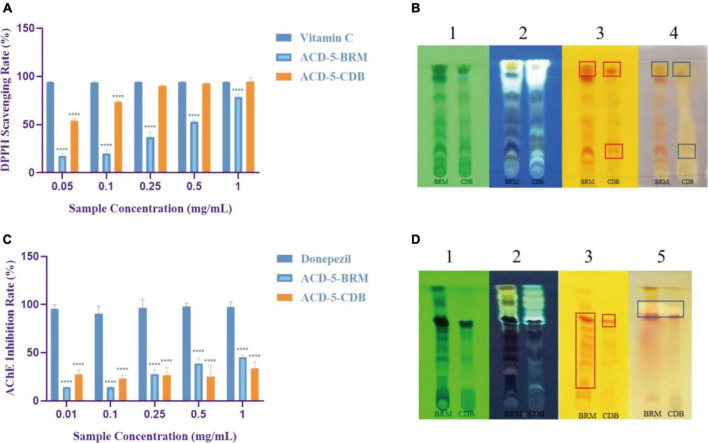
The DPPH scavenging and AChE inhibitory assays of the two crude extracts of strain ACD-5. **(A)** DPPH scavenging rate of ACD-5-BRM and ACD-5-CDB. Vitamin C was taken as a positive control. **(B)** The TLC-bioautography of the two extracts: (1) UV, (2) fluorescence, (3) Dragendorff reagent staining, (4) DPPH staining. **(C)** AChE inhibitory effect of ACD-5-BRM and ACD-5-CDB. The positive control was donepezil. **(D)** The TLC-bioautography of the two extracts: (1) UV, (2) fluorescence, (3) Dragendorff reagent staining, (5) AChE bioautography. The TLC developing system was dichloromethane: methanol = 5:1 (v/v). The results were expressed as the means ± SD (*n* = 3), ****: *p* < 0.0001 compared with positive control of the same concentration. A value of *p* < 0.05 was considered statistically significant.

The antioxidant effect of crude extracts was evaluated by their DPPH free radical scavenging ability. As shown in Figure 6A, the antioxidant effect of ACD-5-CDB was better than ACD-5-BRM. ACD-5-CDB scavenged DPPH free radicals in a dose dependent manner, and with 90% clearance rate in a concentration of 0.25 mg/mL. In the DPPH scavenging TLC bioautographic image, strong and rich active spots in ACD-5-CDB were observed to show clearing zone on the purple background stained by DPPH. By comparing the plates stained by DPPH and Dragendorff reagent, respectively, some of the antioxidant spots may be linked to alkaloids (Figure 6B).

Low levels of acetylcholine are one of the molecular characteristics of AD. Thus, AChE inhibitors have been traditionally valued as an important source of anti-AD drugs. As shown in Figure 6C, both the two extracts possess moderate AChE inhibitory activity with the highest inhibition rate of 45% for ACD-5-BRM and 33% for ACD-5-CDB. The AChE inhibitory TLC bioautography image also located the inhibitory compounds as white spots with Rf value of 0.82 in ACD-5-BRM and 0.79 in ACD-5-CDB, respectively, (Figure 6D).

**FIGURE 7:**
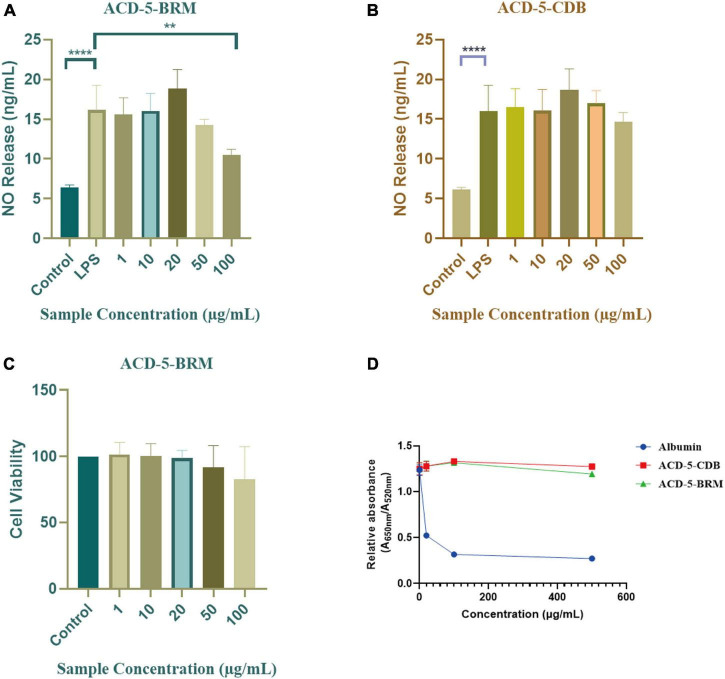
The anti-neuroinflammatory, cytotoxic, and anti-Aβ aggregation effects of the extracts ACD-5-BRM and ACD-5-CDB. **(A,B)** Inhibition to liposaccharide induced nitric oxide (NO) production of BV-2 cells of ACD-5-BRM **(A)** and ACD-5-CDB **(B)**, respectively. The results were expressed as the mean ± SD (*n* = 3). ^**^p < 0.01, ^****^p < 0.0001, compared with the LPS group. A value of *p* < 0.05 was considered statistically significant. **(C)** The effect of ACD-5-BRM on the viability of BV-2 cells. Since the quantity of ACD-5-CDB was insufficient, no cytotoxicity test was conducted. **(D)** The Anti-Aβ aggregation effect of ACD-5-CDB and ACD-5-BRM in gold nanoparticle-mediated anti-Aggregation test. Albumin was taken as a positive control.

The inhibitory to bacterial LPS induced NO production in microglial cells (BV-2) was tested for the two extracts. As shown in Figures 7A, B, when the BV-2 cells were pre-treated with increasing concentrations of the two crude extracts, the levels of NO were decreased significantly by ACD-5-BRM in 100 μg/mL. Furthermore, ACD-5-BRM did not exhibited obvious cytotoxicity to BV-2 cells in the concentration range of 0–100 μg/mL (Figure 7C). These results indicated that the crude extract ACD-5-BRM has potential anti-neuroinflammatory effect.

Oligomerization and fibrillation of Aβ are thought to be key steps in the pathogenesis of AD (Jucker and Walker, 2013). Aβ monomers and crude extracts with various concentrations were pre-incubated for 10 min and then GNPs were added as indicator to screen the anti-aggregation effects of ACD-5-CDB and ACD-5-BRM (Figure 7D). The anti-aggregation effect was negatively correlated to the ratio of absorbance at 650 nm to that at 520 nm (A_650nm_/A_520nm_) (Kim et al., 2017). The results indicated the anti-aggregation ability of ACD-5-BRM and ACD-5-CDB are much weaker than the positive control albumin.

### 3.5. Analysis of morphological characteristics

**FIGURE 8:**
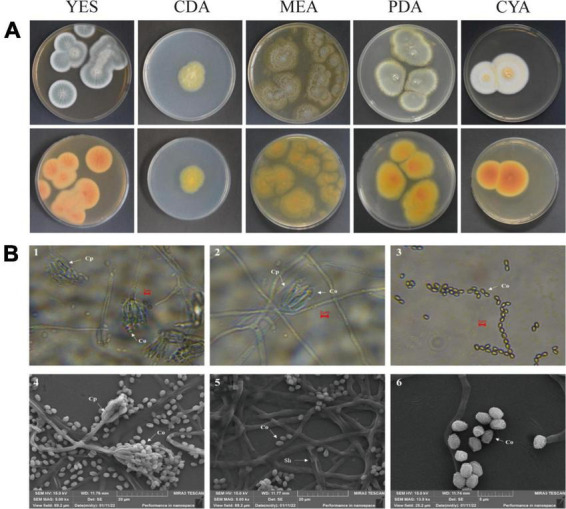
The colonial and microscopic morphology of ACD-5. **(A)** The 7-day old cultures of ACD-5 on CYA, CDA, MEA, YES, and PDA, showing variation in colony characters. The front view of colony on the left and the back view of colony on the right. **(B)** The microscopic morphology of strain ACD-5. 1–3 were morphologies observed under light microscope at 100 x magnification, scale bar = 2 μm; 4–6 were the morphologies observed under scanning electron microscope, the scale bar of 4 and 5 was 20 μm while the 6 was 5 μm. Co, Conidium; Cp, Conidiophore; Sh, Septa hypha.

After strain ACD-5 was cultured on MEA, CYA, CDA, YES, and PDA for 1 week at 25°C (Figure 8A), characteristics including colony morphology and growth rate were recorded (Table 2). The scanning electron microscope (SEM) and optical microscope analysis revealed that strain ACD-5 produced conidiophores monoverticillate, stipes septate, mostly unbranched, phialides 6–7.2 × 1.6–2.8 μm, ampulliform with a collula, conidia ellipsoidal, fine and rough, 2.3–3.8 × 1.6–2 μm, and mean L/W ratio 1.7: 1 (Figure 8B; Rivera and Seifert, 2011; Rivera et al., 2012).

**TABLE 2:** Cultural characteristics of strain ACD-5 after 1 week growth at 25°C.

Medium	Diameter (mm)	Colony characteristics	
		Front	Reverse
CYA	32–42	Dense and velutinous, sometimes with aerial mycelium in the center; conidia white to orange; produce clear orange exudate droplets; mycelium edges white.	Red-orange, light to pastel yellow at the margins.
CDA	27–30	Sparsely downy with aerial mycelium; conidia white to yellow; no exudate and soluble pigment.	Yellow.
MEA	25–38	Planar, with no aerial mycelium; conidia greyish green to dull green; no exudates or soluble pigments.	Yellow, deep yellow or orange, with lighter shades of these colors near the edges.
YES	27–38	Dense, velutinous; conidia white to greyish green; moderately sulcate, often wrinkled in the center; produce clear orange-yellow exudate droplets, yellowish exudate droplets.	Reddish-orange on the center.
PDA	40–42	Planar, with aerial mycelium in the center; conidia are greyish green to dull green with light yellow marginal spores; no exudate.	Yellow or red-orange.

### 3.6. Identification by phylogenetic analysis

**FIGURE 9:**
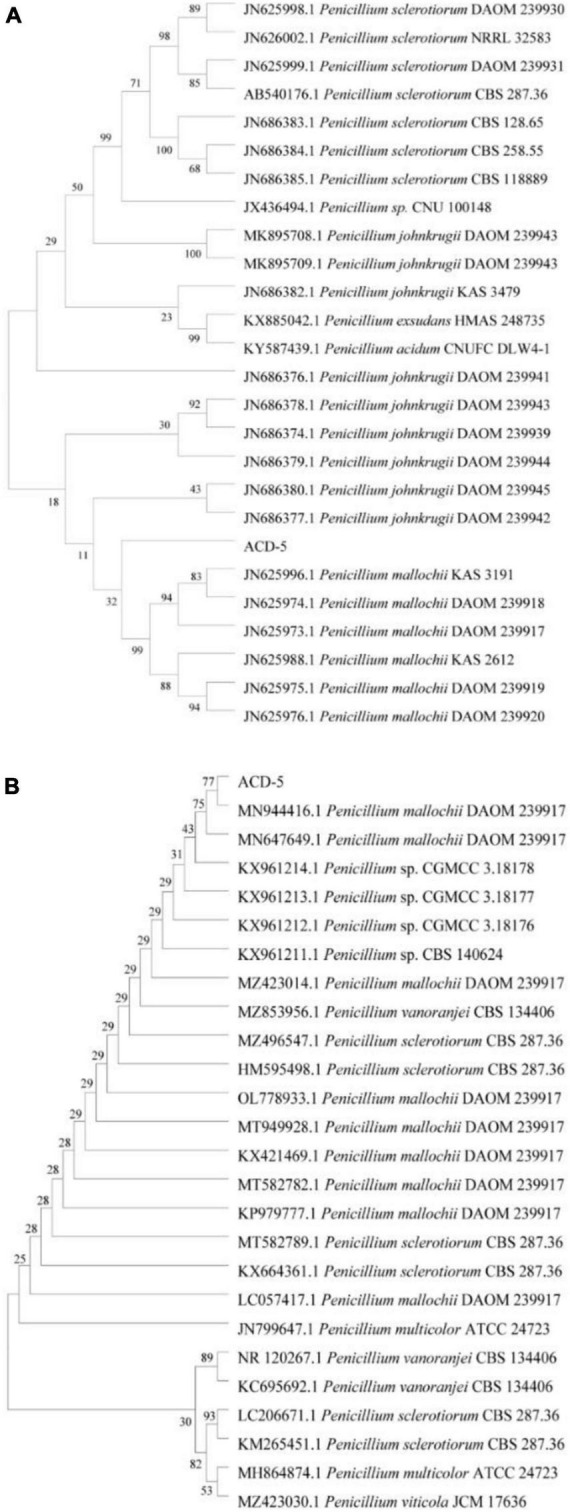
Neighbor-jointing trees based on β-tubulin **(A)** and ITS rDNA **(B)** of ACD-5 and adjacent areas of the dominant fungal groups (Bootstrap values were calculated using 1,000 replications).

The ITS and *BenA* sequences of strain ACD-5 were amplified and sequenced. After alignment, the ITS sequence showed the highest similarity (100%) to *Penicillium mallochii* DAOM 2,39,917 and the *BenA* sequence showed the highest similarity (100%) to *P. johnkrugii* DAOM 239943. It was supported by the phylogenetic trees constructed by the neighbor-joining approach (Figure 9). Combined with the morphological analysis, ACD-5 was identified as *P. mallochii*. Subsequently, the ITS sequence of ACD-5 had been submitted to the GenBank database of NCBI with the accession number OM368350. This strain is deposited in Guangdong Microbial Culture Collection Center with the deposit number of GDMCC62411.

### 3.7. Isolation, identification, and bioassay of azaphilones

In the above chemical analysis and bioassay, ACD-5-BRM showed generally richer alkaloids and better activities than those of ACD-5-CDB. Therefore, BRM was chosen for bulk fermentation of strain ACD-5, and a total of 80 g crude extracts were obtained after fermentation for metabolite isolation.

**FIGURE 10:**
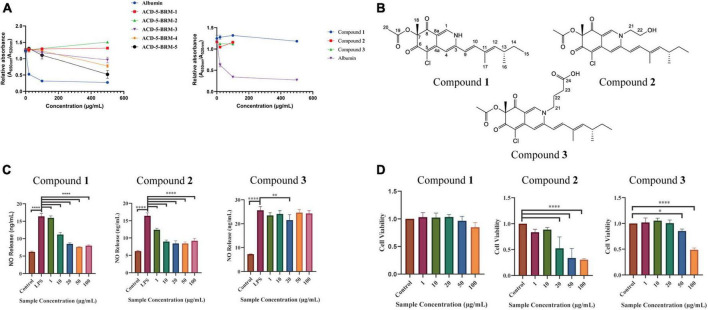
The anti-Aβ aggregation, anti-neuroinflammatory and cytotoxic effects of the primary fractions of ACD-5-BRM and the isolated **compounds 1–3**. **(A)** The anti-Aβ aggregation effects of the primary fractions of ACD-5-BRM and compounds. Since **compounds 2** and **3** were not soluble in methanol at high concentrations, anti-Aβ aggregation assay was not performed on 500 μg/mL. Albumin was taken as a positive control. **(B)** The structure of **compounds 1–3**. **(C)** The inhibitory effect of compounds on LPS induced nitric oxide (NO) production of BV-2 cells. **(D)** The effect of **compounds 1–3** on the viability of BV-2 cells. The results were expressed as the mean ± SD (*n* = 3). ^*^p < 0.05, ^**^p < 0.01, ^****^p < 0.0001, compared with the control group. A value of *p* < 0.05 was considered statistically significant.

The extract was subjected to a silica gel vacuum liquid chromatography (VLC), which was eluted with a gradient of *n*-hexane/ethyl acetate/methanol to obtain 5 fractions (Fr.1–Fr.5). In the anti-Aβ aggregation assay, Fr.3 to Fr.5 demonstrated increased anti-aggregation activities (Figure 10A). Analysis of them using LC-MS/MS revealed the presence of rich azaphilone alkaloids. Further separation of Fr.3 had led to the isolation of **compounds 1–3**.

**Compound 1** was obtained as a red powder. Its molecular formula was determined to be C_21_H_24_ClNO_4_ by the positive HR-ESI-MS with the quasimolecular ion peak at *m*/*z* 390.1471 [M + H]^+^ (calcd. 390.1467 for C_21_H_25_ClNO_4_^+^) and the typical 3:1 isotopic pattern for monochlorinated molecules. The ^1^H/^13^C NMR data were listed as follows. ^1^H NMR (DMSO-*d*_6_, 700 MHz): δ_*H*_ 7.96 (1 H, s, H-1), 6.95 (1 H, s, H-4), 6.47 (1 H, d, *J* = 16.3 Hz, H-9), 7.24 (1 H, d, *J* = 16.3 Hz, H-10), 5.71 (1 H, d, *J* = 9.7 Hz, H-12), 2.49 (1 H, m, H-13), 1.30∼1.41 (2 H, m, H-14), 0.84 (3 H, t, *J* = 7.4 Hz, H-15), 0.98 (3 H, d, *J* = 6.6 Hz, H-16), 1.83 (3 H, s, H-17), 1.40 (3 H, s, H-18), and 2.07 (3 H, s, H-20). ^13^C NMR (DMSO-*d*_6_, 175 MHz): δ_*C*_ 139.1 (C–1), 146.2 (C–3), 110.6 (C–4), 146.1 (C–4a), 100.3 (C–5), 183.0 (C–6), 85.3 (C–7), 193.5 (C–8), 113.5 (C–8a), 118.5 (C–9), 141.5 (C–10), 132.8 (C–11), 146.8 (C–12), 34.7 (C–13), 30.0 (C–14), 12.3 (C–15), 20.6 (C–16), 12.7 (C–17), 20.5 (C–18), 169.6 (C–19), and 20.6 (C–20). Its specific optical rotation was ${\left[\alpha \right]}_{D}^{25}$+408° (*c* 0.01, MeOH). CD (*c* 0.02, MeOH) λmax (Δε) 215 (−6.66), 246 (+4.87), 309 (−4.64) and 381 (+8.24) nm. By comparison of its ^1^H and ^13^C NMR data as well as CD spectrum with the reported data in literature (Jia et al., 2019), **compound 1** was elucidated to be sclerotioramine with the same stereochemistry (7*R*, 13*S*) (Figure 10B).

**Compound 2** was obtained as a red powder. The molecular formula of **compound 2** was determined to be C_23_H_28_ClNO_5_ by the HR-ESI-MS peak at *m*/*z* 433.17 [M + H]^+^, with the 3:1 chlorine isotope peaks. The ^1^H NMR data was listed as follows. ^1^H NMR (DMSO-*d*_6_, 700 MHz): δ_*H*_ 8.09 (1 H, s, H-1), 6.95 (1 H, s, H-4), 6.53 (1 H, d, *J* = 15.5 Hz, H-9), 7.10 (1 H, d, *J* = 15.5 Hz, H-10), 5.83 (1 H, d, *J* = 9.70 Hz, H-12), 2.47 (1 H, m, H-13), 1.30∼1.41 (1 H, m, H-14), 0.84 (3 H, t, *J* = 7.0 Hz, H-15), 0.98 (3 H, d, *J* = 6.7 Hz, H-16), 1.85 (3 H, s, H-17), 1.40 (3 H, s, H-18), 2.07 (3 H, s, H-20), 4.21 (2 H, m, H-21), and 3.66 (2 H, m, H-22). Its specific optical rotation was ${\left[\alpha \right]}_{D}^{25}$+590° (*c* 0.01, MeOH). CD (*c* 0.02, MeOH) λmax (Δε) 226 (+0.55), 244 (+4.04), 304 (-5.48) and 380 (+4.44) nm. By comparison of its ^1^H NMR data with **compound 1** and the reported data in literature (Jia et al., 2019), **compound 2** was elucidated to be isochromophilone VI with the same stereochemistry (7*R*, 13*S*) (Figure 10B).

**Compound 3** was obtained as a red powder. The peak *m/z* 475.18 [M + H]^+^ and the chlorine isotope peaks in HR-ESI-MS spectrum indicated the molecular formula was C_25_H_30_ClNO_6._ The ^1^H NMR data was listed as follows. ^1^H NMR (DMSO-*d*_6_, 700 MHz): δ_*H*_ 8.18 (1 H, s, H-1), 6.97 (1 H, s, H-4), 6.54 (1 H, d, *J* = 15.5 Hz, H-9), 7.17 (1 H, d, *J* = 15.5 Hz, H-10), 5.85 (1 H, d, *J* = 9.7 Hz, H-12), 2.49 (1 H, m, H-13), 1.31 ∼ 1.44 (1 H, m, H-14), 0.84 (3 H, t, *J* = 7.4 Hz, H-15), 0.98 (3 H, d, *J* = 6.6 Hz, H-16), 1.90 (3 H, s, H-17), 1.40 (3 H, s, H-18), 2.07 (3 H, s, H-20), 4.13 (2 H, m, H-21), 1.93 (2 H, m, H-22), and 2.32 (2 H, t, *J* = 6.7 Hz, H-23). Its specific optical rotation was ${\left[\alpha \right]}_{D}^{25}$ + 1240° (*c* 0.01, MeOH). CD (*c* 0.02, MeOH) λmax (Δε) 231 (+0.35), 244 (+2.89), 305 (-3.45), and 378 (+2.98) nm. By comparison of its ^1^H NMR data with the above two azaphilones and the reported data in literature (Jia et al., 2019), **Compound 3** was elucidated to be isochromophilone IX with the same stereochemistry (7*R*, 13*S*) (Figure 10B).

In the bioassays, **compound 1** displayed remarkable inhibition to LPS induced NO release in microglial BV-2 cells at the dose of 10–100 μg/mL without toxicity to this cell within the same concentration range (Figures 10C, D). **Compound 2** also exhibited inhibition to NO release without cytotoxicity at 1 and 10 μg/mL; however, it produced toxicity to the cell at higher concentrations. **Compound 3** only showed weak inhibition to NO release at 20 μg/mL. This suggested the potential of **compound 1** as anti-neuroinflammatory agent. Judged from their structures, the too long side chain on N-2 position seems to weaken their anti-neuroinflammatory activity or increase cytotoxicity. In the anti-Aβ aggregation activity assay, no significant effect comparable to the control albumin was observed for these three compounds. **Compounds 2** and **3** displayed relatively higher anti-aggregation effect than **compound 1** at 20 and 100 μg/mL. However, their low solubility in the test system, hampered their testing at higher concentration. Considering the anti-aggregation effect of Fr.3 to Fr.5, we speculate that more azaphilones with strong activity may be found later from these fractions.

## 4. Discussion

In this study, a *Penicillium mallochii* strain derived from sea cucumber with diverse alkaloid metabolites was screened out from marine fungal collection by *in situ* colony dyeing and multi-approach assisted FBMN and identified by genetic markers and morphology. Intriguingly, it is capable of producing rich azaphilones, most of which belong to alkaloids. This ability was confirmed by the isolation and elucidation of three representative **compounds 1** (sclerotioramine), **2** (isochromophilone VI), and **3** (isochromophilone IX). Its crude extracts, fractions, and/or isolated compounds exhibited antioxidant, AChE inhibitory, anti-Aβ aggregation, and anti-neuroinflammatory effects.

The traditional screening of alkaloid-producing strains is usually carried out via alkaloids detecting in culture extracts by chromogenic reactions in liquid or on TLC plate and by HPLC, LC-MS, or direct ESI-MS (Yin and Sun, 2011; Han et al., 2015; da Silva et al., 2021; Munakata et al., 2022). However, these methods usually require a term of fermentation before extraction of metabolites from all the tested strains in the first step. It is usually time-consuming and laborious, though sometimes the extraction can be skipped. While *in situ* colony assay in our study has proven to be a more convenient and effective method as it was performed by direct color development of the strain to determine whether it can produce alkaloids, which can help us shorten the range and find the target strains quickly. LC-MS/MS is well known for its wide analytical range, reliable qualitative analysis results and low detection limits. *In situ* colony assay combined with high-resolution LC-MS/MS enabled us to quickly find the alkaloid producing strains in the screening. Although some new technologies like DESI-MS (desorption electrospray ionization mass spectrometry) and DART-MS (direct analysis in real-time mass spectrometry) may be used for direct MS analysis on colonies (Tata et al., 2015; Gross, 2014), the minute contents of secondary metabolites (SMs) in colony may limit the completeness of information and much more accurate instruments are needed.

Mass spectrometry-based molecular networks are established on the similar MS/MS fragmentation for the structurally similar molecules. To discover potential SMs, molecular networking tools like GNPS can provide useful information to annotate possibly known natural products and find novel compounds in the dereplication of strains. The updated version of GNPS, i.e., FBMN can make use of data containing molecular formula and retention time information collected from high-resolution mass spectrometry and thus enhances the annotation function to discriminate isomers (Nothias et al., 2020). MSDIAL, MSFINDER, and CFM-ID are also practical tools for metabolites annotation. However, GNPS or FBMN usually provides very limited hits due to the capacity of the measured MS/MS spectral databases. While MSDIAL, MSFINDER, and CFM-ID merely annotate compounds separately without considering the internal relationship between metabolites. And a mutual shortcoming of these approaches is that the reasonable biological sources of compounds have not been taken into account during annotation.

In the present study, we developed a comprehensive strategy named “multi-approach assisted FBMN” to annotate the metabolites in the extracts of the strain ACD-5 (Graphical Abstract). This strategy could fully utilize the advantages of FBMN (networking view, multiple measured MS/MS spectral libraries), MSDIAL (inner set measured MS/MS library), MSFINDER (multiple measured and *in silico* MS/MS spectral libraries), and CFM-ID 3.0 (rich *in silico* MS/MS spectra with different collision energies predicted for natural product libraries). In addition, the biological sources of the hits were investigated by manual retrieving in quite a few important natural product libraries. The final annotations were made on the basis of comprehensive consideration of these results. With this strategy, diverse alkaloids together with other compounds were annotated for strain ACD-5. Particularly, a series of azaphilones were found in the extracts and their high scores and fungal origin in literatures demonstrated the effectiveness of this metabolic annotation strategy. However, it is reminded that the consideration of the biological source should be cautiously given priority since some natural products are evolutionarily conservative between different taxa and it is not excludable the residue of plant-derived ingredients in cultural medium.

Marine fungi are one of the important sources of MNPs due to their ability to produce abundant SMs and the potential for sustainable utilization. ACD-5 was isolated from the sea cucumber *Holothuria scabra* intestine and was identified as *P. mallochii* by gene sequencing and morphology. Little is known about this fungal species up to now. *P. mallochii* has been first isolated from *Rothschildia lebeau* and *Citheronia lobesis* larvae in Costa Rica and is monoverticillate with greenish globose to sub-globose conidia, smooth-walled conidiophores, and an orange-red extracellular pigment in agar culture (Rivera et al., 2012). There are fewer reports on its SMs and activities. The chlorinated azaphilone sclerotiorin is the first secondary metabolite reported from this species (Maccurin and Relly, 1940). Herein, we discover the great potential of *P. mallochii* strain ACD-5 in the production of alkaloids, including diverse azaphilones, by *in situ* colony screening and LC-MS/MS.

Azaphilones have been found with versatile bioactivities and structures. A previous report showed that azaphilones produced by *P. mallochii* exhibited an inhibitory effect on glioblastoma and the effective dose showed no lethal properties in healthy cells (Bouhri et al., 2020). Furthermore, sclerotiorin was reported to potently delay both seeded and non-seeded Aβ_42_ polymerization in cell-free assays. The azaphilones also have antioxidant and anti-inflammatory properties (Tang et al., 2019; Ayyolath et al., 2020) and have been described as modulators of tau aggregation *in vitro* (Paranjape et al., 2015). Thus, this type of compound has great potential as drug candidates for the treatment of AD (Wiglenda et al., 2020). In addition, azaphilones from different fungi were reported for their cytotoxic, antimicrobial, antitumoral, and antiviral (including inhibition to SARS-CoV-2, the pathogen of COVID-19) properties (Ayyolath et al., 2020; Pimenta et al., 2021; Youssef et al., 2021). Some azaphilones like sclerotiorin have no nitrogen atoms in structures while others like sclerotioramine contain nitrogen. Previous reports indicated that the pyranyl oxygens in their structures are readily converted to nitrogens with ammonia (Maccurin and Relly, 1940; Arai et al., 1995), which enhances their structural and bioactivity diversities.

In strain ACD-5, a total of thirteen azaphilones including nitrogenated and non-nitrogenated were annotated (Figure 5), most of them contain chlorin as well, and their structural scaffold or side-chain substitution are also quite different. Moreover, some other unannotated nodes were also observed to have high structural similarities with the annotated ones in the same networks as shown by their cosine values (higher than 0.8 and even close to 1.0). In anti-neuroinflammatory and anti-Aβ aggregation assays, the extracts and fractions of ACD-5 exhibited bioactivities, which need further investigation of their bioactive compounds especially azaphilones. Promisingly, **compound 1** (sclerotioramine) from fraction Fr.3 showed potent anti-neuroinflammatory activity without apparent toxicity to BV-2 cells. Therefore, ACD-5 is considered as a strain with great potential to be explored and we believe more azaphilones will be continuously contributed by this strain with in-depth isolation and application of metabolomic and genomic mining.

In summary, this study reported the discovery of a marine fungus *P. mallochii* ACD-5 from sea cucumber with high potential in alkaloids production through an integrated pipeline consisting of an efficient *in situ* colony assay, TLC chemical colorization and LC-MS/MS based metabolomics profiling. During this, a comprehensive metabolic annotation strategy named “multi-approach assisted FBMN” was established by combining MSDIAL, MSFINDER, FBMN, CFM-ID, and manual biological source annotation for metabolomics investigation. Using this strategy, diverse alkaloids especially azaphilones were annotated in the extracts of this strain. Its fermentation extracts, fractions and/or the isolated compounds displayed good antioxidant ability, inhibited Aβ aggregation and had anti-neuroinflammatory effect. The isolation and elucidation of the azaphilone alkaloids sclerotioramine, isochromophilone VI, and isochromophilone IX verified the effectiveness of our alkaloids “hunting” pipeline. As a fertile producer of potential alkaloids, the bioactive azaphilones and other metabolites are deserved to be mined from strain ACD-5 by further metabonomic, genomic, and pharmacological approaches.

## Data availability statement

The datasets presented in this study can be found in online repositories. The names of the repository/repositories and accession number(s) can be found in this article/Supplementary material.

## Author contributions

YZ and YL designed the research plan. TL mainly performed most of the experiments and prepared the draft. YN and XL participated in the experiments on sampling, isolation, culture of seaweeds, sea cucumbers, and corals. YF and LZ participated in the strain identification. QL was responsible for the assay of anti-inflammatory activity. XW was responsible for the assay of anti-aggregation of Aβ. PH and XH provided advice on the research. YZ supervised the work. YL, LZ, and YZ reviewed the manuscript. All authors contributed to the article and approved the submitted version.

## Footnotes

1
http://prime.psc.riken.jp/compms/msdial/main.html

2
https://cfmid.wishartlab.com/

3
https://www.ncbi.nlm.nih.gov/guide/all/
