# Supplementary material for: The screening for marine fungal strains with high potential in alkaloids production by in situ colony assay and LC-MS/MS based secondary metabolic profiling
Source: Front Microbiol. 2023 May 3;14:1144328. doi: 10.3389/fmicb.2023.1144328 (PMC10191116; doi:10.3389/fmicb.2023.1144328)
Supplement: Supplementary file 1 [file Data_Sheet_1.docx]

Supplementary Material

The Screening for Marine Fungi Strains with High Potential in Alkaloids Production by In Situ Colony Assay and LC-MS/MS Based Secondary Metabolic Profiling

Tiantian Lu^1#^, Yayue Liu^1,2,3#^, Longjian Zhou^1,2,3^, Yingying Nie^1^, Qingnan Liao^1^, Xingyuan Wang^1^, Xiaoling Lei^1,2,3^, Pengzhi Hong^1,2,3^, Yan Feng^1^, Xueqiong Hu^1,2,3^, Yi Zhang^1,2,3*^

*** Correspondence:** Prof. Dr. Yi Zhang : hubeizhangyi@163.com & zhangyi@gdou.edu.cn

**Figure legends**

FIGURE S1 The representative negative and weakly positive results in the in situ colony assay. A1 (S6-3 Y2) and A2 (S7-1-1) were negative results, while A3 (C23-3) was weakly positive result.

FIGURE S2 The annotations on the compounds with high yields and high scores in FBMN.

TABLE S1 Culture medium formulae used in the experiment

| Medium | Formula |
| --- | --- |
| Czapek–Dox Broth (CDB) | 3.0 g/L NaNO_3_, 1.0 g/L K_2_HPO_4_, 0.5 g/L MgSO_4_, 0.5 g/L KCl, 0.01 g/L FeSO_4_, 30.0 g/L sucrose, and 20.0 g/L sea salt. |
| Czapek–Dox Agar (CDA) | 3.0 g/L NaNO_3_, 1.0 g/L K_2_HPO_4_, 0.5 g/L MgSO_4_, 0.5 g/L KCl, 0.01 g/L FeSO_4_, 30.0 g/L sucrose, 20.0 g/L sea salt, and 15.0 g/L agar. |
| Potato Sucrose Broth (PSB) | 20.0 g/L potato leaching powder, 20.0 g/L sucrose, 5.0 g/L peptone, and 20.0 g/L sea salt. |
| Malt Extract Broth (MEB) | 20.0 g/L malt extract and 20.0 g/L sea salt. |
| Brown Rice Medium (BRM) | 800 g/L brown rice and 20.0 g/L sea salt. |
| Potato Dextrose Agar (PDA) | 20.0 g/L potato leaching powder, 20.0 g/L dextrose, 5.0 g/L peptone, 20.0 g/L sea salt, and 15.0 g/L agar. |
| Blakeslee’s Malt Extract Agar (MEA) | 30.0 g/L malt extract, 3.0 g/L peptone, 20.0 g/L sea salt, and 15.0 g/L agar. |
| Czapek Yeast Agar (CYA) | 3.0 g/L NaNO_3_, 1.0 g/L K_2_HPO_4_, 0.5 g/L MgSO_4_, 0.5 g/L KCl, 0.01 g/L FeSO_4_, 30.0 g/L sucrose, 5.0 g/L yeast extract, 20.0 g/L sea salt, and 15.0 g/L agar. |
| Yeast Extract Sucrose Agar (YES) | 10.0 g/L sucrose, 10.0 g/L yeast extract, 20.0 g/L sea salt, and 15.0 g/L agar. |

TABLE S2 Multiple database annotation results of the compounds in the same network with the azaphilones in Networks 5, 7, 9, 12, and 13

| No | Parent mass | RT | Area | Adduct | Formula |  | FBMN | | | | |  | MSFINDER Annotation TOP 5 | | | |  | CFM-ID 3.0 | | | | |  | Final Annotation |
| --- | --- | --- | --- | --- | --- | --- | --- | --- | --- | --- | --- | --- | --- | --- | --- | --- | --- | --- | --- | --- | --- | --- | --- | --- |
|  |  |  |  |  |  |  | Name | Smiles | MQ Score | Source | Network |  | Name | Smiles | Score | Source |  | Name | Smiles | Score | Formula | Source |  |  |
| 1 | 482.2052 | 5.66 | 2958 | [M+H]^+^ | C_27_H_23_N_5_O_4_ |  | **—** | **—** | **—** | **—** | 5 |  | **—** | **—** | **—** | **—** |  | *N*-[4-oxo-2-(2*H*-tetrazol-5-yl)chromen-7-yl]-4-(4-phenylbutoxy)benzamide | O=C(NC1=CC2=C(C=C1)C(=O)C=C(O2)C1=NNN=N1)C1=CC=C(OCCCCC2=CC=CC=C2)C=C1 | 0.69 | C_27_H_23_N_5_O_4_ | PubChem=115100, not found |  | *N*-[4-oxo-2-(2*H*-tetrazol-5-yl)chromen-7-yl]-4-(4-phenylbutoxy)benzamide |
| 2 | 498.1754 | 5.66 | 1760 | [M+H]^+^ | C_26_H_27_NO_9_ |  | **—** | **—** | **—** | **—** | 5 |  | **—** | **—** | **—** | **—** |  | 9-acetyl-7-(4-amino-5-hydroxy-6-methyloxan-2-yl)oxy-6,9,11-trihydroxy-8,10-dihydro-7*H*-tetracene-5,12-dione | CC1C(C(CC(O1)OC2CC(CC3=C2C(=C4C(=C3O)C(=O)C5=CC=CC=C5C4=O)O)(C(=O)C)O)N)O | 1 | C_26_H_27_NO_9_ | Plantae |  | 9-acetyl-7-(4-amino-5-hydroxy-6-methyloxan-2-yl)oxy-6,9,11-trihydroxy-8,10-dihydro-7*H*-tetracene-5,12-dione |
| 3 | 393.1285 | 5.57 | 2713 | [M+H]^+^ | C_22_H_20_N_2_O_3_S |  | **—** | **—** | **—** | **—** | 5 |  | 3-(1,3-benzothiazol-2-yl)-7-hydroxy-8-(piperidin-1-ylmethyl)chromen-2-one | O=C1OC=2C(=CC=C(O)C2CN3CCCCC3)C=C1C4=NC=5C=CC=CC5S4 | 3.69 | COCONUT=CNP0447354, not found |  | **—** | **—** | **—** | **—** | **—** |  | 3-(1,3-benzothiazol-2-yl)-7-hydroxy-2-methyl-8-(pyrrolidin-1-ylmethyl)chromen-4-one |
|  |  |  |  |  |  |  |  |  |  |  |  |  | 3-(1,3-benzothiazol-2-yl)-7-hydroxy-8-(piperidin-1-ylmethyl)-2*H*-chromen-2-one | O=C1C(=COC=2C1=CC=C(O)C2CN3CCCCC3)C4=NC=5C=CC=CC5S4 | 3.59 | COCONUT=CNP0443371, not found |  |  |  |  |  |  |  |  |

TABLE S2 cont.

| No | Parent mass | RT | Area | Adduct | Formula |  | FBMN | | | | |  | MSFINDER Annotation TOP 5 | | | |  | CFM-ID 3.0 | | | | |  | Final Annotation |
| --- | --- | --- | --- | --- | --- | --- | --- | --- | --- | --- | --- | --- | --- | --- | --- | --- | --- | --- | --- | --- | --- | --- | --- | --- |
|  |  |  |  |  |  |  | Name | Smiles | MQ Score | Source | Network |  | Name | Smiles | Score | Source |  | Name | Smiles | Score | Formula | Source |  |  |
|  |  |  |  |  |  |  |  |  |  |  |  |  | 3-(1,3-benzothiazol-2-yl)-7-hydroxy-2-methyl-8-(pyrrolidin-1-ylmethyl)chromen-4-one (*) | O=C1C2=CC=C(O)C(=C2OC(=C1C3=NC=4C=CC=CC4S3)C)CN5CCCC5 (*) | 3.59 | COCONUT=CNP0326739, not found |  |  |  |  |  |  |  |  |
|  |  |  |  |  |  |  |  |  |  |  |  |  | [4-(1,3-benzothiazol-2-yl)piperidin-1-yl]-(7-methoxy-1-benzofuran-2-yl)methanone | O=C(C=1OC=2C(OC)=CC=CC2C1)N3CCC(C4=NC=5C=CC=CC5S4)CC3 | 3.48 | COCONUT=CNP0070887, not found |  |  |  |  |  |  |  |  |
|  |  |  |  |  |  |  |  |  |  |  |  |  | 4-[(5-naphthalen-1-yl-1,3,4-oxadiazol-2-yl)sulfanyl]but-2-ynyl cyclopentanecarboxylate | O=C(OCC#CCSC1=NN=C(O1)C=2C=CC=C3C=CC=CC32)C4CCCC4 | 3.46 | ChEBI=CHEBI:93687, not found |  |  |  |  |  |  |  |  |
| 4 | 419.1477 | 5.59 | 4976 | [M+K]^+^ | C_20_H_28_O_7_ |  | **—** | **—** | **—** | **—** | 5 |  | Argophyllin A | O=C(OC1CC2(OC2CC(O)C(O)(C)CC3OC(=O)C(=C)C31)C)C(=CC)C | 3.73 | Plantae |  | **—** | **—** | **—** | **—** | **—** |  | Argophyllin A |
|  |  |  |  |  |  |  |  |  |  |  |  |  | Chaparrin | O=C1OC2CC3C(=CC(O)C(O)C3(C)C4C5(O)OCC24C(C1)C(C)C5O)C | 3.73 | Plantae |  |  |  |  |  |  |  |  |
|  |  |  |  |  |  |  |  |  |  |  |  |  | Cinncassiol C | O=C1C=C(C(C)CO)C2(C(=O)C34OC2(O)CC1(C)C4(O)CCC(C)C3O)C | 3.73 | COCONUT=CNP0247683, not found |  |  |  |  |  |  |  |  |
|  |  |  |  |  |  |  |  |  |  |  |  |  | Neurolenin C | O=C1OC2CC(C=CC(=O)C(O)(C)C(OC(=O)CC(C)C)C(O)C2C1=C)C | 3.73 | Plantae |  |  |  |  |  |  |  |  |
|  |  |  |  |  |  |  |  |  |  |  |  |  | Neurolenin D | O=C1OC2CC(C=CC(=O)C(O)(C)C(O)C(OC(=O)CC(C)C)C2C1=C)C | 3.72 | Plantae |  |  |  |  |  |  |  |  |

TABLE S2 cont.

| No | Parent mass | RT | Area | Adduct | Formula |  | FBMN | | | | |  | MSFINDER Annotation TOP 5 | | | |  | CFM-ID 3.0 | | | | |  | Final Annotation |
| --- | --- | --- | --- | --- | --- | --- | --- | --- | --- | --- | --- | --- | --- | --- | --- | --- | --- | --- | --- | --- | --- | --- | --- | --- |
|  |  |  |  |  |  |  | Name | Smiles | MQ Score | Source | Network |  | Name | Smiles | Score | Source |  | Name | Smiles | Score | Formula | Source |  |  |
| 5 | 458.2535 | 5.66 | 1748 | [M+NH_4_]^+^ | C_26_H_32_O_6_ |  | **—** | **—** | **—** | **—** | 5 |  | Tropolactone C | O=C1OC(C)(C)C2CCC3(OC=4C(=CC(=C(C(=O)OC)C(=O)C4C)C)CC3C2(C=C1)C)C | 4.75 | Fungi |  | **—** | **—** | **—** | **—** | **—** |  | Tropolactone C |
|  |  |  |  |  |  |  |  |  |  |  |  |  | 3-[2-(2,2-dimethyl-6-methylidenecyclohexyl)ethyl]-4-hydroxy-2-(hydroxymethyl)-7-methoxy-3-methyl-2*H*-benzo[g][1]benzofuran-6,9-dione | O=C1C=C(OC)C(=O)C2=CC(O)=C3C(OC(CO)C3(C)CCC4C(=C)CCCC4(C)C)=C12 | 4.69 | Bacterium |  |  |  |  |  |  |  |  |
|  |  |  |  |  |  |  |  |  |  |  |  |  | Kleinioxanthrone 1 | O=C(OC1C=2C=C(OC)C=C(O)C2C(=O)C3=C(O)C=C(C=C31)C)CCCCCCCCC | 4.69 | Plantae |  |  |  |  |  |  |  |  |
|  |  |  |  |  |  |  |  |  |  |  |  |  | 7-Deacetylgedunin (*) | O=C1OC(C2=COC=C2)C3(C)CCC4C5(C=CC(=O)C(C)(C)C5CC(O)C4(C)C63OC16)C (*) | 4.66 | Plantae |  |  |  |  |  |  |  |  |
|  |  |  |  |  |  |  |  |  |  |  |  |  | (1*S*,2*R*,4*S*,7*S*,8*S*,12*R*,19*R*)-7-(3-furyl)-19-hydroxy-1,8,12,16,16-pentamethyl-3,6-dioxapentacyclo[9.8.0.02,4.02,8.012,17]nonadec-13-ene-5,15-quinone | O=C1OC(C2=COC=C2)C3(C)CCC4C5(C=CC(=O)C(C)(C)C5CC(O)C4(C)C63OC16)C | 4.66 | Plantae |  |  |  |  |  |  |  |  |

TABLE S2 cont.

| No | Parent mass | RT | Area | Adduct | Formula |  | FBMN | | | | |  | | MSFINDER Annotation TOP 5 | | | | |  | | CFM-ID 3.0 | | | | | |  | Final Annotation |
| --- | --- | --- | --- | --- | --- | --- | --- | --- | --- | --- | --- | --- | --- | --- | --- | --- | --- | --- | --- | --- | --- | --- | --- | --- | --- | --- | --- | --- |
|  |  |  |  |  |  |  | Name | Smiles | MQ Score | Source | Network |  | Name | | Smiles | Score | Source |  | | Name | | Smiles | Score | Formula | Source |  | |  |
| 6 | 372.2103 | 5.65 | 8462 | [M+H]^+^ | C_19_H_25_N_5_O_3_ |  | **—** | **—** | **—** | **—** | 5 |  | **—** | | **—** | **—** | **—** |  | | 9-butyl-8-[(3,4,5-trimethoxyphenyl)methyl]purin-6-amine | | CCCCN1C(CC2=CC(OC)=C(OC)C(OC)=C2)=NC2=C(N)N=CN=C12 | 0.82 | C_19_H_25_N_5_O_3_ | PubChem=448965, not found |  | | 9-butyl-8-[(3,4,5-trimethoxyphenyl)methyl]purin-6-amine |
| 7 | 374.2107 | 5.65 | 2075 | [M+H]^+^ | C_22_H_28_FNO_3_ |  | **—** | **—** | **—** | **—** | 5 |  | 5-[[(4-fluorophenyl)methylamino]methyl]-10,14-dimethyl-2,7-dioxatetracyclo[8.4.0.01,3.04,8]tetradecan-6-one | | O=C1OC2CC3(C)CCCC(C)C43OC4C2C1CNCC5=CC=C(F)C=C5 | 3.91 | COCONUT=CNP0252816, not found |  | | 3,4,11,12-tetramethoxy-17-methyl-17-azatetracyclo[8.4.3.01,10.02,7]heptadeca-2(7),3,5,11-tetraen-13-one | | CN1CCC23C1(CCC4=C2C(=C(C=C4)OC)OC)C(=C(C(=O)C3)OC)OC | 0.73 | C_21_H_27_NO_5_ | Plantae |  | | 5-[[(4-fluorophenyl)methylamino]methyl]-10,14-dimethyl-2,7-dioxatetracyclo[8.4.0.01,3.04,8]tetradecan-6-one |
|  |  |  |  |  |  |  |  |  |  |  |  |  | *N*-(3,5a,9-trimethyl-2-oxo-3,3a,4,5,6,7,8,9,9a,9b-decahydrobenzo[g][1]benzofuran-8-yl)-3-fluorobenzamide | | O=C1OC2C(CCC3(C)CCC(NC(=O)C=4C=CC=C(F)C4)C(C)C23)C1C | 3.87 | COCONUT=CNP0223677, not found |  | |  | |  |  |  |  |  | |  |
|  |  |  |  |  |  |  |  |  |  |  |  |  | 3-[[(4-fluorophenyl)methylamino]methyl]-8a-methylspiro[3a,4,4a,6,7,8,9,9a-octahydro-3*H*-benzo[f][1]benzofuran-5,2'-oxirane]-2-one | | O=C1OC2CC3(C)CCCC4(OC4)C3CC2C1CNCC5=CC=C(F)C=C5 | 3.86 | COCONUT=CNP0388726, not found |  | |  | |  |  |  |  |  | |  |

TABLE S2 cont.

| No | Parent mass | RT | Area | Adduct | Formula |  | FBMN | | | | |  | MSFINDER Annotation TOP 5 | | | |  | CFM-ID 3.0 | | | | |  | Final Annotation |
| --- | --- | --- | --- | --- | --- | --- | --- | --- | --- | --- | --- | --- | --- | --- | --- | --- | --- | --- | --- | --- | --- | --- | --- | --- |
|  |  |  |  |  |  |  | Name | Smiles | MQ Score | Source | Network |  | Name | Smiles | Score | Source |  | Name | Smiles | Score | Formula | Source |  |  |
| 8 | 391.1307 | 5.59 | 12704 | [M+H]^+^ | C_21_H_23_ClO_5_ |  | **—** | **—** | **—** | **—** | 5 |  | Sclerotiorin (*) | O=C(OC1(C(=O)C(Cl)=C2C=C(OC=C2C1=O)C=CC(=CC(C)CC)C)C)C (*) | 4.81 | Fungi |  | **—** | **—** | **—** | **—** | **—** |  | Sclerotiorin |
|  |  |  |  |  |  |  |  |  |  |  |  |  | methyl 9-(3-chloroprop-1-en-2-yl)-5-oxo-2-prop-1-en-2-yl-4,14-dioxatricyclo[9.2.1.13,6]pentadeca-1(13),6(15),11-triene-12-carboxylate | O=C(OC)C=1C=C2OC1CC(C(=C)CCl)CCC3=CC(OC3=O)C2C(=C)C | 4.58 | Metazoa |  |  |  |  |  |  |  |  |
|  |  |  |  |  |  |  |  |  |  |  |  |  | 3-(6-chloro-4*H*-1,3-benzodioxin-8-yl)-1-(4-ethoxy-2-hydroxyphenyl)-2-methylbutan-1-one | O=C(C1=CC=C(OCC)C=C1O)C(C)C(C=2C=C(Cl)C=C3C2OCOC3)C | 4.26 | COCONUT=CNP0196306, not found |  |  |  |  |  |  |  |  |
| 9 | 415.1104 | 5.57 | 4001 | [M+H]^+^ | C_25_H_18_O_6_ |  | **—** | **—** | **—** | **—** | 5 |  | 6-acetyl-5-hydroxy-4-methyl-10-naphthalen-1-yl-9,10-dihydropyrano[2,3-h]chromene-2,8-dione | O=C1OC2=C(C(O)=C(C(=O)C)C=3OC(=O)CC(C4=CC=CC=5C=CC=CC54)C23)C(=C1)C | 3.94 | COCONUT=CNP0383438, not found |  | **—** | **—** | **—** | **—** | **—** |  | 6-acetyl-5-hydroxy-4-methyl-10-naphthalen-1-yl-9,10-dihydropyrano[2,3-h]chromene-2,8-dione |
|  |  |  |  |  |  |  |  |  |  |  |  |  | 5-hydroxy-10-(4-methoxyphenyl)-3-phenyl-9,10-dihydropyrano[2,3-h]chromene-4,8-dione | O=C1OC2=CC(O)=C3C(=O)C(=COC3=C2C(C4=CC=C(OC)C=C4)C1)C=5C=CC=CC5 | 3.93 | Plantae |  |  |  |  |  |  |  |  |
|  |  |  |  |  |  |  |  |  |  |  |  |  | 5-hydroxy-10-(2-methoxyphenyl)-2-phenyl-9,10-dihydropyrano[2,3-h]chromene-4,8-dione | O=C1C=C(OC=2C1=C(O)C=C3OC(=O)CC(C=4C=CC=CC4OC)C32)C=5C=CC=CC5 | 3.93 | COCONUT=CNP0402176, not found |  |  |  |  |  |  |  |  |
|  |  |  |  |  |  |  |  |  |  |  |  |  | Interruptin D | O=C1OC=2C=3C(=O)C(O)(OC3C(=C(O)C2C(=C1)C=4C=CC=CC4)C)CC=5C=CC=CC5 | 3.93 | Archaeplastida |  |  |  |  |  |  |  |  |

TABLE S2 cont.

| No | Parent mass | RT | Area | Adduct | Formula |  | FBMN | | | | |  | MSFINDER Annotation TOP 5 | | | |  | CFM-ID 3.0 | | | | |  | Final Annotation |
| --- | --- | --- | --- | --- | --- | --- | --- | --- | --- | --- | --- | --- | --- | --- | --- | --- | --- | --- | --- | --- | --- | --- | --- | --- |
|  |  |  |  |  |  |  | Name | Smiles | MQ Score | Source | Network |  | Name | Smiles | Score | Source |  | Name | Smiles | Score | Formula | Source |  |  |
|  |  |  |  |  |  |  |  |  |  |  |  |  | 5-hydroxy-3-(4-hydroxyphenyl)-10-(4-methylphenyl)-9,10-dihydropyrano[2,3-h]chromene-4,8-dione | O=C1OC2=CC(O)=C3C(=O)C(=COC3=C2C(C4=CC=C(C=C4)C)C1)C=5C=CC(O)=CC5 | 3.92 | COCONUT=CNP0401883, not found |  |  |  |  |  |  |  |  |
| 10 | 417.1475 | 5.62 | 22303 | [M+Na]^+^ | C_21_H_27_ClO_5_ |  | **—** | **—** | **—** | **—** | 5 |  | Isochromophilone VIII (*) | O=C(OC1C2C(C=C(OC2)C=CC(=CC(C)CC)C)=C(Cl)C(=O)C1(O)C)C (*) | 4.35 | Fungi |  | **—** | **—** | **—** | **—** | **—** |  | Isochromophilone VIII |
|  |  |  |  |  |  |  |  |  |  |  |  |  | Bisphenol A (3-chloro-2-hydroxypropyl) (2,3-dihydroxypropyl) ether | ClCC(O)COC1=CC=C(C=C1)C(C2=CC=C(OCC(O)CO)C=C2)(C)C | 4.23 | COCONUT=CNP0254775, not found |  |  |  |  |  |  |  |  |
|  |  |  |  |  |  |  |  |  |  |  |  |  | Loteprednol | O=C1C=CC2(C(=C1)CCC3C4CCC(O)(C(=O)OCCl)C4(C)CC(O)C32)C | 4.15 | ChEBI=CHEBI:50848, not found |  |  |  |  |  |  |  |  |
|  |  |  |  |  |  |  |  |  |  |  |  |  | 8a-acetyl-5-chloro-3-(3,5-dimethylhepta-1,3-dienyl)-7,8-dihydroxy-7-methyl-1,8-dihydroisochromen-6-one | O=C1C(Cl)=C2C=C(OCC2(C(=O)C)C(O)C1(O)C)C=CC(=CC(C)CC)C | 4.14 | Fungi |  |  |  |  |  |  |  |  |
|  |  |  |  |  |  |  |  |  |  |  |  |  | [5-chloro-3-(3,5-dimethylheptyl)-7-methyl-6,8-dioxoisochromen-7-yl] acetate | O=C(OC1(C(=O)C(Cl)=C2C=C(OC=C2C1=O)CCC(C)CC(C)CC)C)C | 3.92 | Fungi |  |  |  |  |  |  |  |  |

TABLE S2 cont.

| No | Parent mass | RT | Area | Adduct | Formula |  | FBMN | | | | |  | MSFINDER Annotation TOP 5 | | | |  | CFM-ID 3.0 | | | | |  | Final Annotation |
| --- | --- | --- | --- | --- | --- | --- | --- | --- | --- | --- | --- | --- | --- | --- | --- | --- | --- | --- | --- | --- | --- | --- | --- | --- |
|  |  |  |  |  |  |  | Name | Smiles | MQ Score | Source | Network |  | Name | Smiles | Score | Source |  | Name | Smiles | Score | Formula | Source |  |  |
| 11 | 462.2221 | 5.66 | 3290 | [M+H]^+^ | C_23_H_32_ClN_5_O_3_ |  | **—** | **—** | **—** | **—** | 5 |  | **—** | **—** | **—** | **—** |  | 2-ethylbutyl (3*S*,4a*S*,6*S*,8a*R*)-6-[3-chloro-2-(2*H*-tetrazol-5-yl)phenoxy]-1,2,3,4,4a,5,6,7,8,8a-decahydroisoquinoline-3-carboxylate | CCC(CC)COC(=O)C1CC2CC(CCC2CN1)OC3=C(C(=CC=C3)Cl)C4=NNN=N4 | 0.67 | C_23_H_32_ClN_5_O_3_ | PubChem=53307207, not found |  | 2-ethylbutyl (3*S*,4a*S*,6*S*,8a*R*)-6-[3-chloro-2-(2*H*-tetrazol-5-yl)phenoxy]-1,2,3,4,4a,5,6,7,8,8a-decahydroisoquinoline-3-carboxylate |
| 12 | 413.1157 | 5.59 | 16645 | [M+Na]^+^ | C_20_H_22_O_8_ |  | **—** | **—** | **—** | **—** | 5 |  | Piceid | OC1=CC=C(C=C1)C=CC=2C=C(O)C=C(OC3OC(CO)C(O)C(O)C3O)C2 | 5.29 | Plantae |  | **—** | **—** | **—** | **—** | **—** |  | Cordyanhydride A |
|  |  |  |  |  |  |  |  |  |  |  |  |  | Cordyanhydride A | O=C1OC(=O)C(=C1C=CCC)CC(CC=2C(=O)OC(=O)C2CCC(=O)O)CC | 5.2 | Fungi |  |  |  |  |  |  |  |  |
|  |  |  |  |  |  |  |  |  |  |  |  |  | Jamesoniellide K | O=C1OC(C2=CC(O)OC2=O)CC(=C1CC3OC(=O)C4=CCCC(O)C43C)C | 5.13 | Plantae |  |  |  |  |  |  |  |  |
|  |  |  |  |  |  |  |  |  |  |  |  |  | Jamesoniellide L | O=C1OC(O)C(=C1)C2OC(=O)C(=C(C)C2)CC3OC(=O)C4=CCCC(O)C43C | 5.11 | Plantae |  |  |  |  |  |  |  |  |
|  |  |  |  |  |  |  |  |  |  |  |  |  | Resveratroloside | OC=1C=C(O)C=C(C=CC2=CC=C(OC3OC(CO)C(O)C(O)C3O)C=C2)C1 | 5.11 | Plantae |  |  |  |  |  |  |  |  |

TABLE S2 cont.

| No | Parent mass | RT | Area | Adduct | Formula |  | FBMN | | | | |  | | MSFINDER Annotation TOP 5 | | | | |  | | CFM-ID 3.0 | | | | | |  | Final Annotation |
| --- | --- | --- | --- | --- | --- | --- | --- | --- | --- | --- | --- | --- | --- | --- | --- | --- | --- | --- | --- | --- | --- | --- | --- | --- | --- | --- | --- | --- |
|  |  |  |  |  |  |  | Name | Smiles | MQ Score | Source | Network |  | Name | | Smiles | Score | Source |  | | Name | | Smiles | Score | Formula | Source |  | |  |
| 13 | 460.2269 | 5.66 | 13012 | [M+Na]^+^ | C_25_H_31_N_3_O_4_ |  | **—** | **—** | **—** | **—** | 5 |  | Rubrumline E | | O=C1NC(=CC=2C=3C=C(C=CC3NC2C(C=C)(C)C)CC(O)C(OC)(C)C)C(=O)NC1=C | 3.92 | Fungi |  | | **—** | | **—** | **—** | **—** | **—** |  | | Rubrumline E |
|  |  |  |  |  |  |  |  |  |  |  |  |  | Lunarine (*) | | O=C1C=CC=2C=CC=3OC4CC(=O)CCC4(C=CC(=O)NCCCCNCCCN1)C3C2 (*) | 3.87 | Plantae |  | |  | |  |  |  |  |  | |  |
|  |  |  |  |  |  |  |  |  |  |  |  |  | 5,14-dihydroxy-6,15-diphenyl-tetradecahydro-1*H*-pyrrolo[1,2-a]1,5,9-triazacyclotridecane-4,13-dione | | O=C1NCCC2N(C(=O)C(O)C2C=3C=CC=CC3)CCCCNC(C=4C=CC=CC4)C1O | 3.86 | Archaeplastida |  | |  | |  |  |  |  |  | |  |
|  |  |  |  |  |  |  |  |  |  |  |  |  | 3-(4-hydroxyphenyl)-*N*-[4-[3-[3-(4-hydroxyphenyl)prop-2-enoylamino]propylamino]butyl]prop-2-enamide | | O=C(C=CC1=CC=C(O)C=C1)NCCCNCCCCNC(=O)C=CC2=CC=C(O)C=C2 | 3.78 | Archaeplastida |  | |  | |  |  |  |  |  | |  |
|  |  |  |  |  |  |  |  |  |  |  |  |  | 1-{2-[({[1,1'-biphenyl]-4-yl}methyl)amino]-3-hydroxy-6,8-dioxabicyclo[3.2.1]octan-4-yl}piperidine-4-carboxamide | | O=C(N)C1CCN(CC1)C2C3OCC(O3)C(NCC=4C=CC(=CC4)C=5C=CC=CC5)C2O | 3.77 | COCONUT=CNP0012553, not found |  | |  | |  |  |  |  |  | |  |
| 14 | 439.1299 | 5.62 | 12166 | [M+Na]^+^ | C_22_H_24_O_8_ |  | **—** | **—** | **—** | **—** | 5 |  | 1-Acetoxypinoresinol | | O=C(OC12COC(C3=CC=C(O)C(OC)=C3)C2COC1C4=CC=C(O)C(OC)=C4)C | 4.59 | Plantae |  | | Syncurine | | [Br-].[Br-].C[N+](C)(C)CCCCCCCCCC[N+](C)(C)C | 0.91 | C_16_H_38_N_2_.2BrH | PubChem=10921, not found |  | | Comazaphilone E |

TABLE S2 cont.

| No | Parent mass | RT | Area | Adduct | Formula |  | FBMN | | | | |  | | MSFINDER Annotation TOP 5 | | | | |  | | CFM-ID 3.0 | | | | |  | Final Annotation |
| --- | --- | --- | --- | --- | --- | --- | --- | --- | --- | --- | --- | --- | --- | --- | --- | --- | --- | --- | --- | --- | --- | --- | --- | --- | --- | --- | --- |
|  |  |  |  |  |  |  | Name | Smiles | MQ Score | Source | Network |  | Name | | Smiles | Score | Source |  | | Name | | Smiles | Score | Formula | Source |  |  |
|  |  |  |  |  |  |  |  |  |  |  |  |  | Comazaphilone E | | O=C(OC1CC=2C=C(OCC2C(=O)C1(O)C)C=CC)C3=C(OC)C(O)=C(O)C=C3C | 4.58 | Fungi |  | |  | |  |  |  |  |  |  |
|  |  |  |  |  |  |  |  |  |  |  |  |  | Alnumycin | | O=C1C=C(C(=O)C2=C(O)C3=C(C=C(OC3CCC)C)C=C12)C4OCC(O)C(O4)CO | 4.55 | Bacterium |  | |  | |  |  |  |  |  |  |
| 15 | 431.088 | 5.57 | 1972 | [M+Na]^+^ | C_19_H_20_O_10_ |  | **—** | **—** | **—** | **—** | 5 |  | 4-{[3-(4-hydroxy-3-methoxyphenyl)prop-2-enoyl]oxy}-2-(methoxycarbonyl)-7-oxooxepane-4-carboxylic acid | | O=C(OC1(C(=O)O)CCC(=O)OC(C(=O)OC)C1)C=CC2=CC=C(O)C(OC)=C2 | 4.34 | COCONUT=CNP0287981, not found |  | | **—** | | **—** | **—** | **—** | **—** |  | 4-{[3-(4-hydroxy-3-methoxyphenyl)prop-2-enoyl]oxy}-2-(methoxycarbonyl)-7-oxooxepane-4-carboxylic acid |
|  |  |  |  |  |  |  |  |  |  |  |  |  | 4-hydroxy-6-[2-(4-hydroxyphenyl)ethenyl]-3-{[3,4,5-trihydroxy-6-(hydroxymethyl)oxan-2-yl]oxy}-2*H*-pyran-2-one | | O=C1OC(C=CC2=CC=C(O)C=C2)=CC(O)=C1OC3OC(CO)C(O)C(O)C3O | 4.31 | Archaeplastida |  | |  | |  |  |  |  |  |  |
|  |  |  |  |  |  |  |  |  |  |  |  |  | Khellol glucoside | | O=C1C=C(OC=2C=C3OC=CC3=C(OC)C12)COC4OC(CO)C(O)C(O)C4O | 4.30 | Plantae |  | |  | |  |  |  |  |  |  |
|  |  |  |  |  |  |  |  |  |  |  |  |  | Fortuneanoside L | | OC1=CC=CC=2OC=3C(OC4OC(CO)C(O)C(O)C4O)=C(O)C(OC)=CC3C12 | 4.22 | Plantae |  | |  | |  |  |  |  |  |  |
|  |  |  |  |  |  |  |  |  |  |  |  |  | {3,4,5-trihydroxy-6-[(2-methyl-4-oxo-4*H*-pyran-3-yl)oxy]oxan-2-yl}methyl 4-hydroxybenzoate | | O=C(OCC1OC(OC=2C(=O)C=COC2C)C(O)C(O)C1O)C3=CC=C(O)C=C3 | 4.22 | Plantae |  | |  | |  |  |  |  |  |  |

TABLE S2 cont.

| No | Parent mass | RT | Area | Adduct | Formula |  | FBMN | | | | |  | MSFINDER Annotation TOP 5 | | | |  | | CFM-ID 3.0 | | | | |  | Final Annotation |
| --- | --- | --- | --- | --- | --- | --- | --- | --- | --- | --- | --- | --- | --- | --- | --- | --- | --- | --- | --- | --- | --- | --- | --- | --- | --- |
|  |  |  |  |  |  |  | Name | Smiles | MQ Score | Source | Network |  | Name | Smiles | Score | Source |  | Name | | Smiles | Score | Formula | Source |  |  |
| 16 | 412.1305 | 4.29 | 4634 | [M+H]^+^ | C_23_H_22_ClNO_4_ |  | **—** | **—** | **—** | **—** | 7 |  | *N*-(2-chlorophenyl)-2-{4,8,8-trimethyl-2-oxo-2*H*,6*H*,7*H*,8*H*-pyrano[3,2-g]chromen-3-yl}acetamide | O=C1OC=2C=C3OC(C)(C)CCC3=CC2C(=C1CC(=O)NC=4C=CC=CC4Cl)C | 4.33 | COCONUT=CNP0412133, not found |  | 3-(1,1-dioxo-4*H*-1lambda6,2,4-benzothiadiazin-3-yl)-4-hydroxy-1-(3-methylbutyl)quinolin-2-one | | CC(C)CCN1C(=O)C(C2=NS(=O)(=O)C3=CC=CC=C3N2)=C(O)C2=CC=CC=C12 | 0.63 | C_21_H_21_N_3_O_4_S | Drug Bank=DB07275, not found |  | N-(2-chlorophenyl)-2-{4,8,8-trimethyl-2-oxo-2*H*,6*H*,7*H*,8*H*-pyrano[3,2-g]chromen-3-yl}acetamide |
|  |  |  |  |  |  |  |  |  |  |  |  |  | *N*-[2-(4-chlorobenzoyl)-7-methoxy-1-benzofuran-3-yl]cyclohexanecarboxamide | O=C(C1=CC=C(Cl)C=C1)C=2OC=3C(OC)=CC=CC3C2NC(=O)C4CCCCC4 | 4.27 | COCONUT=CNP0048863, not found |  |  | |  |  |  |  |  |  |
|  |  |  |  |  |  |  |  |  |  |  |  |  | 3-(4-chlorophenyl)-2-methyl-9-[(oxolan-2-yl)methyl]-4*H*,8*H*,9*H*,10*H*-chromeno[8,7-e][1,3]oxazin-4-one | O=C1C2=CC=C3OCN(CC3=C2OC(=C1C=4C=CC(Cl)=CC4)C)CC5OCCC5 | 4.26 | COCONUT=CNP0111114, not found |  |  | |  |  |  |  |  |  |
|  |  |  |  |  |  |  |  |  |  |  |  |  | 2-(4-chlorophenyl)-*N*-{2-[3-methoxy-4-(prop-2-yn-1-yloxy)phenyl]ethyl}-2-(prop-2-yn-1-yloxy)acetamide(*) | O=C(NCCC1=CC=C(OCC#C)C(OC)=C1)C(OCC#C)C2=CC=C(Cl)C=C2 (*) | 4.08 | COCONUT=CNP0347571, not found |  |  | |  |  |  |  |  |  |
| 17 | 498.1663 | 4.01 | 20131 | [M+Na]^+^ | C_25_H_30_ClNO_6_ |  | **—** | **—** | **—** | **—** | 7 |  | Chaetomugilide C | O=C1OC2(C(=O)C(Cl)=C3C=C(C=CC(C)CC)N(C=C3C2=C1C(=O)C(C)C(O)C)CCO)C | 5.75 | Fungi |  | **—** | | **—** | **—** | **—** | **—** |  | Penazaphilone I |

TABLE S2 cont.

| No | Parent mass | RT | Area | Adduct | Formula |  | FBMN | | | | |  | | MSFINDER Annotation TOP 5 | | | | |  | | CFM-ID 3.0 | | | | | |  | Final Annotation |
| --- | --- | --- | --- | --- | --- | --- | --- | --- | --- | --- | --- | --- | --- | --- | --- | --- | --- | --- | --- | --- | --- | --- | --- | --- | --- | --- | --- | --- |
|  |  |  |  |  |  |  | Name | Smiles | MQ Score | Source | Network |  | Name | | Smiles | Score | Source |  | | Name | | Smiles | Score | Formula | Source |  | |  |
|  |  |  |  |  |  |  |  |  |  |  |  |  | Penazaphilone I (*) | | O=C(OC1(C(=O)C(Cl)=C2C=C(C=CC(=CC(C)CC)C)N(C=C2C1=O)CCCC(=O)O)C)C (*) | 5.59 | Fungi |  | |  | |  |  |  |  |  | |  |
|  |  |  |  |  |  |  |  |  |  |  |  |  | Chaetoviridide C | | O=C1OC2(C(=O)C(Cl)=C3C=C(C=CC(C)CC)N(C=C3C2=C1C(=O)CC)CCOCCO)C | 5.55 | Fungi |  | |  | |  |  |  |  |  | |  |
|  |  |  |  |  |  |  |  |  |  |  |  |  | 4-[(2-{6-chloro-4-methyl-7-[(3-methylbut-2-en-1-yl)oxy]-2-oxo-2*H*-chromen-3-yl}acetamido)methyl]cyclohexane-1-carboxylic acid | | O=C1OC=2C=C(OCC=C(C)C)C(Cl)=CC2C(=C1CC(=O)NCC3CCC(C(=O)O)CC3)C | 5.34 | COCONUT=CNP0391570, not found |  | |  | |  |  |  |  |  | |  |
| 18 | 412.1305 | 4.24 | 2973 | [M+H]^+^ | C_23_H_22_ClNO_4_ |  | **—** | **—** | **—** | **—** | 7 |  | 3-(4-chlorophenyl)-2-methyl-9-[(oxolan-2-yl)methyl]-4*H*,8*H*,9*H*,10*H*-chromeno[8,7-e][1,3]oxazin-4-one | | O=C1C2=CC=C3OCN(CC3=C2OC(=C1C=4C=CC(Cl)=CC4)C)CC5OCCC5 | 5.04 | COCONUT=CNP0111114, not found |  | | 3-(1,1-dioxo-4*H*-1lambda6,2,4-benzothiadiazin-3-yl)-4-hydroxy-1-(3-methylbutyl)quinolin-2-one | | CC(C)CCN1C(=O)C(C2=NS(=O)(=O)C3=CC=CC=C3N2)=C(O)C2=CC=CC=C12 | 0.63 | C_21_H_21_N_3_O_4_S | Drug Bank=DB07275, not found |  | | 3-(4-chlorophenyl)-2-methyl-9-[(oxolan-2-yl)methyl]-4*H*,8*H*,9*H*,10*H*-chromeno[8,7-e][1,3]oxazin-4-one |
|  |  |  |  |  |  |  |  |  |  |  |  |  | *N*-[2-(4-chlorobenzoyl)-7-methoxy-1-benzofuran-3-yl]cyclohexanecarboxamide | | O=C(C1=CC=C(Cl)C=C1)C=2OC=3C(OC)=CC=CC3C2NC(=O)C4CCCCC4 | 5.04 | COCONUT=CNP0048863, not found |  | |  | |  |  |  |  |  | |  |

TABLE S2 cont.

| No | Parent mass | RT | Area | Adduct | Formula |  | FBMN | | | | |  | | MSFINDER Annotation TOP 5 | | | | |  | | CFM-ID 3.0 | | | | | |  | Final Annotation |
| --- | --- | --- | --- | --- | --- | --- | --- | --- | --- | --- | --- | --- | --- | --- | --- | --- | --- | --- | --- | --- | --- | --- | --- | --- | --- | --- | --- | --- |
|  |  |  |  |  |  |  | Name | Smiles | MQ Score | Source | Network |  | Name | | Smiles | Score | Source |  | | Name | | Smiles | Score | Formula | Source |  | |  |
|  |  |  |  |  |  |  |  |  |  |  |  |  | *N*-(2-chlorophenyl)-2-{4,8,8-trimethyl-2-oxo-2*H*,6*H*,7*H*,8*H*-pyrano[3,2-g]chromen-3-yl}acetamide | | O=C1OC=2C=C3OC(C)(C)CCC3=CC2C(=C1CC(=O)NC=4C=CC=CC4Cl)C | 5.01 | COCONUT=CNP0412133, not found |  | |  | |  |  |  |  |  | |  |
|  |  |  |  |  |  |  |  |  |  |  |  |  | 2-(4-chlorophenyl)-*N*-{2-[3-methoxy-4-(prop-2-yn-1-yloxy)phenyl]ethyl}-2-(prop-2-yn-1-yloxy)acetamide (*) | | O=C(NCCC1=CC=C(OCC#C)C(OC)=C1)C(OCC#C)C2=CC=C(Cl)C=C2 (*) | 4.74 | COCONUT=CNP0347571, not found |  | |  | |  |  |  |  |  | |  |
| 19 | 520.1771 | 3.51 | 27278 | [M+H]^+^ | C_26_H_30_ClNO_8_ |  | 2-[7-(acetyloxy)-5-chloro-3-(3,5-dimethylhepta-1,3-dien-1-yl)-7-methyl-6,8-dioxo-2,6,7,8-tetrahydroisoquinolin-2-yl]pentanedioic acid | CCC(C)\C=C(C)\C=C\C1=C\C/2=C(Cl)/C(=O)C(C)(OC(C)=O)C(=O)C2=C\N1C(CCC(O)=O)C(O)=O | 0.86 | Fungi | 7 |  | 2-[5-chloro-7-(3,5-dihydroxyhexanoyloxy)-3-hepta-1,3,5-trienyl-7-methyl-6,8-dioxoisoquinolin-2-yl]propanoic acid | | O=C(O)C(N1C=C2C(=O)C(OC(=O)CC(O)CC(O)C)(C(=O)C(Cl)=C2C=C1C=CC=CC=CC)C)C | 4.66 | Fungi |  | | **—** | | **—** | **—** | **—** | **—** |  | | 2-[7-(acetyloxy)-5-chloro-3-(3,5-dimethylhepta-1,3-dien-1-yl)-7-methyl-6,8-dioxo-2,6,7,8-tetrahydroisoquinolin-2-yl]pentanedioic acid |
|  |  |  |  |  |  |  |  |  |  |  |  |  | 2-[7-(acetyloxy)-5-chloro-3-(3,5-dimethylhepta-1,3-dien-1-yl)-7-methyl-6,8-dioxo-2,6,7,8-tetrahydroisoquinolin-2-yl]pentanedioic acid (*) | | O=C(O)CCC(C(=O)O)N1C=C2C(=O)C(OC(=O)C)(C(=O)C(Cl)=C2C=C1C=CC(=CC(C)CC)C)C (*) | 4.64 | Fungi |  | |  | |  |  |  |  |  | |  |
| 20 | 522.1719 | 3.50 | 9043 | [M+Na]^+^ | C_26_H_29_NO_9_ |  | **—** | **—** | **—** | **—** | 7 |  | 13-Deoxycarminomycin | | O=C1C=2C=CC=C(O)C2C(=O)C=3C(O)=C4C(=C(O)C13)CC(O)(CC)CC4OC5OC(C)C(O)C(N)C5 | 4.66 | Bacterium |  | | Halofantrine | | CCCCN(CCCC)CCC(O)C1=C2C=CC(=CC2=C2C=C(Cl)C=C(Cl)C2=C1)C(F)(F)F | 0.32 | C_26_H_30_Cl_2_F_3_NO | PubChem=37393, not found |  | | 13-Deoxycarminomycin |

TABLE S2 cont.

| No | Parent mass | RT | Area | Adduct | Formula |  | FBMN | | | | |  | MSFINDER Annotation TOP 5 | | | |  | CFM-ID 3.0 | | | | |  | Final Annotation |
| --- | --- | --- | --- | --- | --- | --- | --- | --- | --- | --- | --- | --- | --- | --- | --- | --- | --- | --- | --- | --- | --- | --- | --- | --- |
|  |  |  |  |  |  |  | Name | Smiles | MQ Score | Source | Network |  | Name | Smiles | Score | Source |  | Name | Smiles | Score | Formula | Source |  |  |
|  |  |  |  |  |  |  |  |  |  |  |  |  | Chrotacumine C | O=C(OC1CN(C)CCC1C=2C(O)=CC(O)=C3C(=O)C=C(OC32)C)C4=CC(OC)=C(OC)C(OC)=C4 | 4.58 | Plantae |  |  |  |  |  |  |  |  |
|  |  |  |  |  |  |  |  |  |  |  |  |  | Oxaunomycin, 6-Deoxy | O=C1C=2C=C3C(=C(O)C2C(=O)C=4C=CC=C(O)C14)C(O)C(O)(CC)CC3OC5OC(C)C(O)C(N)C5 | 4.38 | Bacterium |  |  |  |  |  |  |  |  |
|  |  |  |  |  |  |  |  |  |  |  |  |  | 7-[(4-amino-5-hydroxy-6-methyloxan-2-yl)oxy]-6,9,11-trihydroxy-9-(1-hydroxyethyl)-5,7,8,9,10,12-hexahydrotetracene-5,12-dione | O=C1C=2C=CC=CC2C(=O)C=3C(O)=C4C(=C(O)C13)CC(O)(CC4OC5OC(C)C(O)C(N)C5)C(O)C | 4.37 | Bacterium |  |  |  |  |  |  |  |  |
|  |  |  |  |  |  |  |  |  |  |  |  |  | methyl 3-{1-[2-(3,4-dihydroxyphenyl)ethyl]-4-hydroxy-6-methyl-2-oxo-1,2-dihydropyridin-3-yl}-3-(4-hydroxy-3,5-dimethoxyphenyl)propanoate | O=C(OC)CC(C1=CC(OC)=C(O)C(OC)=C1)C2=C(O)C=C(N(C2=O)CCC3=CC=C(O)C(O)=C3)C | 4.28 | COCONUT=CNP0407670, not found |  |  |  |  |  |  |  |  |
| 21 | 390.1479 | 4.29 | 40553 | [M+H]^+^ | C_21_H_24_ClNO_4_ |  | Penazaphilone E | CCC(C)C=C(C)C=CC1=CC2=C(C=N1)C(=O)C(C(=C2Cl)O)(C)OC(=O)C | 0.77 | Fungi | 7 |  | Sclerotioramine (*) | O=C(OC1(C(=O)C(Cl)=C2C=C(C=CC(=CC(C)CC)C)NC=C2C1=O)C)C (*) | 5.39 | Fungi |  | **—** | **—** | **—** | **—** | **—** |  | Sclerotioramine |

TABLE S2 cont.

| No | Parent mass | RT | Area | Adduct | Formula |  | FBMN | | | | |  | MSFINDER Annotation TOP 5 | | | |  | CFM-ID 3.0 | | | | |  | Final Annotation |
| --- | --- | --- | --- | --- | --- | --- | --- | --- | --- | --- | --- | --- | --- | --- | --- | --- | --- | --- | --- | --- | --- | --- | --- | --- |
|  |  |  |  |  |  |  | Name | Smiles | MQ Score | Source | Network |  | Name | Smiles | Score | Source |  | Name | Smiles | Score | Formula | Source |  |  |
|  |  |  |  |  |  |  |  |  |  |  |  |  | Penazaphilone E | O=C(OC1(C(=O)C=2C=NC(C=CC(=CC(C)CC)C)=CC2C(Cl)=C1O)C)C | 5.32 | Fungi |  |  |  |  |  |  |  |  |
| 22 | 392.1432 | 4.26 | 17327 | [M+Na]^+^ | C_21_H_23_NO_5_ |  | **—** | **—** | **—** | **—** | 7 |  | Tenellin | O=C(C=CC(=CC(C)CC)C)C=1C(=O)N(O)C=C(C=2C=CC(O)=CC2)C1O | 5.71 | Fungi |  | **—** | **—** | **—** | **—** | **—** |  | Tenellin |
|  |  |  |  |  |  |  |  |  |  |  |  |  | Fumaricine | OC1C2=C3OCOC3=CC=C2CC41C5=CC(OC)=C(OC)C=C5CCN4C | 5.62 | Plantae |  |  |  |  |  |  |  |  |
|  |  |  |  |  |  |  |  |  |  |  |  |  | Azaspirene | O=C1NC(O)(CC=2C=CC=CC2)C(O)C31OC(C=CC=CCC)=C(C3=O)C | 5.61 | Fungi |  |  |  |  |  |  |  |  |
|  |  |  |  |  |  |  |  |  |  |  |  |  | Pyridovericin | O=C(C=CC(=CC(CO)CC)C)C=1C(=O)NC=C(C=2C=CC(O)=CC2)C1O | 5.56 | Fungi |  |  |  |  |  |  |  |  |
|  |  |  |  |  |  |  |  |  |  |  |  |  | 5,7-dihydroxy-2-(4-hydroxycyclohexa-1,5-dien-1-yl)-6-(1-methylpiperidin-2-yl)-4H-chromen-4-one | O=C1C=C(OC2=CC(O)=C(C(O)=C12)C3N(C)CCCC3)C=4C=CC(O)CC4 | 5.56 | COCONUT=CNP0075597, not found |  |  |  |  |  |  |  |  |
| 23 | 500.1642 | 4.01 | 5513 | [M+Na]^+^ | C_26_H_27_N_3_O_4_S |  | **—** | **—** | **—** | **—** | 7 |  | **—** | **—** | **—** | **—** |  | butyl 4,7-bis(4-ethoxyphenyl)-[1,2,5]thiadiazolo[3,4-c]pyridine-6-carboxylate | CCCCOC(=O)C1=C(C2=NSN=C2C(=N1)C3=CC=C(C=C3)OCC)C4=CC=C(C=C4)OCC | 0.93 | C_26_H_27_N_3_O_4_S | PubChem=101662282, not found |  | butyl 4,7-bis(4-ethoxyphenyl)-[1,2,5]thiadiazolo[3,4-c]pyridine-6-carboxylate |

TABLE S2 cont.

| No | Parent mass | RT | Area | Adduct | Formula |  | FBMN | | | | |  | | MSFINDER Annotation TOP 5 | | | | |  | CFM-ID 3.0 | | | | | |  | Final Annotation |
| --- | --- | --- | --- | --- | --- | --- | --- | --- | --- | --- | --- | --- | --- | --- | --- | --- | --- | --- | --- | --- | --- | --- | --- | --- | --- | --- | --- |
|  |  |  |  |  |  |  | Name | Smiles | MQ Score | Source | Network |  | Name | | Smiles | Score | Source |  | Name | | Smiles | Score | Formula | Source |  | |  |
| 24 | 431.173 | 3.22 | 4930 | [M+H]^+^ | C_23_H_26_O_8_ |  | **—** | **—** | **—** | **—** | 7 |  | **—** | | **—** | **—** | **—** |  | Diaseartemin | | COC1=C2OCOC2=CC(=C1)C1OCC2C1COC2C1=CC(OC)=C(OC)C(OC)=C1 | 0.92 | C_23_H_26_O_8_ | Archaeplastida |  | | Diaseartemin |
| 25 | 504.2135 | 4.81 | 13653 | [M+H]^+^ | C_18_H_33_NO_15_ |  | **—** | **—** | **—** | **—** | 7 |  | **—** | | **—** | **—** | **—** |  | 6-[[3-amino-4-hydroxy-6-(hydroxymethyl)-5-[3,4,5-trihydroxy-6-(hydroxymethyl)oxan-2-yl]oxyoxan-2-yl]oxymethyl]oxane-2,3,4,5-tetrol | | NC1C(O)C(OC2OC(CO)C(O)C(O)C2O)C(CO)OC1OCC1OC(O)C(O)C(O)C1O | 0.4 | C_18_H_33_NO_15_ | COCONUT=CNP0002429, not found |  | | 6-[[3-amino-4-hydroxy-6-(hydroxymethyl)-5-[3,4,5-trihydroxy-6-(hydroxymethyl)oxan-2-yl]oxyoxan-2-yl]oxymethyl]oxane-2,3,4,5-tetrol |
| 26 | 520.3404 | 4.82 | 1398 | [M+H]^+^ | C_26_H_50_NO_7_P |  | **—** | **—** | **—** | **—** | 7 |  | LysoPC(18:2(9*Z*,12*Z*)) (*) | | O=C(OCC(O)COP(=O)([O-])OCC[N+](C)(C)C)CCCCCCCC=CCC=CCCCCC (*) | 5.54 | Fungi |  | **—** | | **—** | **—** | **—** | **—** |  | | LysoPC(18:2(9*Z*,12*Z*)) |
|  |  |  |  |  |  |  |  |  |  |  |  |  | (3-hydroxy-2-octadeca-9,12-dienoyloxypropyl) 2-(trimethylazaniumyl)ethyl phosphate | | O=C(OC(CO)COP(=O)([O-])OCC[N+](C)(C)C)CCCCCCCC=CCC=CCCCCC | 5.04 | Fungi |  |  | |  |  |  |  |  | |  |

TABLE S2 cont.

| No | Parent mass | RT | Area | Adduct | Formula |  | FBMN | | | | |  | | MSFINDER Annotation TOP 5 | | | | |  | | CFM-ID 3.0 | | | | | |  | Final Annotation |
| --- | --- | --- | --- | --- | --- | --- | --- | --- | --- | --- | --- | --- | --- | --- | --- | --- | --- | --- | --- | --- | --- | --- | --- | --- | --- | --- | --- | --- |
|  |  |  |  |  |  |  | Name | Smiles | MQ Score | Source | Network |  | Name | | Smiles | Score | Source |  | | Name | | Smiles | Score | Formula | Source |  | |  |
| 27 | 506.2097 | 4.81 | 3562 | [M+H]^+^ | C_28_H_31_N_3_O_6_ |  | **—** | **—** | **—** | **—** | 7 |  | **—** | | **—** | **—** | **—** |  | | Coniel | | CC1=C(C(C(=C(N1)C)C(=O)OC2CCCN(C2)CC3=CC=CC=C3)C4=CC(=CC=C4)[N+](=O)[O-])C(=O)OC | 0.35 | C_28_H_31_N_3_O_6_ | PubChem=656668, not found |  | | Coniel |
| 28 | 476.4749 | 4.01 | 3491 | [M+H]^+^ | **—** |  | **—** | **—** | **—** | **—** | 9 |  | — | | — | — | — |  | | — | | — | — | — | — |  | | Unknown |
| 29 | 478.1828 | 4.01 | 120070 | [M+H]^+^ | C_23_H_23_N_7_O_5_ |  | ------ | ------ | ------ | ------ | 9 |  | *R*-Pralatrexate | | O=C(O)CCC(NC(=O)C1=CC=C(C=C1)C(CC#C)CC=2N=C3C(=NC2)N=C(N=C3N)N)C(=O)O | 5.19 | COCONUT=CNP0074526, not found |  | | *N*-hydroxy-*N*'-[1-(methylamino)-1-oxo-3-phenylpropan-2-yl]-3-(2-methylpropyl)-2-(thiophen-2-ylsulfanylmethyl)butanediamide | | CC(C)CC(C(CSC1=CC=CS1)C(=O)NO)C(=O)NC(CC2=CC=CC=C2)C(=O)NC | 0.94 | C_23_H_31_N_3_O_4_S_2_ | PubChem=5362422, not found |  | | *R*-Pralatrexate |
| 30 | 458.1527 | 4.01 | 1389 | [M+H-H_2_O]^+^ | — |  | — | — | — | — | 9 |  | — | | — | — | — |  | | — | | — | — | — | — |  | | Unknown |
| 31 | 456.1532 | 4.01 | 5335 | [M+Na]^+^ | C_23_H_28_ClNO_5_ |  | **—** | **—** | **—** | **—** | 9 |  | Isochromophilone VI (*) | | O=C(OC1(C(=O)C(Cl)=C2C=C(C=CC(=CC(C)CC)C)N(C=C2C1=O)CCO)C)C (*) | 5.28 | Fungi |  | | **—** | | **—** | **—** | **—** | **—** |  | | Isochromophilone VI |
|  |  |  |  |  |  |  |  |  |  |  |  |  | pentyl 4-(3-chloro-4-hydroxy-5-methoxyphenyl)-2-methyl-5-oxo-1,4,5,6,7,8-hexahydroquinoline-3-carboxylate | | O=C(OCCCCC)C1=C(NC2=C(C(=O)CCC2)C1C3=CC(Cl)=C(O)C(OC)=C3)C | 4.99 | COCONUT=CNP0119569, not found |  | |  | |  |  |  |  |  | |  |

TABLE S2 cont.

| No | Parent mass | RT | Area | Adduct | Formula |  | FBMN | | | | |  | MSFINDER Annotation TOP 5 | | | |  | CFM-ID 3.0 | | | | |  | Final Annotation |
| --- | --- | --- | --- | --- | --- | --- | --- | --- | --- | --- | --- | --- | --- | --- | --- | --- | --- | --- | --- | --- | --- | --- | --- | --- |
|  |  |  |  |  |  |  | Name | Smiles | MQ Score | Source | Network |  | Name | Smiles | Score | Source |  | Name | Smiles | Score | Formula | Source |  |  |
| 32 | 476.4079 | 4.01 | 5803 | [M+H]^+^ | — |  | — | — | — | — | 9 |  | — | — | — | — |  | — | — | — | — | — |  | Unknown |
| 33 | 462.1693 | 4.01 | 33032 | [M+H]^+^ | C_24_H_28_ClNO_6_ |  | **—** | **—** | **—** | **—** | 9 |  | 2-[7-(acetyloxy)-5-chloro-3-(3,5-dimethylhepta-1,3-dien-1-yl)-7-methyl-6,8-dioxo-2,6,7,8-tetrahydroisoquinolin-2-yl]propanoic acid (*) | O=C(OC1(C(=O)C(Cl)=C2C=C(C=CC(=CC(C)CC)C)N(C=C2C1=O)C(C(=O)O)C)C)C (*) | 4.66 | Fungi |  | **—** | **—** | **—** | **—** | **—** |  | 2-[7-(acetyloxy)-5-chloro-3-(3,5-dimethylhepta-1,3-dien-1-yl)-7-methyl-6,8-dioxo-2,6,7,8-tetrahydroisoquinolin-2-yl]propanoic acid |
| 34 | 476.1846 | 4.01 | 345240 | [M+H]^+^ | C_25_H_30_ClNO_6_ |  | **—** | **—** | **—** | **—** | 9 |  | Chaetomugilide C | O=C1OC2(C(=O)C(Cl)=C3C=C(C=CC(C)CC)N(C=C3C2=C1C(=O)C(C)C(O)C)CCO)C | 5.51 | Fungi |  | **—** | **—** | **—** | **—** | **—** |  | Penazaphilone I |
|  |  |  |  |  |  |  |  |  |  |  |  |  | Penazaphilone I (*) | O=C(OC1(C(=O)C(Cl)=C2C=C(C=CC(=CC(C)CC)C)N(C=C2C1=O)CCCC(=O)O)C)C (*) | 5.28 | Fungi |  |  |  |  |  |  |  |  |
|  |  |  |  |  |  |  |  |  |  |  |  |  | Chaetoviridide C | O=C1OC2(C(=O)C(Cl)=C3C=C(C=CC(C)CC)N(C=C3C2=C1C(=O)CC)CCOCCO)C | 5.2 | Fungi |  |  |  |  |  |  |  |  |
|  |  |  |  |  |  |  |  |  |  |  |  |  | 4-[(2-{6-chloro-4-methyl-7-[(3-methylbut-2-en-1-yl)oxy]-2-oxo-2*H*-chromen-3-yl}acetamido)methyl]cyclohexane-1-carboxylic acid | O=C1OC=2C=C(OCC=C(C)C)C(Cl)=CC2C(=C1CC(=O)NCC3CCC(C(=O)O)CC3)C | 4.95 | COCONUT=CNP0391570, not found |  |  |  |  |  |  |  |  |

TABLE S2 cont.

| No | Parent mass | RT | Area | Adduct | Formula |  | FBMN | | | | |  | MSFINDER Annotation TOP 5 | | | |  | CFM-ID 3.0 | | | | |  | Final Annotation |
| --- | --- | --- | --- | --- | --- | --- | --- | --- | --- | --- | --- | --- | --- | --- | --- | --- | --- | --- | --- | --- | --- | --- | --- | --- |
|  |  |  |  |  |  |  | Name | Smiles | MQ Score | Source | Network |  | Name | Smiles | Score | Source |  | Name | Smiles | Score | Formula | Source |  |  |
| 35 | 490.2053 | 4.01 | 3798 | [M+NH_4_]^+^ | C_25_H_28_O_9_ |  | **—** | **—** | **—** | **—** | 9 |  | Aspernolide I | O=C1OC(C(=O)OC)(C(C=2C=CC(O)=CC2)=C1OC)CC3=CC=C(O)C(=C3)CC(O)C(O)(C)C | 3.86 | Fungi |  | **—** | **—** | **—** | **—** | **—** |  | Aspernolide I |
|  |  |  |  |  |  |  |  |  |  |  |  |  | Aspernolide J | O=C1OC(C(=O)OC)(C(C=2C=CC(O)=C(O)C2)=C1OC)CC3=CC=C(O)C(=C3)CCC(O)(C)C | 3.86 | Fungi |  |  |  |  |  |  |  |  |
|  |  |  |  |  |  |  |  |  |  |  |  |  | Naphthopyranomycin | O=C1C2=CC=3C=C(OC(C3C(O)=C2C(=O)C(=C1CC(=O)C)C4OCC(O)C(O4)CO)CCC)C | 3.85 | Bacterium |  |  |  |  |  |  |  |  |
|  |  |  |  |  |  |  |  |  |  |  |  |  | 5-hydroxy-2,2-dimethyl-8-{[(2-methylbut-2-enoyl)oxy]methyl}-6-oxo-2*H*,3*H*,4*H*,6*H*-pyrano[3,2-g]chromen-3-yl 2,3-dimethyloxirane-2-carboxylate | O=C(OCC=1OC=2C=C3OC(C)(C)C(OC(=O)C4(OC4C)C)CC3=C(O)C2C(=O)C1)C(=CC)C | 3.84 | Archaeplastida |  |  |  |  |  |  |  |  |

TABLE S2 cont.

| No | Parent mass | RT | Area | Adduct | Formula |  | FBMN | | | | |  | MSFINDER Annotation TOP 5 | | | |  | CFM-ID 3.0 | | | | |  | Final Annotation |
| --- | --- | --- | --- | --- | --- | --- | --- | --- | --- | --- | --- | --- | --- | --- | --- | --- | --- | --- | --- | --- | --- | --- | --- | --- |
|  |  |  |  |  |  |  | Name | Smiles | MQ Score | Source | Network |  | Name | Smiles | Score | Source |  | Name | Smiles | Score | Formula | Source |  |  |
| 36 | 474.2129 | 4.01 | 29880 | [M+Na]^+^ | C_23_H_33_NO_8_ |  | **—** | **—** | **—** | **—** | 9 |  | 1-{[(cyclohexyloxy)carbonyl]oxy}ethyl 1-(1-hydroxyethyl)-5-methoxy-2-oxo-1*H*,2*H*,5*H*,6*H*,7*H*,8*H*,8a*H*,8b*H*-azeto[2,1-a]isoindole-4-carboxylate | O=C(OC(OC(=O)C1=C2C(OC)CCCC2C3N1C(=O)C3C(O)C)C)OC4CCCCC4 | 5.06 | COCONUT=CNP0074453, not found |  | Valery 4-Hydroxy Valsartan | CC(C)C(C(=O)O)N(CC1=CC=C(C=C1)C2=CC=CC=C2C3=NNN=N3)C(=O)CCC(C)O | 0.87 | C_24_H_29_N_5_O_4_ | PubChem=9911647, not found |  | 1-{[(cyclohexyloxy)carbonyl]oxy}ethyl 1-(1-hydroxyethyl)-5-methoxy-2-oxo-1*H*,2*H*,5*H*,6*H*,7*H*,8*H*,8a*H*,8b*H*-azeto[2,1-a]isoindole-4-carboxylate |
|  |  |  |  |  |  |  |  |  |  |  |  |  | 4,12-dihydroxy-8-(4-methoxyphenyl)-2,9,14-trioxa-6-azatricyclo[9.2.1.0³,⁷]tetradecan-13-yl 4-methylpentanoate | O=C(OC1C2OC(COC(C3=CC=C(OC)C=C3)C4NCC(O)C4O2)C1O)CCC(C)C | 4.96 | COCONUT=CNP0331290, not found |  |  |  |  |  |  |  |  |
|  |  |  |  |  |  |  |  |  |  |  |  |  | [3,5,14-trihydroxy-13-methyl-17-(5-oxo-2*H*-furan-3-yl)-2,3,4,6,7,8,9,11,12,15,16,17-dodecahydro-1*H*-cyclopenta[a]phenanthren-10-yl]methyl nitrate (*) | O=C1OCC(=C1)C2CCC3(O)C4CCC5(O)CC(O)CCC5(CO[N+](=O)[O-])C4CCC23C (*) | 4.66 | COCONUT=CNP0362691, not found |  |  |  |  |  |  |  |  |
| 37 | 464.1688 | 4.01 | 10591 | [M+K]^+^ | C_26_H_29_NO_9_ |  | **—** | **—** | **—** | **—** | 9 |  | Malfilamentoside B (*) | O=C1OC(OC2OC(COC)C(O)C(O)C2NC(=O)C)C(C=3C=CC=CC3)=C1C(O)C=4C=CC=CC4 (*) | 5.46 | Fungi |  | **—** | **—** | **—** | **—** | **—** |  | Malfilamentoside B |
|  |  |  |  |  |  |  |  |  |  |  |  |  | 13-Deoxycarminomycin | O=C1C=2C=CC=C(O)C2C(=O)C=3C(O)=C4C(=C(O)C13)CC(O)(CC)CC4OC5OC(C)C(O)C(N)C5 | 5.43 | Bacterium |  |  |  |  |  |  |  |  |

TABLE S2 cont.

| No | Parent mass | RT | Area | Adduct | Formula |  | FBMN | | | | |  | MSFINDER Annotation TOP 5 | | | |  | CFM-ID 3.0 | | | | |  | Final Annotation |
| --- | --- | --- | --- | --- | --- | --- | --- | --- | --- | --- | --- | --- | --- | --- | --- | --- | --- | --- | --- | --- | --- | --- | --- | --- |
|  |  |  |  |  |  |  | Name | Smiles | MQ Score | Source | Network |  | Name | Smiles | Score | Source |  | Name | Smiles | Score | Formula | Source |  |  |
| 38 | 472.1297 | 4.01 | 2198 | [M+K]^+^ | C_23_H_28_ClNO_5_ |  | **—** | **—** | **—** | **—** | 9 |  | Isochromophilone VI (*) | O=C(OC1(C(=O)C(Cl)=C2C=C(C=CC(=CC(C)CC)C)N(C=C2C1=O)CCO)C)C (*) | 5.37 | Fungi |  | **—** | **—** | **—** | **—** | **—** |  | Isochromophilone VI |
|  |  |  |  |  |  |  |  |  |  |  |  |  | pentyl 4-(3-chloro-4-hydroxy-5-methoxyphenyl)-2-methyl-5-oxo-1,4,5,6,7,8-hexahydroquinoline-3-carboxylate | O=C(OCCCCC)C1=C(NC2=C(C(=O)CCC2)C1C3=CC(Cl)=C(O)C(OC)=C3)C | 5.14 | COCONUT=CNP0119569, not found |  |  |  |  |  |  |  |  |
| 39 | 434.1739 | 4.01 | 50927 | [M+H]^+^ | C_23_H_28_ClNO_5_ |  | **—** | **—** | **—** | **—** | 12 |  | Isochromophilone VI (*) | O=C(OC1(C(=O)C(Cl)=C2C=C(C=CC(=CC(C)CC)C)N(C=C2C1=O)CCO)C)C (*) | 4.68 | Fungi |  | **—** | **—** | **—** | **—** | **—** |  | Isochromophilone VI |
|  |  |  |  |  |  |  |  |  |  |  |  |  | pentyl 4-(3-chloro-4-hydroxy-5-methoxyphenyl)-2-methyl-5-oxo-1,4,5,6,7,8-hexahydroquinoline-3-carboxylate | O=C(OCCCCC)C1=C(NC2=C(C(=O)CCC2)C1C3=CC(Cl)=C(O)C(OC)=C3)C | 4.55 | COCONUT=CNP0119569, not found |  |  |  |  |  |  |  |  |
| 40 | 436.1717 | 4.01 | 17258 | [M+H]^+^ | C_24_H_25_N_3_O_3_S |  | **—** | **—** | **—** | **—** | 12 |  | 4-benzyl-*N*-{[1-(7-hydroxy-2-oxo-2*H*-chromen-8-yl)ethylidene]amino}piperidine-1-carboimidothioic acid | O=C1OC2=C(C=C1)C=CC(O)=C2C(=NN=C(S)N3CCC(CC=4C=CC=CC4)CC3)C | 4.6 | COCONUT=CNP0163612, not found |  | 2-[6-cyano-3-[(2,2-difluoro-2-pyridin-2-ylethyl)amino]-2-fluorophenyl]-*N*-[2-(diaminomethylideneamino)oxyethyl]acetamide | C1=CC=NC(=C1)C(CNC2=C(C(=C(C=C2)C#N)CC(=O)NCCON=C(N)N)F)(F)F | 0.67 | C_19_H_20_F_3_N_7_O_2_ | PubChem=9867609, not found |  | 4-benzyl-*N*-{[1-(7-hydroxy-2-oxo-2*H*-chromen-8-yl)ethylidene]amino}piperidine-1-carboimidothioic acid |

TABLE S2 cont.

| Parent mass | RT | Area | Adduct | Formula |  | FBMN | | | | |  | MSFINDER Annotation TOP 5 | | | |  | CFM-ID 3.0 | | | | |  | Final Annotation |
| --- | --- | --- | --- | --- | --- | --- | --- | --- | --- | --- | --- | --- | --- | --- | --- | --- | --- | --- | --- | --- | --- | --- | --- |
|  |  |  |  |  |  | Name | Smiles | MQ Score | Source | Network |  | Name | Smiles | Score | Source |  | Name | Smiles | Score | Formula | Source |  |  |
|  |  |  |  |  |  |  |  |  |  |  |  | ethyl 6-amino-5-(1,3-benzothiazol-2-yl)-4-[4-(dimethylamino)phenyl]-2-methyl-4*H*-pyran-3-carboxylate | O=C(OCC)C1=C(OC(N)=C(C2=NC=3C=CC=CC3S2)C1C4=CC=C(C=C4)N(C)C)C | 4.53 | COCONUT=CNP0049001, not found |  |  |  |  |  |  |  |  |
|  |  |  |  |  |  |  |  |  |  |  |  | 1-(6-methoxy-2,2,4-trimethylquinolin-1-yl)-2-[[5-(4-methylphenyl)-1,3,4-oxadiazol-2-yl]sulfanyl]ethanone | O=C(N1C=2C=CC(OC)=CC2C(=CC1(C)C)C)CSC3=NN=C(O3)C=4C=CC(=CC4)C | 4.38 | ChEBI=CHEBI:116550, not found |  |  |  |  |  |  |  |  |
|  |  |  |  |  |  |  |  |  |  |  |  | 3-(1,3-benzothiazol-2-yl)-6-ethyl-7-hydroxy-8-[(4-methylpiperazin-1-yl)methyl]chromen-4-one | O=C1C(=COC2=C1C=C(C(O)=C2CN3CCN(C)CC3)CC)C4=NC=5C=CC=CC5S4 | 4.38 | COCONUT=CNP0444908, not found |  |  |  |  |  |  |  |  |
|  |  |  |  |  |  |  |  |  |  |  |  | 3'-[2-(methylsulfanyl)ethyl]-5'-(2-phenylethyl)-1,2,3',3'a,4',5',6',6'a-octahydro-2'*H*-spiro[indole-3,1'-pyrrolo[3,4-c]pyrrole]-2,4',6'-trione | O=C1NC=2C=CC=CC2C31NC(CCSC)C4C(=O)N(C(=O)C43)CCC=5C=CC=CC5 | 4.3 | COCONUT=CNP0141843, not found |  |  |  |  |  |  |  |  |

TABLE S2 cont.

| No | Parent mass | RT | Area | Adduct | Formula |  | FBMN | | | | |  | MSFINDER Annotation TOP 5 | | | |  | CFM-ID 3.0 | | | | |  | Final Annotation |
| --- | --- | --- | --- | --- | --- | --- | --- | --- | --- | --- | --- | --- | --- | --- | --- | --- | --- | --- | --- | --- | --- | --- | --- | --- |
|  |  |  |  |  |  |  | Name | Smiles | MQ Score | Source | Network |  | Name | Smiles | Score | Source |  | Name | Smiles | Score | Formula | Source |  |  |
| 41 | 417.181 | 4.01 | 3187 | [M+Na]^+^ | C_23_H_26_N_2_O_4_ |  | **—** | **—** | **—** | **—** | 12 |  | 19*S*-Acetoxytabersonine | O=C(OC)C1=C2NC=3C=CC=CC3C24CCN5CC=CC(C1)(C(OC(=O)C)C)C54 | 5.31 | Plantae |  | 2,2,5,7-tetramethyl-6-(2-{[3,4,5-trihydroxy-6-(hydroxymethyl)oxan-2-yl]oxy}ethyl)-2,3-dihydro-1*H*-inden-1-one | CC1=CC2=C(C(=O)C(C)(C)C2)C(C)=C1CCOC1OC(CO)C(O)C(O)C1O | 0.66 | C_21_H_30_O_7_ | COCONUT=CNP0229645, not found |  | 19*S*-Acetoxytabersonine |
|  |  |  |  |  |  |  |  |  |  |  |  |  | Kopsijasmine | O=C(OC)C1=CC23CCCN4CCC5(C=6C=CC=CC6N(C(=O)OC)C15CC2)C43 | 5.23 | Plantae |  |  |  |  |  |  |  |  |
|  |  |  |  |  |  |  |  |  |  |  |  |  | ethyl 5-hydroxy-4-[(4-methylpiperazin-1-yl)methyl]-2-phenyl-1-benzofuran-3-carboxylate | O=C(OCC)C1=C(OC2=CC=C(O)C(=C21)CN3CCN(C)CC3)C=4C=CC=CC4 | 5.17 | COCONUT=CNP0435951, not found |  |  |  |  |  |  |  |  |
|  |  |  |  |  |  |  |  |  |  |  |  |  | Brucin | O=C1N2C3=CC(OC)=C(OC)C=C3C45CCN6CC7=CCOC(C1)C(C7CC64)C25 | 5.12 | Plantae |  |  |  |  |  |  |  |  |
|  |  |  |  |  |  |  |  |  |  |  |  |  | Akuammiline | O=C(OCC1(C(=O)OC)C2C(=CC)CN3CCC41C=5C=CC=CC5N=C4C3C2)C | 5.11 | Plantae |  |  |  |  |  |  |  |  |

TABLE S2 cont.

| No | Parent mass | RT | Area | Adduct | Formula |  | FBMN | | | | |  | MSFINDER Annotation TOP 5 | | | |  | CFM-ID 3.0 | | | | |  | Final Annotation |
| --- | --- | --- | --- | --- | --- | --- | --- | --- | --- | --- | --- | --- | --- | --- | --- | --- | --- | --- | --- | --- | --- | --- | --- | --- |
|  |  |  |  |  |  |  | Name | Smiles | MQ Score | Source | Network |  | Name | Smiles | Score | Source |  | Name | Smiles | Score | Formula | Source |  |  |
| 42 | 433.1639 | 4.01 | 4615 | [M+Na]^+^ | C_24_H_26_O_6_ |  | **—** | **—** | **—** | **—** | 12 |  | Cochinchinone C | O=C1C=2C(O)=CC=CC2OC34C1=CC5(OC)C(=O)C4(OC(C)(C)C3C5)CC=C(C)C | 4.05 | Plantae |  | **—** | **—** | **—** | **—** | **—** |  | Multformin A |
|  |  |  |  |  |  |  |  |  |  |  |  |  | 1-Isomangostin | O=C1C=2C(OC3=CC(O)=C(OC)C(=C13)CC=C(C)C)=CC(O)=C4C2OC(C)(C)CC4 | 4.02 | Plantae |  |  |  |  |  |  |  |  |
|  |  |  |  |  |  |  |  |  |  |  |  |  | Multformin A | O=C1OC2(C(=O)C=C3C=C(OC=C3C2C1C(=O)C(C)CC)C4C(=O)C=CCC4C)C | 4.01 | Fungi |  |  |  |  |  |  |  |  |
|  |  |  |  |  |  |  |  |  |  |  |  |  | Aspercyclide A | O=CC=1C(O)=CC=C2OC3=CC=C(C(=C3)C(=O)OC(CCCCC)C(O)C=CC21)C | 3.99 | Fungi |  |  |  |  |  |  |  |  |
|  |  |  |  |  |  |  |  |  |  |  |  |  | Paxanthonin | O=C1C2=CC=C(O)C(OC)=C2OC3=CC(O)=C(C(O)=C13)C4CC(C(=C)C)CC4(C)C | 3.97 | Plantae |  |  |  |  |  |  |  |  |

TABLE S2 cont.

| No | Parent mass | RT | Area | Adduct | Formula |  | FBMN | | | | |  | MSFINDER Annotation TOP 5 | | | |  | CFM-ID 3.0 | | | | |  | Final Annotation |
| --- | --- | --- | --- | --- | --- | --- | --- | --- | --- | --- | --- | --- | --- | --- | --- | --- | --- | --- | --- | --- | --- | --- | --- | --- |
|  |  |  |  |  |  |  | Name | Smiles | MQ Score | Source | Network |  | Name | Smiles | Score | Source |  | Name | Smiles | Score | Formula | Source |  |  |
| 43 | 414.1937 | 4.01 | 2782 | [M+H]^+^ | C_23_H_27_NO_6_ |  | **—** | **—** | **—** | **—** | 12 |  | 3-Methyl-Antibiotic G-15F | O=C1C2=CC=C(C(O)=C2C(=O)C3=C1C=C(OC3C)C)C4OC(C)C(O)C(N(C)C)C4 | 5.59 | Plantae |  | 8-[(2-acetamido-3-phenylbutanoyl)oxy]-8-(2-methyloxiran-2-yl)octa-2,4,6-trienoic acid | CC(C1=CC=CC=C1)C(C(=O)OC(C=CC=CC=CC(=O)O)C2(CO2)C)NC(=O)C | 0.99 | C_23_H_27_NO_6_ | Fungi |  | 8-[(2-acetamido-3-phenylbutanoyl)oxy]-8-(2-methyloxiran-2-yl)octa-2,4,6-trienoic acid |
|  |  |  |  |  |  |  |  |  |  |  |  |  | 4-{[2-({6-methyl-4-oxo-1*H*,2*H*,3*H*,4*H*-cyclopenta[c]chromen-7-yl}oxy)acetamido]methyl}cyclohexane-1-carboxylic acid | O=C1OC2=C(C=CC(OCC(=O)NCC3CCC(C(=O)O)CC3)=C2C)C4=C1CCC4 | 5.48 | COCONUT=CNP0206603, not found |  |  |  |  |  |  |  |  |
|  |  |  |  |  |  |  |  |  |  |  |  |  | 4-{[2-({7-methyl-4-oxo-1*H*,2*H*,3*H*,4*H*-cyclopenta[c]chromen-9-yl}oxy)acetamido]methyl}cyclohexane-1-carboxylic acid | O=C1OC2=CC(=CC(OCC(=O)NCC3CCC(C(=O)O)CC3)=C2C4=C1CCC4)C | 5.46 | COCONUT=CNP0123573, not found |  |  |  |  |  |  |  |  |
|  |  |  |  |  |  |  |  |  |  |  |  |  | 4-({[1-hydroxy-2-({4-oxo-1*H*,2*H*,3*H*,4*H*-cyclopenta[c]chromen-7-yl}oxy)propylidene]amino}methyl)cyclohexane-1-carboxylic acid | O=C1OC=2C=C(OC(C(=O)NCC3CCC(C(=O)O)CC3)C)C=CC2C4=C1CCC4 | 5.44 | COCONUT=CNP0340594, not found |  |  |  |  |  |  |  |  |
|  |  |  |  |  |  |  |  |  |  |  |  |  | AK toxin I | O=C(O)C=CC=CC=CC(OC(=O)C(NC(=O)C)C(C=1C=CC=CC1)C)C2(OC2)C | 5.44 | Fungi |  |  |  |  |  |  |  |  |

TABLE S2 cont.

| No | Parent mass | RT | Area | Adduct | Formula |  | FBMN | | | | |  | | MSFINDER Annotation TOP 5 | | | | |  | | CFM-ID 3.0 | | | | | |  | Final Annotation |
| --- | --- | --- | --- | --- | --- | --- | --- | --- | --- | --- | --- | --- | --- | --- | --- | --- | --- | --- | --- | --- | --- | --- | --- | --- | --- | --- | --- | --- |
|  |  |  |  |  |  |  | Name | Smiles | MQ Score | Source | Network |  | Name | | Smiles | Score | Source |  | | Name | | Smiles | Score | Formula | Source |  | |  |
| 44 | 432.2019 | 4.02 | 3855 | [M+NH_4_]^+^ | C_23_H_26_O_7_ |  | **—** | **—** | **—** | **—** | 12 |  | Vermixocin B (*) | | O=C1OCC2=CC(=CC(O)=C2OC3=CC=C(C(OC)=C13)C(OC(=O)C)CC(C)C)C (*) | 5.49 | Fungi |  | | **—** | | **—** | **—** | **—** | **—** |  | | Vermixocin B |
|  |  |  |  |  |  |  |  |  |  |  |  |  | Mammea E/Bc Cyclo D | | O=C1OC2=C(C(=O)CCC)C(O)=C3C=CC(OC3=C2C(=C1)C(OC(=O)C)CC)(C)C | 5.40 | Plantae |  | |  | |  |  |  |  |  | |  |
|  |  |  |  |  |  |  |  |  |  |  |  |  | Purpactin B | | O=C(OC(C1=CC=C2OC3(C(=O)C=C(C=C3CO)C)C(=O)C2=C1OC)CC(C)C)C | 5.40 | Fungi |  | |  | |  |  |  |  |  | |  |
|  |  |  |  |  |  |  |  |  |  |  |  |  | Mammea E/Ac Cyclo D | | O=C1OC=2C=3C=CC(OC3C(C(=O)CCC)=C(O)C2C(=C1)C(OC(=O)C)CC)(C)C | 5.39 | Plantae |  | |  | |  |  |  |  |  | |  |
|  |  |  |  |  |  |  |  |  |  |  |  |  | Chaetoglobosin L | | O=C(O)C1C(=O)C(C)CC(=C(C=CC=2OC(=C3OC1(C(O)=C3O)C)C(=CC2)C)C)C | 5.38 | COCONUT=CNP0276527, not found |  | |  | |  |  |  |  |  | |  |

TABLE S2 cont.

| No | Parent mass | RT | Area | Adduct | Formula |  | FBMN | | | | |  | MSFINDER Annotation TOP 5 | | | |  | CFM-ID 3.0 | | | | |  | Final Annotation |
| --- | --- | --- | --- | --- | --- | --- | --- | --- | --- | --- | --- | --- | --- | --- | --- | --- | --- | --- | --- | --- | --- | --- | --- | --- |
|  |  |  |  |  |  |  | Name | Smiles | MQ Score | Source | Network |  | Name | Smiles | Score | Source |  | Name | Smiles | Score | Formula | Source |  |  |
| 45 | 420.158 | 4.01 | 1119 | [M+H]^+^ | C_22_H_26_ClNO_5_ |  | **—** | **—** | **—** | **—** | 12 |  | 7-[2-(4a-hydroxy-decahydroisoquinolin-2-yl)-2-oxoethoxy]-6-chloro-3,4-dimethyl-2*H*-chromen-2-one | O=C1OC=2C=C(OCC(=O)N3CCC4(O)CCCCC4C3)C(Cl)=CC2C(=C1C)C | 4.78 | COCONUT=CNP0200985, not found |  | 4-[[(3*S*)-1-azabicyclo[2.2.2]octan-3-yl]amino]-3-(1*H*-benzimidazol-2-yl)-6-chloro-1*H*-quinolin-2-one | ClC1=CC2=C(NC(=O)C(C3=NC4=CC=CC=C4N3)=C2N[C@@H]2CN3CCC2CC3)C=C1 | 0.95 | C_23_H_22_ClN_5_O | Drug Bank=DB06852, not found |  | 7-[2-(4a-hydroxy-decahydroisoquinolin-2-yl)-2-oxoethoxy]-6-chloro-3,4-dimethyl-2*H*-chromen-2-one |
|  |  |  |  |  |  |  |  |  |  |  |  |  | 7-[2-(4a-hydroxy-decahydroisoquinolin-2-yl)-2-oxoethoxy]-6-chloro-4-ethyl-2*H*-chromen-2-one | O=C1OC=2C=C(OCC(=O)N3CCC4(O)CCCCC4C3)C(Cl)=CC2C(=C1)CC | 4.78 | COCONUT=CNP0291953, not found |  |  |  |  |  |  |  |  |
|  |  |  |  |  |  |  |  |  |  |  |  |  | 2-chloro-1-{1-[(3,4-dimethoxyphenyl)methyl]-6,7-dimethoxy-1,2,3,4-tetrahydroisoquinolin-2-yl}ethan-1-one | O=C(N1CCC2=CC(OC)=C(OC)C=C2C1CC3=CC=C(OC)C(OC)=C3)CCl | 4.35 | COCONUT=CNP0377175, not found |  |  |  |  |  |  |  |  |
| 46 | 374.1513 | 4.06 | 5311 | [M+Na]^+^ | C_18_H_25_NO_6_ |  | **—** | **—** | **—** | **—** | 13 |  | Integerrimine N-oxide | O=C1OC2CCN3(=O)CC=C(COC(=O)C(O)(C)C(C)CC1=CC)C23 | 4.92 | Plantae |  | 2-(hydroxymethyl)-5-[6-[(4-hydroxy-3-methylbut-2-enyl)amino]purin-9-yl]oxolane-3,4-diol | CC(=CCNC1=C2C(=NC=N1)N(C=N2)C3C(C(C(O3)CO)O)O)CO | 0.99 | C_15_H_21_N_5_O_5_ | Archaeplastida |  | Retrorsine |
|  |  |  |  |  |  |  |  |  |  |  |  |  | Retrorsine (*) | O=C1OC2CCN3CC=C(COC(=O)C(O)(CO)C(C)CC1=CC)C32 (*) | 4.86 | Plantae |  |  |  |  |  |  |  |  |

TABLE S2 cont.

| No | Parent mass | RT | Area | Adduct | Formula |  | FBMN | | | | |  | | MSFINDER Annotation TOP 5 | | | | |  | | CFM-ID 3.0 | | | | | |  | Final Annotation |
| --- | --- | --- | --- | --- | --- | --- | --- | --- | --- | --- | --- | --- | --- | --- | --- | --- | --- | --- | --- | --- | --- | --- | --- | --- | --- | --- | --- | --- |
|  |  |  |  |  |  |  | Name | Smiles | MQ Score | Source | Network |  | Name | | Smiles | Score | Source |  | | Name | | Smiles | Score | Formula | Source |  | |  |
|  |  |  |  |  |  |  |  |  |  |  |  |  | Jacobine | | O=C1OCC2=CCN3CCC(OC(=O)C4(OC4C)CC(C)C1(O)C)C23 | 4.82 | Plantae |  | |  | |  |  |  |  |  | |  |
|  |  |  |  |  |  |  |  |  |  |  |  |  | Anacrotine | | O=C1OC2C(O)CN3CC=C(COC(=O)C(O)(C)C(C)CC1=CC)C32 | 4.76 | Plantae |  | |  | |  |  |  |  |  | |  |
|  |  |  |  |  |  |  |  |  |  |  |  |  | Angularine | | O=C1OC2CCN3CC(O)C(COC(=O)C(O)(C(=C)CC1=CC)C)C32 | 4.75 | Plantae |  | |  | |  |  |  |  |  | |  |
| 47 | 462.1649 | 4.06 | 8174 | [M+H]^+^ | C_24_H_28_ClNO_6_ |  | **—** | **—** | **—** | **—** | 13 |  | 2-[7-(acetyloxy)-5-chloro-3-(3,5-dimethylhepta-1,3-dien-1-yl)-7-methyl-6,8-dioxo-2,6,7,8-tetrahydroisoquinolin-2-yl]propanoic acid (*) | | O=C(OC1(C(=O)C(Cl)=C2C=C(C=CC(=CC(C)CC)C)N(C=C2C1=O)C(C(=O)O)C)C)C (*) | 3.58 | Fungi |  | | **—** | | **—** | **—** | **—** | **—** |  | | 2-[7-(acetyloxy)-5-chloro-3-(3,5-dimethylhepta-1,3-dien-1-yl)-7-methyl-6,8-dioxo-2,6,7,8-tetrahydroisoquinolin-2-yl]propanoic acid |
| 48 | 478.1828 | 4.06 | 26434 | [M+H]^+^ | C_23_H_23_N_7_O_5_ |  | **—** | **—** | **—** | **—** | 13 |  | Pralatrexate (*) | | O=C(O)CCC(NC(=O)C1=CC=C(C=C1)C(CC#C)CC=2N=C3C(=NC2)N=C(N=C3N)N)C(=O)O (*) | 5.19 | COCONUT=CNP0074526, not found |  | | *N*-hydroxy-*N*'-[1-(methylamino)-1-oxo-3-phenylpropan-2-yl]-3-(2-methylpropyl)-2-(thiophen-2-ylsulfanylmethyl)butanediamide | | CC(C)CC(C(CSC1=CC=CS1)C(=O)NO)C(=O)NC(CC2=CC=CC=C2)C(=O)NC | 0.94 | C_23_H_31_N_3_O_4_S_2_ | PubChem=2299, not found |  | | Pralatrexate |

TABLE S2 cont.

| No | Parent mass | RT | Area | Adduct | Formula |  | FBMN | | | | |  | MSFINDER Annotation TOP 5 | | | |  | CFM-ID 3.0 | | | | |  | Final Annotation |
| --- | --- | --- | --- | --- | --- | --- | --- | --- | --- | --- | --- | --- | --- | --- | --- | --- | --- | --- | --- | --- | --- | --- | --- | --- |
|  |  |  |  |  |  |  | Name | Smiles | MQ Score | Source | Network |  | Name | Smiles | Score | Source |  | Name | Smiles | Score | Formula | Source |  |  |
| 49 | 405.0572 | 4.06 | 1856 | [M+K]^+^ | C_17_H_18_O_9_ |  | **—** | **—** | **—** | **—** | 13 |  | Furocoumarinic acid glucoside | O=C(O)C=CC1=CC=2C=COC2C=C1OC3OC(CO)C(O)C(O)C3O | 4.99 | Plantae |  | Mepron | C1CC(CCC1C2=CC=C(C=C2)Cl)C3=C(C4=CC=CC=C4C(=O)C3=O)O | 1 | C_22_H_19_ClO_3_ | COCONUT=CNP0281811, not found |  | Furocoumarinic acid glucoside |
|  |  |  |  |  |  |  |  |  |  |  |  |  | Isopsoralenoside | O=C(O)C=CC1=CC=C2OC=CC2=C1OC3OC(CO)C(O)C(O)C3O | 4.89 | Plantae |  |  |  |  |  |  |  |  |
|  |  |  |  |  |  |  |  |  |  |  |  |  | Rubinaphthin A (*) | O=C(O)C=1C=C(OC2OC(CO)C(O)C(O)C2O)C=3C=CC=CC3C1O (*) | 4.78 | Plantae |  |  |  |  |  |  |  |  |
|  |  |  |  |  |  |  |  |  |  |  |  |  | {3,4,5-trihydroxy-6-[(2-oxo-2*H*-chromen-7-yl)oxy]oxan-2-yl}methyl acetate | O=C1OC=2C=C(OC3OC(COC(=O)C)C(O)C(O)C3O)C=CC2C=C1 | 4.69 | COCONUT=CNP0302443, not found |  |  |  |  |  |  |  |  |
|  |  |  |  |  |  |  |  |  |  |  |  |  | 9-{[3,4,5-trihydroxy-6-(hydroxymethyl)oxan-2-yl]oxy}-2*H*,3*H*,7*H*-furo[3,2-g]chromen-7-one | O=C1OC=2C(OC3OC(CO)C(O)C(O)C3O)=C4OCCC4=CC2C=C1 | 4.69 | COCONUT=CNP0306643, not found |  |  |  |  |  |  |  |  |

TABLE S2 cont.

| No | Parent mass | RT | Area | Adduct | Formula |  | FBMN | | | | |  | MSFINDER Annotation TOP 5 | | | |  | CFM-ID 3.0 | | | | |  | Final Annotation |
| --- | --- | --- | --- | --- | --- | --- | --- | --- | --- | --- | --- | --- | --- | --- | --- | --- | --- | --- | --- | --- | --- | --- | --- | --- |
|  |  |  |  |  |  |  | Name | Smiles | MQ Score | Source | Network |  | Name | Smiles | Score | Source |  | Name | Smiles | Score | Formula | Source |  |  |
| 50 | 476.1846 | 4.06 | 74560 | [M+H]^+^ | C_25_H_30_ClNO_6_ |  | **—** | **—** | **—** | **—** | 13 |  | Chaetomugilide C | O=C1OC2(C(=O)C(Cl)=C3C=C(C=CC(C)CC)N(C=C3C2=C1C(=O)C(C)C(O)C)CCO)C | 4.59 | Fungi |  | **—** | **—** | **—** | **—** | **—** |  | Penazaphilone I |
|  |  |  |  |  |  |  |  |  |  |  |  |  | Penazaphilone I (*) | O=C(OC1(C(=O)C(Cl)=C2C=C(C=CC(=CC(C)CC)C)N(C=C2C1=O)CCCC(=O)O)C)C (*) | 4.47 | Fungi |  |  |  |  |  |  |  |  |
|  |  |  |  |  |  |  |  |  |  |  |  |  | 4-[(2-{6-chloro-4-methyl-7-[(3-methylbut-2-en-1-yl)oxy]-2-oxo-2*H*-chromen-3-yl}acetamido)methyl]cyclohexane-1-carboxylic acid | O=C1OC=2C=C(OCC=C(C)C)C(Cl)=CC2C(=C1CC(=O)NCC3CCC(C(=O)O)CC3)C | 4.46 | COCONUT=CNP0391570, not found |  |  |  |  |  |  |  |  |
|  |  |  |  |  |  |  |  |  |  |  |  |  | Chaetoviridide C | O=C1OC2(C(=O)C(Cl)=C3C=C(C=CC(C)CC)N(C=C3C2=C1C(=O)CC)CCOCCO)C | 4.44 | Fungi |  |  |  |  |  |  |  |  |

TABLE S2 cont.

| No | Parent mass | RT | Area | Adduct | Formula |  | FBMN | | | | |  | MSFINDER Annotation TOP 5 | | | |  | CFM-ID 3.0 | | | | |  | Final Annotation |
| --- | --- | --- | --- | --- | --- | --- | --- | --- | --- | --- | --- | --- | --- | --- | --- | --- | --- | --- | --- | --- | --- | --- | --- | --- |
|  |  |  |  |  |  |  | Name | Smiles | MQ Score | Source | Network |  | Name | Smiles | Score | Source |  | Name | Smiles | Score | Formula | Source |  |  |
| 51 | 346.1562 | 4.06 | 7679 | [M+NH_4_]^+^ | C_15_H_20_O_8_ |  | **—** | **—** | **—** | **—** | 13 |  | Anisatin | O=C1OC2CC3(C1O)C(C)CC(O)C3(O)C4(C(=O)OC4)C2(O)C | 4.85 | Plantae |  | 6-[(*E*)-2-(3,4-dihydroxyphenyl)ethenyl]-3-[(1*E*)-3-methylbut-1-en-1-yl]benzene-1,2,4-triol | [H]/C(C1=CC=C(O)C(O)=C1)=C([H])\C2=CC(O)=C(/C([H])=C(C(C)C)\[H])C(O)=C2O | 0.96 | C_19_H_20_O_5_ | HMDB=HMDB0129032, not found |  | Anisatin |
|  |  |  |  |  |  |  |  |  |  |  |  |  | Paeonoside | O=C(C1=CC=C(OC)C=C1OC2OC(CO)C(O)C(O)C2O)C | 4.64 | Plantae |  |  |  |  |  |  |  |  |
|  |  |  |  |  |  |  |  |  |  |  |  |  | 2-(1-Propen-1-Yl)-4-Hydroxymethyl-3-Furanylcarbonyla-L-Rhamnopyranoside | O=C(OC1OC(C)C(O)C(O)C1O)C2=C(OC=C2CO)C=CC | 4.62 | Bacterium |  |  |  |  |  |  |  |  |
|  |  |  |  |  |  |  |  |  |  |  |  |  | Majucin | O=C1OC2CC3(C1O)C(C)CC(O)C3(O)C4(C)COC(=O)C24O | 4.54 | Plantae |  |  |  |  |  |  |  |  |
|  |  |  |  |  |  |  |  |  |  |  |  |  | Androsin | O=C(C1=CC=C(OC2OC(CO)C(O)C(O)C2O)C(OC)=C1)C | 4.53 | Plantae |  |  |  |  |  |  |  |  |

TABLE S2 cont.

| No | Parent mass | RT | Area | Adduct | Formula |  | FBMN | | | | |  | MSFINDER Annotation TOP 5 | | | |  | CFM-ID 3.0 | | | | |  | Final Annotation |
| --- | --- | --- | --- | --- | --- | --- | --- | --- | --- | --- | --- | --- | --- | --- | --- | --- | --- | --- | --- | --- | --- | --- | --- | --- |
|  |  |  |  |  |  |  | Name | Smiles | MQ Score | Source | Network |  | Name | Smiles | Score | Source |  | Name | Smiles | Score | Formula | Source |  |  |
| 52 | 388.1618 | 4.06 | 25238 | [M+H]^+^ | C_21_H_23_ClFN_3_O |  | **—** | **—** | **—** | **—** | 13 |  | Flurazepam (*) | O=C1N(C=2C=CC(Cl)=CC2C(=NC1)C=3C=CC=CC3F)CCN(CC)CC (*) | 3.77 | HMDB=HMDB0014828, not found |  | *N*-[2-(3,4-dimethoxyphenyl)ethyl]-3-(2-hydroxy-3,4-dimethoxyphenyl)prop-2-enimidic acid | OC1=C(C(OC)=CC=C1/C=C/C(O)=N/CCC2=CC(OC)=C(C=C2)OC)OC | 0.94 | C_21_H_25_NO_6_ | HMDB=HMDB0135468, not found |  | Flurazepam |
|  |  |  |  |  |  |  |  |  |  |  |  |  | 2-(1-adamantyl)-4-chloro-5-[(4-fluorophenyl)methylamino]pyridazin-3-one | O=C1C(Cl)=C(C=NN1C23CC4CC(CC(C4)C2)C3)NCC5=CC=C(F)C=C5 | 3.64 | ChEBI=CHEBI:109676, not found |  |  |  |  |  |  |  |  |

* Represents the annotation shared by MSFINDER and MSDIAL.

TABLE S3 Multi database annotation results of compounds with high scores and high yields in other networks besides Network 5, 7, 9, 12, and 13

| No | Parent mass | RT | Area | Adduct | Formula |  | FBMN | | | | |  | | MSFINDER Annotation TOP 5 | | | | |  | | CFM-ID 3.0 | | | | | |  | Final Annotation |
| --- | --- | --- | --- | --- | --- | --- | --- | --- | --- | --- | --- | --- | --- | --- | --- | --- | --- | --- | --- | --- | --- | --- | --- | --- | --- | --- | --- | --- |
|  |  |  |  |  |  |  | Name | Smiles | MQ Score | Source | Network |  | Name | | Smiles | Score | Source |  | | Name | | Smiles | Score | Formula | Source |  | |  |
| 1 | 381.2982 | 5.92 | 34286 | [M+Na]^+^ | C_21_H_42_O_4_ |  | **—** | **—** | **—** | **—** | 1 |  | Stearic acid glyceryl ester (*) | | O=C(OCC(O)CO)CCCCCCCCCCCCCCCCC (*) | 6.45 | Plantae |  | | 1,3-dihydroxypropan-2-yl octadecanoate | | CCCCCCCCCCCCCCCCCC(=O)OC(CO)CO | 0.89 | C_21_H_42_O_4_ | Plantae |  | | Stearic acid glyceryl ester |
|  |  |  |  |  |  |  |  |  |  |  |  |  | Glycerol Monoisostearate | | O=C(OCC(O)CO)CCCCCCCCCCCCCCC(C)C | 6.38 | Bacterium |  | |  | |  |  |  |  |  | |  |
|  |  |  |  |  |  |  |  |  |  |  |  |  | 2-Monostearin | | O=C(OC(CO)CO)CCCCCCCCCCCCCCCCC | 6.26 | Archaeplastida |  | |  | |  |  |  |  |  | |  |
|  |  |  |  |  |  |  |  |  |  |  |  |  | 2-(1-Hydroxyheptadecyl)-1,3-Dioxolane-4-Methanol | | OCC1OC(OC1)C(O)CCCCCCCCCCCCCCCC | 6.23 | Metazoa |  | |  | |  |  |  |  |  | |  |
|  |  |  |  |  |  |  |  |  |  |  |  |  | (1-hexadecoxy-3-hydroxypropan-2-yl) acetate | | O=C(OC(CO)COCCCCCCCCCCCCCCCC)C | 6.21 | COCONUT=CNP0105234, not found |  | |  | |  |  |  |  |  | |  |
| 2 | 540.316 | 3.83 | 5110 | [M+H]^+^ | C_28_H_45_NO_9_ |  | **—** | **—** | **—** | **—** | 2 |  | 3-(carbamoylmethyl)-11,13-dihydroxy-14,21-dimethoxy-8,10,12-trimethyl-7,21-dioxohenicosa-9,15,19-trienoic acid | | O=C(OC)C=CCCC=CC(OC)C(O)C(C)C(O)C(=CC(C(=O)CCCC(CC(=O)O)CC(=O)N)C)C | 5.52 | COCONUT=CNP0323388, not found |  | | **—** | | **—** | **—** | **—** | **—** |  | | 3-(carbamoylmethyl)-11,13-dihydroxy-14,21-dimethoxy-8,10,12-trimethyl-7,21-dioxohenicosa-9,15,19-trienoic acid |

TABLE S3 cont.

| No | Parent mass | RT | Area | Adduct | Formula |  | FBMN | | | | |  | MSFINDER Annotation TOP 5 | | | |  | CFM-ID 3.0 | | | | |  | Final Annotation |
| --- | --- | --- | --- | --- | --- | --- | --- | --- | --- | --- | --- | --- | --- | --- | --- | --- | --- | --- | --- | --- | --- | --- | --- | --- |
|  |  |  |  |  |  |  | Name | Smiles | MQ Score | Source | Network |  | Name | Smiles | Score | Source |  | Name | Smiles | Score | Formula | Source |  |  |
| 3 | 385.1198 | 5.50 | 20779 | [M+Na]^+^ | C_21_H_18_N_2_O_4_ |  | **—** | **—** | **—** | **—** | 2 |  | Zanthobisquinolone | O=C1C(=C(O)C=2C=CC=CC2N1C)CC=3C(=O)N(C=4C=CC=CC4C3O)C | 4.6 | Archaeplastida |  | 4-hydroxy-3-[(4-hydroxy-1-methyl-2-oxo-1,2-dihydroquinolin-3-yl)methyl]-1-methyl-1,2-dihydroquinolin-2-one | CN1C(=O)C(CC2=C(O)C3=CC=CC=C3N(C)C2=O)=C(O)C2=CC=CC=C12 | 0.92 | C_21_H_18_N_2_O_4_ | Archaeplastida |  | Zanthobisquinolone |
|  |  |  |  |  |  |  |  |  |  |  |  |  | 7-methyl-4-{2-oxo-1-azatricyclo[7.3.1.0⁵,¹³]trideca-3,5,7,9(13)-tetraen-3-yl}-2*H*,3*H*,4*H*,5*H*,6*H*-pyrano[3,2-c]pyridine-2,5-dione | O=C1OC=2C=C(NC(=O)C2C(C3=CC=4C=CC=C5C4N(C3=O)CCC5)C1)C | 4.47 | COCONUT=CNP0400659, not found |  |  |  |  |  |  |  |  |
|  |  |  |  |  |  |  |  |  |  |  |  |  | 6,7-dimethyl-4-{11-oxo-1-azatricyclo[6.3.1.0⁴,¹²]dodeca-4(12),5,7,9-tetraen-10-yl}-2*H*,3*H*,4*H*,5*H*,6*H*-pyrano[3,2-c]pyridine-2,5-dione | O=C1OC=2C=C(N(C(=O)C2C(C3=CC=4C=CC=C5C4N(C3=O)CC5)C1)C)C | 4.44 | COCONUT=CNP0403960, not found |  |  |  |  |  |  |  |  |
|  |  |  |  |  |  |  |  |  |  |  |  |  | 2-benzyl-9-(3,6-dioxocyclohexa-1,4-dien-1-yl)-1*H*,2*H*,3*H*,4*H*,6*H*,7*H*,8*H*-pyrido[1,2-a]pyrazine-1,4-dione (*) | O=C1C=CC(=O)C(=C1)C2=C3C(=O)N(CC=4C=CC=CC4)CC(=O)N3CCC2 (*) | 4.42 | COCONUT=CNP0379211, not found |  |  |  |  |  |  |  |  |
|  |  |  |  |  |  |  |  |  |  |  |  |  | Ethyl 2-amino-5-oxo-4-phenyl-4*H*,5*H*,6*H*-pyrano[3,2-c]quinoline-3-carboxylate | O=C(OCC)C1=C(OC=2C=3C=CC=CC3NC(=O)C2C1C=4C=CC=CC4)N | 4.41 | COCONUT=CNP0370422, not found |  |  |  |  |  |  |  |  |

TABLE S3 cont.

| No | Parent mass | RT | Area | Adduct | Formula |  | FBMN | | | | |  | MSFINDER Annotation TOP 5 | | | |  | CFM-ID 3.0 | | | | |  | Final Annotation |
| --- | --- | --- | --- | --- | --- | --- | --- | --- | --- | --- | --- | --- | --- | --- | --- | --- | --- | --- | --- | --- | --- | --- | --- | --- |
|  |  |  |  |  |  |  | Name | Smiles | MQ Score | Source | Network |  | Name | Smiles | Score | Source |  | Name | Smiles | Score | Formula | Source |  |  |
| 4 | 340.2838 | 3.78 | 4835 | [M+Na]^+^ | C_18_H_39_NO_3_ |  | **—** | **—** | **—** | **—** | 2 |  | Phytosphingosine (*) | OCC(N)C(O)C(O)CCCCCCCCCCCCCC (*) | 6.49 | Plantae |  | Phytosphingosine | CCCCCCCCCCCCCCC(C(C(CO)N)O)O | 0.96 | C_18_H_39_NO_3_ | Metazoa |  | Phytosphingosine |
|  |  |  |  |  |  |  |  |  |  |  |  |  | 2-aminooctadecane-1,3,6-triol | OCC(N)C(O)CCC(O)CCCCCCCCCCCC | 5.6 | Metazoa |  |  |  |  |  |  |  |  |
| 5 | 340.2801 | 3.83 | 2499 | [M+Na]^+^ | C_18_H_39_NO_3_ |  | **—** | **—** | **—** | **—** | 2 |  | 2-aminooctadecane-1,3,4-triol (*) | OCC(N)C(O)C(O)CCCCCCCCCCCCCC (*) | 7.11 | Plantae |  | Phytosphingosine | CCCCCCCCCCCCCCC(C(C(CO)N)O)O | 0.96 | C_18_H_39_NO_3_ | Metazoa |  | 2-aminooctadecane-1,3,4-triol |
|  |  |  |  |  |  |  |  |  |  |  |  |  | 2-aminooctadecane-1,3,6-triol | OCC(N)C(O)CCC(O)CCCCCCCCCCCC | 6.23 | Metazoa |  |  |  |  |  |  |  |  |
| 6 | 465.2275 | 3.69 | 17102 | [M+Na]^+^ | C_26_H_34_O_6_ |  | **—** | **—** | **—** | **—** | 2 |  | Sch 528647 | O=C(O)C=CC=CC=CC=CC(=O)OC1CCC(=C)C(C1OC)C2(OC2CC=C(C)C)C | 5 | Fungi |  | **—** | **—** | **—** | **—** | **—** |  | Sch 528647 |
|  |  |  |  |  |  |  |  |  |  |  |  |  | Strobilurin M | O=C(OC)C(=COC)C(=CC=CC1=CC=C2OCC(OC2=C1)(OCC=C(C)C)C(C)C)C | 4.91 | Fungi |  |  |  |  |  |  |  |  |

TABLE S3 cont.

| No | Parent mass | RT | Area | Adduct | Formula |  | FBMN | | | | |  | MSFINDER Annotation TOP 5 | | | |  | CFM-ID 3.0 | | | | |  | Final Annotation |
| --- | --- | --- | --- | --- | --- | --- | --- | --- | --- | --- | --- | --- | --- | --- | --- | --- | --- | --- | --- | --- | --- | --- | --- | --- |
|  |  |  |  |  |  |  | Name | Smiles | MQ Score | Source | Network |  | Name | Smiles | Score | Source |  | Name | Smiles | Score | Formula | Source |  |  |
|  |  |  |  |  |  |  |  |  |  |  |  |  | Strobilurin G | O=C(OC)C(=COC)C(=CC=CC1=CC=C2OCC(OCC=C(C)C)C(OC2=C1)(C)C)C | 4.88 | Fungi |  |  |  |  |  |  |  |  |
|  |  |  |  |  |  |  |  |  |  |  |  |  | Englerin A | O=C(OC1C2C(C)CCC2C3(OC1(CC3OC(=O)CO)C(C)C)C)C=CC=4C=CC=CC4 | 4.85 | Archaeplastida |  |  |  |  |  |  |  |  |
|  |  |  |  |  |  |  |  |  |  |  |  |  | Pectachol | O=C1OC=2C(OC)=C(OCC3C(=C)CCC4C(C)(C)C(O)CCC34C)C(OC)=CC2C=C1 | 4.83 | Plantae |  |  |  |  |  |  |  |  |
| 7 | 701.4943 | 0.71 | 1399522 | [M+H]^+^ | C_39_H_73_O_8_P |  | **—** | **—** | **—** | **—** | 2 |  | **—** | **—** | **—** | **—** |  | PA(20:1(11*Z*)/16:1(9*Z*)) | CCCCCCCCC=CCCCCCCCCCC(=O)OCC(COP(=O)(O)O)OC(=O)CCCCCCCC=CCCCCCC | 0.4 | C_39_H_73_O_8_P | PubChem=52929126, not found |  | PA(20:1(11*Z*)/16:1(9*Z*)) |

TABLE S3 cont.

| No | Parent mass | RT | Area | Adduct | Formula |  | FBMN | | | | |  | MSFINDER Annotation TOP 5 | | | |  | CFM-ID 3.0 | | | | |  | Final Annotation |
| --- | --- | --- | --- | --- | --- | --- | --- | --- | --- | --- | --- | --- | --- | --- | --- | --- | --- | --- | --- | --- | --- | --- | --- | --- |
|  |  |  |  |  |  |  | Name | Smiles | MQ Score | Source | Network |  | Name | Smiles | Score | Source |  | Name | Smiles | Score | Formula | Source |  |  |
| 8 | 475.3276 | 0.63 | 222378 | [M+H]^+^ | C_28_H_42_O_6_ |  | **—** | **—** | **—** | **—** | 2 |  | **—** | **—** | **—** | **—** |  | 4-[1-(4,7-dihydroxy-10,13-dimethyl-1-oxo-2,3,4,5,6,7,8,9,11,12,14,15,16,17-tetradecahydrocyclopenta[a]phenanthren-17-yl)ethyl]-1,6-dimethyl-3,7-dioxabicyclo[4.1.0]heptan-2-one | CC(C1CCC2C3C(O)CC4C(O)CCC(=O)C4(C)C3CCC12C)C1CC2(C)OC2(C)C(=O)O1 | 0.99 | C_28_H_42_O_6_ | Plantae |  | 4-[1-(4,7-dihydroxy-10,13-dimethyl-1-oxo-2,3,4,5,6,7,8,9,11,12,14,15,16,17-tetradecahydrocyclopenta[a]phenanthren-17-yl)ethyl]-1,6-dimethyl-3,7-dioxabicyclo[4.1.0]heptan-2-one |
| 9 | 313.2741 | 7.15 | 427737 | [M-H_2_O+H]^+^ | C_19_H_38_O_4_ |  | **—** | **—** | **—** | **—** | 3 |  | Glyceryl Palmitate (*) | O=C(OCC(O)CO)CCCCCCCCCCCCCCC (*) | 6.44 | Plantae |  | **—** | **—** | **—** | **—** | **—** |  | Glyceryl Palmitate |
|  |  |  |  |  |  |  |  |  |  |  |  |  | 2-Palmitoylglycerol | O=C(OC(CO)CO)CCCCCCCCCCCCCCC | 6.2 | Archaeplastida |  |  |  |  |  |  |  |  |
|  |  |  |  |  |  |  |  |  |  |  |  |  | 2,4-dihydroxyheptadecyl acetate | O=C(OCC(O)CC(O)CCCCCCCCCCCCC)C | 6.07 | Tracheophyta |  |  |  |  |  |  |  |  |
|  |  |  |  |  |  |  |  |  |  |  |  |  | methyl 9,10-dihydroxyoctadecanoate | O=C(OC)CCCCCCCC(O)C(O)CCCCCCCC | 6.07 | Archaeplastida |  |  |  |  |  |  |  |  |
|  |  |  |  |  |  |  |  |  |  |  |  |  | 1-[2-(4,8-dimethylnonyl)-5-(1-hydroxyethyl)-2-methyl-1,3-dioxolan-4-yl]ethan-1-ol | OC(C)C1OC(OC1C(O)C)(C)CCCC(C)CCCC(C)C | 6.01 | COCONUT=CNP0117356, not found |  |  |  |  |  |  |  |  |

TABLE S3 cont.

| No | Parent mass | RT | Area | Adduct | Formula |  | FBMN | | | | |  | MSFINDER Annotation TOP 5 | | | |  | CFM-ID 3.0 | | | | |  | Final Annotation |
| --- | --- | --- | --- | --- | --- | --- | --- | --- | --- | --- | --- | --- | --- | --- | --- | --- | --- | --- | --- | --- | --- | --- | --- | --- |
|  |  |  |  |  |  |  | Name | Smiles | MQ Score | Source | Network |  | Name | Smiles | Score | Source |  | Name | Smiles | Score | Formula | Source |  |  |
| 10 | 313.2741 | 7.26 | 185911 | [M-H_2_O+H]^+^ | C_19_H_38_O_4_ |  | **—** | **—** | **—** | **—** | 3 |  | Glyceryl Palmitate (*) | O=C(OCC(O)CO)CCCCCCCCCCCCCCC (*) | 5.39 | Plantae |  | **—** | **—** | **—** | **—** | **—** |  | Glyceryl Palmitate |
|  |  |  |  |  |  |  |  |  |  |  |  |  | 2-Palmitoylglycerol | O=C(OC(CO)CO)CCCCCCCCCCCCCCC | 5.17 | Archaeplastida |  |  |  |  |  |  |  |  |
|  |  |  |  |  |  |  |  |  |  |  |  |  | 2-(2,3-dihydroxypropyl)hexadecanoic acid | O=C(O)C(CCCCCCCCCCCCCC)CC(O)CO | 4.99 | Archaeplastida |  |  |  |  |  |  |  |  |
|  |  |  |  |  |  |  |  |  |  |  |  |  | methyl 9,10-dihydroxyoctadecanoate | O=C(OC)CCCCCCCC(O)C(O)CCCCCCCC | 4.98 | Archaeplastida |  |  |  |  |  |  |  |  |
|  |  |  |  |  |  |  |  |  |  |  |  |  | Valsafungin A | O=C(O)C(CO)CCCCCCCCCC(O)CCCCCC | 4.96 | Fungi |  |  |  |  |  |  |  |  |
| 11 | 331.2844 | 7.15 | 33551 | [M+H]^+^ | C_19_H_38_O_4_ |  | **—** | **—** | **—** | **—** | 3 |  | Glyceryl Palmitate (*) | O=C(OCC(O)CO)CCCCCCCCCCCCCCC (*) | 6.70 | Plantae |  | 2-Palmitoylglycerol | CCCCCCCCCCCCCCCC(=O)OC(CO)CO | 0.68 | C_19_H_38_O_4_ | Archaeplastida |  | Glyceryl Palmitate |
|  |  |  |  |  |  |  |  |  |  |  |  |  | 2-Palmitoylglycerol | O=C(OC(CO)CO)CCCCCCCCCCCCCCC | 6.46 | Archaeplastida |  |  |  |  |  |  |  |  |
|  |  |  |  |  |  |  |  |  |  |  |  |  | 1-[2-(4,8-dimethylnonyl)-5-(1-hydroxyethyl)-2-methyl-1,3-dioxolan-4-yl]ethan-1-ol | OC(C)C1OC(OC1C(O)C)(C)CCCC(C)CCCC(C)C | 6.28 | COCONUT=CNP0117356, not found |  |  |  |  |  |  |  |  |
|  |  |  |  |  |  |  |  |  |  |  |  |  | methyl 9,10-dihydroxyoctadecanoate | O=C(OC)CCCCCCCC(O)C(O)CCCCCCCC | 6.28 | Archaeplastida |  |  |  |  |  |  |  |  |
|  |  |  |  |  |  |  |  |  |  |  |  |  | 2,4-dihydroxyheptadecyl acetate | O=C(OCC(O)CC(O)CCCCCCCCCCCCC)C | 6.27 | Tracheophyta |  |  |  |  |  |  |  |  |

TABLE S3 cont.

| No | Parent mass | RT | Area | Adduct | Formula |  | FBMN | | | | |  | MSFINDER Annotation TOP 5 | | | |  | CFM-ID 3.0 | | | | |  | Final Annotation |
| --- | --- | --- | --- | --- | --- | --- | --- | --- | --- | --- | --- | --- | --- | --- | --- | --- | --- | --- | --- | --- | --- | --- | --- | --- |
|  |  |  |  |  |  |  | Name | Smiles | MQ Score | Source | Network |  | Name | Smiles | Score | Source |  | Name | Smiles | Score | Formula | Source |  |  |
| 12 | 304.1276 | 4.00 | 12420 | [M+Na]^+^ | C_18_H_19_NO_2_ |  | **—** | **—** | **—** | **—** | 4 |  | Floribundine | OC=1C=C2C3=C(C1OC)C4=CC=CC=C4CC3N(C)CC2 | 4.85 | Plantae |  | Floribundine | OC=1C=C2C3=C(C1OC)C4=CC=CC=C4CC3N(C)CC2 | 0.63 | C_18_H_19_NO_2_ | Archaeplastida |  | Floribundine |
|  |  |  |  |  |  |  |  |  |  |  |  |  | 4-methoxy-10-methyl-10-azatetracyclo[7.7.1.0²,⁷.0¹³,¹⁷]heptadeca-1(16),2(7),3,5,13(17),14-hexaen-5-ol | OC=1C=C2C(=CC1OC)C=3C=CC=C4C3C(N(C)CC4)C2 | 4.5 | Archaeplastida |  |  |  |  |  |  |  |  |
|  |  |  |  |  |  |  |  |  |  |  |  |  | Bharatamine | OC=1C=C2C(=CC1OC)CCN3CC=4C=CC=CC4CC23 | 4.48 | Plantae |  |  |  |  |  |  |  |  |
|  |  |  |  |  |  |  |  |  |  |  |  |  | 10-methoxy-4-methyl-12-oxa-4-azapentacyclo[9.6.1.0¹,¹³.0⁵,¹⁷.0⁷,¹⁸]octadeca-7,9,11(18),14,16-pentaene | O(C1=CC=C2C3=C1OC4C=CC=C5C(N(C)CCC534)C2)C | 4.46 | COCONUT=CNP0148311, not found |  |  |  |  |  |  |  |  |
| 13 | 376.1489 | 4.00 | 6579 | [M+H]^+^ | C_21_H_23_ClFNO_2_ |  | **—** | **—** | **—** | **—** | 4 |  | Haloperidol (*) | O=C(C1=CC=C(F)C=C1)CCCN2CCC(O)(C3=CC=C(Cl)C=C3)CC2 (*) | 4.91 | HMDB=HMDB0014645, not found |  | methyl 2-[4-[2-[(4-chlorobenzoyl)amino]ethyl]phenoxy]-2-methylpropanoate | CC(C)(C(OC)=O)OC1=CC=C(CCNC(C2=CC=C(Cl)C=C2)=O)C=C1 | 0.63 | C_20_H_22_ClNO_4_ | Plantae |  | Haloperidol |
| 14 | 302.1324 | 4.00 | 39243 | [M+H]^+^ | C_19_H_15_N_3_O |  | **—** | **—** | **—** | **—** | 4 |  | 3-phenyl-1,4-bis(pyridin-4-yl)azetidin-2-one | O=C1N(C=2C=CN=CC2)C(C=3C=CN=CC3)C1C=4C=CC=CC4 | 4.09 | COCONUT=CNP0266816, not found |  | 1-[(3,4-dihydroxyphenyl)methyl]-2-methyl-1,2,3,4-tetrahydroisoquinoline-6,7-diol | CN1CCC2=CC(=C(C=C2C1CC3=CC(=C(C=C3)O)O)O)O | 0.56 | C_17_H_19_NO_4_ | Plantae |  | 21-methyl-3,13,21-triazapentacyclo[11.8.0.0²,¹⁰.0⁴,⁹.0¹⁵,²⁰]henicosa-1,3,5,7,9,15,17,19-octaen-14-one |

TABLE S3 cont.

| No | Parent mass | RT | Area | Adduct | Formula |  | FBMN | | | | |  | MSFINDER Annotation TOP 5 | | | |  | CFM-ID 3.0 | | | | |  | Final Annotation |
| --- | --- | --- | --- | --- | --- | --- | --- | --- | --- | --- | --- | --- | --- | --- | --- | --- | --- | --- | --- | --- | --- | --- | --- | --- |
|  |  |  |  |  |  |  | Name | Smiles | MQ Score | Source | Network |  | Name | Smiles | Score | Source |  | Name | Smiles | Score | Formula | Source |  |  |
|  |  |  |  |  |  |  |  |  |  |  |  |  | 21-methyl-3,13,21-triazapentacyclo[11.8.0.0²,¹⁰.0⁴,⁹.0¹⁵,²⁰]henicosa-1,3,5,7,9,15,17,19-octaen-14-one (*) | O=C1C=2C=CC=CC2N(C3=C4N=C5C=CC=CC5=C4CCN13)C (*) | 4.08 | Plantae |  |  |  |  |  |  |  |  |
|  |  |  |  |  |  |  |  |  |  |  |  |  | 3-methyl-7-(4-methylphenyl)-4-phenyl-[1,2]oxazolo[3,4-d]pyridazine | N=1N=C(C=2C=CC=CC2)C=3C(=NOC3C)C1C4=CC=C(C=C4)C | 4.07 | COCONUT=CNP0057236, not found |  |  |  |  |  |  |  |  |
|  |  |  |  |  |  |  |  |  |  |  |  |  | 1-methyl-*N*-phenyl-9*H*-pyrido[3,4-b]indole-3-carboxamide | O=C(NC=1C=CC=CC1)C=2N=C(C=3NC=4C=CC=CC4C3C2)C | 3.96 | COCONUT=CNP0368730, not found |  |  |  |  |  |  |  |  |
|  |  |  |  |  |  |  |  |  |  |  |  |  | 18-methyl-3,13,17-triazapentacyclo[11.8.0.0²,¹⁰.0⁴,⁹.0¹⁵,²⁰]henicosa-1(21),2(10),4,6,8,15(20),16,18-octaen-14-one | O=C1C2=CN=C(C=C2C=C3C=4NC=5C=CC=CC5C4CCN13)C | 3.92 | Archaeplastida |  |  |  |  |  |  |  |  |
| 15 | 398.1922 | 4.01 | 8237 | [M+H]^+^ | C_23_H_27_NO_5_ |  | **—** | **—** | **—** | **—** | 4 |  | 8,16-dimethoxy-12,13,13-trimethyl-16-(3-methylbut-2-en-1-yl)-4,14-dioxa-2-azatetracyclo[7.7.0.0³,⁷.0¹¹,¹⁵]hexadeca-1(9),2,5,7,11(15)-pentaen-10-one | O=C1C=2C(OC)=C3C=COC3=NC2C(OC)(C=4OC(C)(C)C(C14)C)CC=C(C)C | 4.95 | Archaeplastida |  | 3-(6-imidazol-1-yl-4-methyl-1*H*-benzimidazol-2-yl)-4-(pyridin-2-ylmethylamino)-1*H*-pyridin-2-one | CC1=CC(=CC2=C1N=C(N2)C1=C(NCC2=NC=CC=C2)C=CNC1=O)N1C=CN=C1 | 0.72 | C_22_H_19_N_7_O | PubChem=135530418, not found |  | 8,16-dimethoxy-12,13,13-trimethyl-16-(3-methylbut-2-en-1-yl)-4,14-dioxa-2-azatetracyclo[7.7.0.0³,⁷.0¹¹,¹⁵]hexadeca-1(9),2,5,7,11(15)-pentaen-10-one |

TABLE S3 cont.

| No | Parent mass | RT | Area | Adduct | Formula |  | FBMN | | | | |  | MSFINDER Annotation TOP 5 | | | |  | CFM-ID 3.0 | | | | |  | Final Annotation |
| --- | --- | --- | --- | --- | --- | --- | --- | --- | --- | --- | --- | --- | --- | --- | --- | --- | --- | --- | --- | --- | --- | --- | --- | --- |
|  |  |  |  |  |  |  | Name | Smiles | MQ Score | Source | Network |  | Name | Smiles | Score | Source |  | Name | Smiles | Score | Formula | Source |  |  |
|  |  |  |  |  |  |  |  |  |  |  |  |  | 2-[(5-hydroxy-2,2-dimethyl-4-oxo-3,4-dihydro-2*H*-1-benzopyran-7-yl)oxy]-*N*-(4-phenylbutan-2-yl)acetamide | O=C(NC(C)CCC=1C=CC=CC1)COC=2C=C(O)C3=C(OC(C)(C)CC3=O)C2 | 4.9 | COCONUT=CNP0355562, not found |  |  |  |  |  |  |  |  |
|  |  |  |  |  |  |  |  |  |  |  |  |  | 7-[2-(4a-hydroxy-decahydroisoquinolin-2-yl)-2-oxoethoxy]-1*H*,2*H*,3*H*,4*H*-cyclopenta[c]chromen-4-one | O=C1OC=2C=C(OCC(=O)N3CCC4(O)CCCCC4C3)C=CC2C5=C1CCC5 | 4.9 | COCONUT=CNP0415547, not found |  |  |  |  |  |  |  |  |
|  |  |  |  |  |  |  |  |  |  |  |  |  | Vilmorrianone | O=C(OC1CC2(C)CN(C)C3C4C(=O)C5C(=C)CC64C(=O)C(=O)C2C3(C1)C6C5)C | 4.79 | Plantae |  |  |  |  |  |  |  |  |
| 16 | 346.1562 | 4.01 | 25944 | [M+Na]^+^ | C_17_H_25_NO_5_ |  | **—** | **—** | **—** | **—** | 4 |  | 7-hydroxy-4,5,6-trimethyl-2,9-dioxa-14-azatricyclo[9.5.1.0¹⁴,¹⁷]heptadec-11-ene-3,8-dione | O=C1OCC2=CCN3CCC(OC(=O)C(C)C(C)C(C)C1O)C23 | 5.26 | Archaeplastida |  | Koenigicine | COC1=C(OC)C=C2C(NC3=C4C=CC(C)(C)OC4=C(C)C=C23)=C1 | 0.63 | C_20_H_21_NO_3_ | Archaeplastida |  | 7-hydroxy-4,5,6-trimethyl-2,9-dioxa-14-azatricyclo[9.5.1.0¹⁴,¹⁷]heptadec-11-ene-3,8-dione |
|  |  |  |  |  |  |  |  |  |  |  |  |  | 6-(1-hydroxyethyl)-4,5-dimethyl-2,8-dioxa-13-azatricyclo[8.5.1.0¹³,¹⁶]hexadec-10-ene-3,7-dione | O=C1OCC2=CCN3CCC(OC(=O)C(C)C(C)C1C(O)C)C23 | 5.25 | Archaeplastida |  |  |  |  |  |  |  |  |
|  |  |  |  |  |  |  |  |  |  |  |  |  | *N*-[(10-hydroxy-4,8-dimethyl-13-oxo-3,14-dioxatricyclo[9.3.0.02,4]tetradec-7-en-12-yl)methyl]acetamide | O=C(NCC1C(=O)OC2C3OC3(C)CCC=C(C)CC(O)C21)C | 5.22 | COCONUT=CNP0265429, not found |  |  |  |  |  |  |  |  |

TABLE S3 cont.

| No | Parent mass | RT | Area | Adduct | Formula |  | FBMN | | | | |  | MSFINDER Annotation TOP 5 | | | |  | CFM-ID 3.0 | | | | |  | Final Annotation |
| --- | --- | --- | --- | --- | --- | --- | --- | --- | --- | --- | --- | --- | --- | --- | --- | --- | --- | --- | --- | --- | --- | --- | --- | --- |
|  |  |  |  |  |  |  | Name | Smiles | MQ Score | Source | Network |  | Name | Smiles | Score | Source |  | Name | Smiles | Score | Formula | Source |  |  |
|  |  |  |  |  |  |  |  |  |  |  |  |  | 5-Hydroxy-9-Methylstreptimidone | O=C1NC(=O)CC(C1)CC(O)CC(=O)C(O)(C=C(C=CC)C)C | 5.17 | Bacterium |  |  |  |  |  |  |  |  |
|  |  |  |  |  |  |  |  |  |  |  |  |  | 4-ethylidene-6-hydroxy-5,6-dimethyl-2,8-dioxa-13-azatricyclo[8.5.1.0¹³,¹⁶]hexadecane-3,7-dione | O=C1OC2CCN3CCC(COC(=O)C(O)(C)C(C1=CC)C)C32 | 5.17 | Archaeplastida |  |  |  |  |  |  |  |  |
| 17 | 348.1542 | 4.01 | 8359 | [M+H]^+^ | C_17_H_21_N_3_O_5_ |  | **—** | **—** | **—** | **—** | 4 |  | Oxepinamide A (*) | O=C1C=2C=C(OC)C=COC2N=C3N1C(C(=O)NC3(O)C(C)CC)C (*) | 5.68 | Fungi |  | **—** | **—** | **—** | **—** | **—** |  | Oxepinamide A |
|  |  |  |  |  |  |  |  |  |  |  |  |  | 3-[[4-[2-(furan-2-ylmethylamino)-2-oxoethyl]piperidin-3-yl]methyl]-1,2-oxazole-5-carboxylic acid | O=C(O)C=1ON=C(C1)CC2CNCCC2CC(=O)NCC=3OC=CC3 | 5.44 | COCONUT=CNP0051087, not found |  |  |  |  |  |  |  |  |
|  |  |  |  |  |  |  |  |  |  |  |  |  | 5-(6,7-dimethoxy-1,2,3,4-tetrahydroisoquinolin-1-yl)-3-ethyl-6-hydroxy-1,2,3,4-tetrahydropyrimidine-2,4-dione | O=C1NC(O)=C(C(=O)N1CC)C2NCCC3=CC(OC)=C(OC)C=C32 | 5.33 | COCONUT=CNP0363615, not found |  |  |  |  |  |  |  |  |
|  |  |  |  |  |  |  |  |  |  |  |  |  | *N*-{3-[(carbamoylmethyl)carbamoyl]-5,6-dihydroxycyclohex-2-en-1-yl}-4-methylbenzamide | O=C(N)CNC(=O)C1=CC(NC(=O)C2=CC=C(C=C2)C)C(O)C(O)C1 | 5.33 | COCONUT=CNP0057856, not found |  |  |  |  |  |  |  |  |
|  |  |  |  |  |  |  |  |  |  |  |  |  | *N*-(2-hydroxy-3-methoxypropyl)-2-({9-oxo-1*H*,2*H*,3*H*,9*H*-pyrrolo[2,1-b]quinazolin-7-yl}oxy)acetamide | O=C(NCC(O)COC)COC=1C=CC=2N=C3N(C(=O)C2C1)CCC3 | 5.24 | COCONUT=CNP0130413, not found |  |  |  |  |  |  |  |  |

TABLE S3 cont.

| No | Parent mass | RT | Area | Adduct | Formula |  | FBMN | | | | |  | MSFINDER Annotation TOP 5 | | | |  | CFM-ID 3.0 | | | | |  | Final Annotation |
| --- | --- | --- | --- | --- | --- | --- | --- | --- | --- | --- | --- | --- | --- | --- | --- | --- | --- | --- | --- | --- | --- | --- | --- | --- |
|  |  |  |  |  |  |  | Name | Smiles | MQ Score | Source | Network |  | Name | Smiles | Score | Source |  | Name | Smiles | Score | Formula | Source |  |  |
| 18 | 374.1513 | 4.00 | 23013 | [M+H]^+^ | C_22_H_19_N_3_O_3_ |  | **—** | **—** | **—** | **—** | 4 |  | Sebastianine B | O=C1N2C3=C(C=4N=C5C=CC=CC5=C6C=CN=C(C46)C3(O)OC1C(C)C)CC2 | 5.34 | Animalia |  | Prochlorperazine | CN1CCN(CCCN2C3=CC=CC=C3SC3=C2C=C(Cl)C=C3)CC1 | 0.94 | C_20_H_24_ClN_3_S | COCONUT=CNP0410106, not found |  | Sebastianine B |
|  |  |  |  |  |  |  |  |  |  |  |  |  | 10-(4-hydroxyphenyl)-6-methyl-14-(propan-2-yl)-8-oxa-13,14,16-triazatetracyclo[7.7.0.0²,⁷.0¹¹,¹⁵]hexadeca-1(16),2,4,6,9,11(15),12-heptaen-5-ol | OC=1C=CC(=CC1)C=2C=3OC4=C(C=CC(O)=C4C)C3N=C5C2C=NN5C(C)C | 5.13 | COCONUT=CNP0323782, not found |  |  |  |  |  |  |  |  |
|  |  |  |  |  |  |  |  |  |  |  |  |  | 10-(3-hydroxyphenyl)-6-methyl-14-(propan-2-yl)-8-oxa-13,14,16-triazatetracyclo[7.7.0.0²,⁷.0¹¹,¹⁵]hexadeca-1(16),2,4,6,9,11(15),12-heptaen-5-ol | OC1=CC=CC(=C1)C=2C=3OC4=C(C=CC(O)=C4C)C3N=C5C2C=NN5C(C)C | 5.13 | COCONUT=CNP0340852, not found |  |  |  |  |  |  |  |  |
|  |  |  |  |  |  |  |  |  |  |  |  |  | 13-(4-methylphenyl)-12,14-dioxo-1,13-diazatetracyclo[8.6.0.0²,⁷.0¹¹,¹⁵]hexadeca-2,4,6,8-tetraene-16-carboxamide | O=C(N)C1N2C=3C=CC=CC3C=CC2C4C(=O)N(C5=CC=C(C=C5)C)C(=O)C14 | 5.11 | COCONUT=CNP0178449, not found |  |  |  |  |  |  |  |  |

TABLE S3 cont.

| No | Parent mass | RT | Area | Adduct | Formula |  | FBMN | | | | |  | MSFINDER Annotation TOP 5 | | | |  | CFM-ID 3.0 | | | | |  | Final Annotation |
| --- | --- | --- | --- | --- | --- | --- | --- | --- | --- | --- | --- | --- | --- | --- | --- | --- | --- | --- | --- | --- | --- | --- | --- | --- |
|  |  |  |  |  |  |  | Name | Smiles | MQ Score | Source | Network |  | Name | Smiles | Score | Source |  | Name | Smiles | Score | Formula | Source |  |  |
|  |  |  |  |  |  |  |  |  |  |  |  |  | 10-(2-methoxyphenyl)-14-(propan-2-yl)-8-oxa-13,14,16-triazatetracyclo[7.7.0.0²,⁷.0¹¹,¹⁵]hexadeca-1(16),2,4,6,9,11(15),12-heptaen-5-ol (*) | OC=1C=CC2=C(OC3=C2N=C4C(C=NN4C(C)C)=C3C=5C=CC=CC5OC)C1 (*) | 5.08 | COCONUT=CNP0380717, not found |  |  |  |  |  |  |  |  |
| 19 | 356.1879 | 4.01 | 4863 | [M+H]^+^ | C_21_H_25_NO_4_ |  | **—** | **—** | **—** | **—** | 4 |  | Monascopyridine A | O=C1OC2(C(=O)C3=CN=C(C=CC)C=C3CC2C1C(=O)CCCCC)C | 5.11 | Fungi |  | **—** | **—** | **—** | **—** | **—** |  | Monascopyridine A |
|  |  |  |  |  |  |  |  |  |  |  |  |  | 6-[2-(4-hydroxyphenyl)ethyl]-7-methyl-4-(2-methylpropyl)-2*H*,3*H*,4*H*,5*H*,6*H*-pyrano[3,2-c]pyridine-2,5-dione | O=C1OC=2C=C(N(C(=O)C2C(C1)CC(C)C)CCC3=CC=C(O)C=C3)C | 5.09 | COCONUT=CNP0407099, not found |  |  |  |  |  |  |  |  |
|  |  |  |  |  |  |  |  |  |  |  |  |  | Tolypocladenol A2 | O=C1NC(=CC2=CC=C(O)C=C2)C(O)=C1C(=O)C(C)CC(C)CC=CC | 5.02 | Fungi |  |  |  |  |  |  |  |  |
|  |  |  |  |  |  |  |  |  |  |  |  |  | 10,17-dihydroxy-4-(3-methylbut-2-en-1-yl)-12-oxa-4-azapentacyclo[9.6.1.0¹,¹³.0⁵,¹⁷.0⁷,¹⁸]octadeca-7,9,11(18)-trien-14-one | O=C1CCC2(O)C3N(CC=C(C)C)CCC42C5=C(OC14)C(O)=CC=C5C3 | 5.02 | COCONUT=CNP0144437, not found |  |  |  |  |  |  |  |  |
|  |  |  |  |  |  |  |  |  |  |  |  |  | Pentyl 3-(2-methylphenyl)-4-oxo-10-oxa-3-azatricyclo[5.2.1.0¹,⁵]dec-8-ene-6-carboxylate | O=C(OCCCCC)C1C2OC3(C=C2)CN(C(=O)C13)C=4C=CC=CC4C | 5.01 | COCONUT=CNP0372060, not found |  |  |  |  |  |  |  |  |

TABLE S3 cont.

| No | Parent mass | RT | Area | Adduct | Formula |  | FBMN | | | | |  | MSFINDER Annotation TOP 5 | | | |  | CFM-ID 3.0 | | | | |  | Final Annotation |
| --- | --- | --- | --- | --- | --- | --- | --- | --- | --- | --- | --- | --- | --- | --- | --- | --- | --- | --- | --- | --- | --- | --- | --- | --- |
|  |  |  |  |  |  |  | Name | Smiles | MQ Score | Source | Network |  | Name | Smiles | Score | Source |  | Name | Smiles | Score | Formula | Source |  |  |
| 20 | 388.1658 | 4.01 | 175113 | [M+H]^+^ | C_19_H_25_N_5_O_2_S |  | **—** | **—** | **—** | **—** | 4 |  | **—** | **—** | **—** | **—** |  | 2-Ketobenzothiazole 23 | C1CCC(C1)C(=O)NC(CCCN=C(N)N)C(=O)C2=NC3=CC=CC=C3S2 | 0.63 | C_19_H_25_N_5_O_2_S | PubChem=3348131, not found |  | 2-Ketobenzothiazole 23 |
| 21 | 679.5124 | 0.7 | 342626 | [M+H]^+^ | C_36_H_66_N_6_O_6_ |  | **—** | **—** | **—** | **—** | 10 |  | 3,6,12-*tri*(butan-2-yl)-9,15,18-*tris*(2-methylpropyl)-1,4,7,10,13,16-hexazacyclooctadecane-2,5,8,11,14,17-hexone | O=C1NC(C(=O)NC(C(=O)NC(C(=O)NC(C(=O)NC(C(=O)NC1CC(C)C)C(C)CC)C(C)CC)CC(C)C)C(C)CC)CC(C)C | 5.2 | Bacterium |  | **—** | **—** | **—** | **—** | **—** |  | 3,6,12-*tri*(butan-2-yl)-9,15,18-*tris*(2-methylpropyl)-1,4,7,10,13,16-hexazacyclooctadecane-2,5,8,11,14,17-hexone |
| 22 | 453.1642 | 4.16 | 75461 | [M+H]^+^ | C_22_H_28_O_10_ |  | **—** | **—** | **—** | **—** | 11 |  | Melampolide 2 | O=C(OC)C1=CCCC(=CC2OC(=O)C(=C)C2C(OC(=O)C(O)(C)CO)C1OC(=O)C)C | 4.87 | Archaeplastida |  | [2a,7-dihydroxy-3-(hydroxymethyl)-6,6,7b-trimethyl-1,2,4a,5,7,7a-hexahydrocyclobuta[e]inden-2-yl] 3-chloro-4,6-dihydroxy-2-methylbenzoate | CC1=C(C(=O)OC2CC3(C)C4C(O)C(C)(C)CC4C=C(CO)C23O)C(O)=CC(O)=C1Cl | 0.68 | C_23_H_29_ClO_7_ | Fungi |  | Melampolide 2 |
|  |  |  |  |  |  |  |  |  |  |  |  |  | Glochidacuminoside B | O=C(O)CC=1C=CC=CC1OC2OC(COC(=O)CC3=CCC(O)CC3)C(O)C(O)C2O | 4.87 | Plantae |  |  |  |  |  |  |  |  |
|  |  |  |  |  |  |  |  |  |  |  |  |  | Davisioside | O=C(OCC1=CC(O)C2CCOC(OC3OC(CO)C(O)C(O)C3O)C12)C=4C=CC=CC4 | 4.82 | Plantae |  |  |  |  |  |  |  |  |

TABLE S3 cont.

| No | Parent mass | RT | Area | Adduct | Formula |  | FBMN | | | | |  | MSFINDER Annotation TOP 5 | | | |  | CFM-ID 3.0 | | | | |  | Final Annotation |
| --- | --- | --- | --- | --- | --- | --- | --- | --- | --- | --- | --- | --- | --- | --- | --- | --- | --- | --- | --- | --- | --- | --- | --- | --- |
|  |  |  |  |  |  |  | Name | Smiles | MQ Score | Source | Network |  | Name | Smiles | Score | Source |  | Name | Smiles | Score | Formula | Source |  |  |
|  |  |  |  |  |  |  |  |  |  |  |  |  | 3,4,5-Trimethoxy-3',4'-Dithoxybibenzyl 3'-O-Glucoside | OC1=CC=C(C=C1OC2OC(CO)C(O)C(O)C2O)CCC3=CC(O)=C(OC)C(OC)=C3 | 4.79 | Plantae |  |  |  |  |  |  |  |  |
|  |  |  |  |  |  |  |  |  |  |  |  |  | Ethyl 2,10,15,16-tetrahydroxy-9,13-dimethyl-4,11-dioxo-5,18-dioxapentacyclo[12.5.0.0¹,⁶.0²,¹⁷.0⁸,¹³]nonadec-9-ene-17-carboxylate | O=C1OC2CC3C(=C(O)C(=O)CC3(C)C4C(O)C(O)C5(OCC24C5(O)C1)C(=O)OCC)C | 4.74 | COCONUT=CNP0096401, not found |  |  |  |  |  |  |  |  |
| 23 | 437.1938 | 4.16 | 219596 | [M+K]^+^ | C_21_H_34_O_7_ |  | **—** | **—** | **—** | **—** | 11 |  | Neorustmicin B | O=C1OC(CC)C(O)(C=C(C)CC(C)CC2(OC)OCC(O)(C(=O)C1C)C2)C | 5.76 | Bacterium |  | **—** | **—** | **—** | **—** | **—** |  | Neorustmicin B |
|  |  |  |  |  |  |  |  |  |  |  |  |  | Hypochoeroside K | OCC1OC(OC2C(=C)C3CC(C(=C)C)CCC3(C)C(O)C2)C(O)C(O)C1O | 5.72 | Plantae |  |  |  |  |  |  |  |  |
|  |  |  |  |  |  |  |  |  |  |  |  |  | 1-(1-hydroxyethyl)-9a,11a-dimethyl-1*H*,2*H*,3*H*,3a*H*,3b*H*,4*H*,6*H*,7*H*,8*H*,9*H*,9a*H*,9b*H*,10*H*,11*H*,11a*H*-cyclopenta[a]phenanthrene-1,3a,3b,7,10,11-hexol | OC1CC2=CCC3(O)C(C(O)C(O)C4(C)C(O)(CCC34O)C(O)C)C2(C)CC1 | 5.70 | COCONUT=CNP0210793, not found |  |  |  |  |  |  |  |  |
|  |  |  |  |  |  |  |  |  |  |  |  |  | 2-{[2-(5-hydroxy-4a,8-dimethyl-1,2,3,4,4a,5,6,8a-octahydronaphthalen-2-yl)prop-2-en-1-yl]oxy}-6-(hydroxymethyl)oxane-3,4,5-triol | OCC1OC(OCC(=C)C2CCC3(C)C(O)CC=C(C)C3C2)C(O)C(O)C1O | 5.60 | COCONUT=CNP0141317, not found |  |  |  |  |  |  |  |  |

TABLE S3 cont.

| No | Parent mass | RT | Area | Adduct | Formula |  | FBMN | | | | |  | MSFINDER Annotation TOP 5 | | | |  | CFM-ID 3.0 | | | | |  | Final Annotation |
| --- | --- | --- | --- | --- | --- | --- | --- | --- | --- | --- | --- | --- | --- | --- | --- | --- | --- | --- | --- | --- | --- | --- | --- | --- |
|  |  |  |  |  |  |  | Name | Smiles | MQ Score | Source | Network |  | Name | Smiles | Score | Source |  | Name | Smiles | Score | Formula | Source |  |  |
|  |  |  |  |  |  |  |  |  |  |  |  |  | 2-(hydroxymethyl)-6-{[7-(3-hydroxyprop-1-en-2-yl)-4a-methyl-1-methylidene-decahydronaphthalen-2-yl]oxy}oxane-3,4,5-triol | OCC(=C)C1CCC2(C)CCC(OC3OC(CO)C(O)C(O)C3O)C(=C)C2C1 | 5.59 | Plantae |  |  |  |  |  |  |  |  |
| 24 | 453.1686 | 4.21 | 163789 | [M+H]^+^ | C_22_H_28_O_10_ |  | **—** | **—** | **—** | **—** | 11 |  | Melampolide 2 | O=C(OC)C1=CCCC(=CC2OC(=O)C(=C)C2C(OC(=O)C(O)(C)CO)C1OC(=O)C)C | 4.07 | Archaeplastida |  | [2a,7-dihydroxy-3-(hydroxymethyl)-6,6,7b-trimethyl-1,2,4a,5,7,7a-hexahydrocyclobuta[e]inden-2-yl] 3-chloro-4,6-dihydroxy-2-methylbenzoate | CC1=C(C(=O)OC2CC3(C)C4C(O)C(C)(C)CC4C=C(CO)C23O)C(O)=CC(O)=C1Cl | 0.68 | C_23_H_29_ClO_7_ | Fungi |  | Melampolide 2 |
|  |  |  |  |  |  |  |  |  |  |  |  |  | Eupalinilide J | O=C(OC1CC(O)(CO)C2C(OC(=O)C)C3OC3(C)C2C4OC(=O)C(=C)C14)C(=CCO)C | 3.99 | Plantae |  |  |  |  |  |  |  |  |
|  |  |  |  |  |  |  |  |  |  |  |  |  | [4*R*-(4*R**, *S**,7*R**,10*S**)]- 4,6-*Bis*(Acetyloxy)-3-[(Acetyloxy)Methyl]-5,6,7,8,9,10-Hexahydro-7-Methoxy-6,10-Dimethyl-7,10-Epoxycyclodeca[B]Furan-2(4*H*)-One | O=C1OC2=CC3(OC(OC)(CC3)C(OC(=O)C)(C)CC(OC(=O)C)C2=C1COC(=O)C)C | 3.97 | Plantae |  |  |  |  |  |  |  |  |

TABLE S3 cont.

| No | Parent mass | RT | Area | Adduct | Formula |  | FBMN | | | | |  | MSFINDER Annotation TOP 5 | | | |  | CFM-ID 3.0 | | | | |  | Final Annotation |
| --- | --- | --- | --- | --- | --- | --- | --- | --- | --- | --- | --- | --- | --- | --- | --- | --- | --- | --- | --- | --- | --- | --- | --- | --- |
|  |  |  |  |  |  |  | Name | Smiles | MQ Score | Source | Network |  | Name | Smiles | Score | Source |  | Name | Smiles | Score | Formula | Source |  |  |
|  |  |  |  |  |  |  |  |  |  |  |  |  | Melcanthin C | O=C(OC)C1=CC(O)CC(=CC2OC(=O)C(=C)C2C(OC(=O)C(C)C)C1OC(=O)C)CO | 3.97 | Plantae |  |  |  |  |  |  |  |  |
|  |  |  |  |  |  |  |  |  |  |  |  |  | [8-acetyloxy-12-(acetyloxymethyl)-4,8-dimethyl-7,13-dioxo-3,14-dioxatricyclo[9.3.0.02,4]tetradec-11-en-10-yl] propanoate | O=C1OC2C(=C1COC(=O)C)C(OC(=O)CC)CC(OC(=O)C)(C(=O)CCC3(OC23)C)C | 3.97 | Plantae |  |  |  |  |  |  |  |  |
| 25 | 437.1938 | 4.21 | 631029 | [M+K]^+^ | C_21_H_34_O_7_ |  | **—** | **—** | **—** | **—** | 11 |  | Neorustmicin B | O=C1OC(CC)C(O)(C=C(C)CC(C)CC2(OC)OCC(O)(C(=O)C1C)C2)C | 5.76 | Bacterium |  | **—** | **—** | **—** | **—** | **—** |  | Neorustmicin B |
|  |  |  |  |  |  |  |  |  |  |  |  |  | Hypochoeroside K | OCC1OC(OC2C(=C)C3CC(C(=C)C)CCC3(C)C(O)C2)C(O)C(O)C1O | 5.72 | Plantae |  |  |  |  |  |  |  |  |
|  |  |  |  |  |  |  |  |  |  |  |  |  | 1-(1-hydroxyethyl)-9a,11a-dimethyl-1*H*,2*H*,3*H*,3a*H*,3b*H*,4*H*,6*H*,7*H*,8*H*,9*H*,9a*H*,9b*H*,10*H*,11*H*,11a*H*-cyclopenta[a]phenanthrene-1,3a,3b,7,10,11-hexol | OC1CC2=CCC3(O)C(C(O)C(O)C4(C)C(O)(CCC34O)C(O)C)C2(C)CC1 | 5.70 | COCONUT=CNP0210793, not found |  |  |  |  |  |  |  |  |

TABLE S3 cont.

| No | Parent mass | RT | Area | Adduct | Formula |  | FBMN | | | | |  | MSFINDER Annotation TOP 5 | | | |  | CFM-ID 3.0 | | | | |  | Final Annotation |
| --- | --- | --- | --- | --- | --- | --- | --- | --- | --- | --- | --- | --- | --- | --- | --- | --- | --- | --- | --- | --- | --- | --- | --- | --- |
|  |  |  |  |  |  |  | Name | Smiles | MQ Score | Source | Network |  | Name | Smiles | Score | Source |  | Name | Smiles | Score | Formula | Source |  |  |
|  |  |  |  |  |  |  |  |  |  |  |  |  | 2-{[2-(5-hydroxy-4a,8-dimethyl-1,2,3,4,4a,5,6,8a-octahydronaphthalen-2-yl)prop-2-en-1-yl]oxy}-6-(hydroxymethyl)oxane-3,4,5-triol | OCC1OC(OCC(=C)C2CCC3(C)C(O)CC=C(C)C3C2)C(O)C(O)C1O | 5.60 | COCONUT=CNP0141317, not found |  |  |  |  |  |  |  |  |
|  |  |  |  |  |  |  |  |  |  |  |  |  | 2-(hydroxymethyl)-6-{[7-(3-hydroxyprop-1-en-2-yl)-4a-methyl-1-methylidene-decahydronaphthalen-2-yl]oxy}oxane-3,4,5-triol | OCC(=C)C1CCC2(C)CCC(OC3OC(CO)C(O)C(O)C3O)C(=C)C2C1 | 5.59 | Plantae |  |  |  |  |  |  |  |  |
| 26 | 247.0572 | 4.00 | 10155 | [M+H]^+^ | C_13_H_10_O_5_ |  | **—** | **—** | **—** | **—** | 14 |  | Isopimpinellin | O=C1OC=2C(OC)=C3OC=CC3=C(OC)C2C=C1 | 4.92 | Plantae |  | Glycerophosphoglycerol | OCC(O)COP(=O)(O)OCC(O)CO | 0.63 | C_6_H_15_O_8_P | Fungi |  | Hispidin |
|  |  |  |  |  |  |  |  |  |  |  |  |  | Pimpinellin (*) | O=C1OC2=C3C=COC3=C(OC)C(OC)=C2C=C1 (*) | 4.71 | Plantae |  |  |  |  |  |  |  |  |
|  |  |  |  |  |  |  |  |  |  |  |  |  | Hispidin | O=C1OC(C=CC2=CC=C(O)C(O)=C2)=CC(O)=C1 | 4.71 | Fungi |  |  |  |  |  |  |  |  |
|  |  |  |  |  |  |  |  |  |  |  |  |  | 2,3',4,6-Tetrahydroxybenzophenone | O=C(C=1C=CC=C(O)C1)C=2C(O)=CC(O)=CC2O | 4.66 | Plantae |  |  |  |  |  |  |  |  |
|  |  |  |  |  |  |  |  |  |  |  |  |  | Monosporascol A | O=C1C2=COC=C2C(O)C3=CC(OC)=CC(O)=C13 | 4.56 | Fungi |  |  |  |  |  |  |  |  |

TABLE S3 cont.

| No | Parent mass | RT | Area | Adduct | Formula |  | FBMN | | | | |  | MSFINDER Annotation TOP 5 | | | |  | CFM-ID 3.0 | | | | |  | Final Annotation |
| --- | --- | --- | --- | --- | --- | --- | --- | --- | --- | --- | --- | --- | --- | --- | --- | --- | --- | --- | --- | --- | --- | --- | --- | --- |
|  |  |  |  |  |  |  | Name | Smiles | MQ Score | Source | Network |  | Name | Smiles | Score | Source |  | Name | Smiles | Score | Formula | Source |  |  |
| 27 | 232.0525 | 4.00 | 17965 | [M+H]^+^ | C_13_H_10_ClNO |  | **—** | **—** | **—** | **—** | 14 |  | 6-chloro-2,4-dimethylfuro[3,2-c]quinoline | ClC1=CC=CC=2C1=NC(=C3C=C(OC23)C)C | 4.47 | COCONUT=CNP0184780, not found |  | 1-(2,3-dichlorophenyl)piperazine | ClC1=C(Cl)C(N2CCNCC2)=CC=C1 | 0.95 | C_10_H_12_Cl_2_N_2_ | PubChem=851833, not found |  | 6-chloro-2,4-dimethylfuro[3,2-c]quinoline |
|  |  |  |  |  |  |  |  |  |  |  |  |  | *N*-[(4-chlorophenyl)(phenyl)methylidene]hydroxylamine | ClC1=CC=C(C=C1)C(=NO)C=2C=CC=CC2 | 4.31 | COCONUT=CNP0058854, not found |  |  |  |  |  |  |  |  |
|  |  |  |  |  |  |  |  |  |  |  |  |  | 2-[(4-chlorophenyl)iminomethyl]phenol | ClC1=CC=C(N=CC=2C=CC=CC2O)C=C1 | 4.31 | COCONUT=CNP0031048, not found |  |  |  |  |  |  |  |  |
|  |  |  |  |  |  |  |  |  |  |  |  |  | 3-(2-chlorophenyl)-1-(1*H*-pyrrol-2-yl)prop-2-en-1-one | O=C(C=CC=1C=CC=CC1Cl)C2=CC=CN2 | 4.3 | ChEBI=CHEBI:115516, not found |  |  |  |  |  |  |  |  |
| 28 | 415.2105 | 4.21 | 208800 | [M+H]^+^ | C_25_H_26_N_4_O_2_ |  | **—** | **—** | **—** | **—** | 17 |  | 1-methyl-*N*-[1-oxo-1-(3-phenylpropylamino)propan-2-yl]-9*H*-pyrido[3,4-b]indole-3-carboxamide | O=C(NC(C(=O)NCCCC=1C=CC=CC1)C)C=2N=C(C=3NC=4C=CC=CC4C3C2)C | 5.54 | COCONUT=CNP0177901, not found |  | methyl 2',15'-dimethyl-5,5'-dioxo-18'-oxaspiro[oxolane-2,14'-pentacyclo[8.8.0.0¹,¹⁷.0²,⁷.0¹¹,¹⁵]octadecan]-6'-ene-9'-carboxylate | CC12CCC(=O)C=C1CC(C3C24C(O4)CC5(C3CCC56CCC(=O)O6)C)C(=O)OC | 0.64 | C_24_H_30_O_6_ | COCONUT=CNP0164123, not found |  | 1-methyl-*N*-[1-oxo-1-(3-phenylpropylamino)propan-2-yl]-9*H*-pyrido[3,4-b]indole-3-carboxamide |

TABLE S3 cont.

| No | Parent mass | RT | Area | Adduct | Formula |  | FBMN | | | | |  | MSFINDER Annotation TOP 5 | | | |  | CFM-ID 3.0 | | | | |  | Final Annotation |
| --- | --- | --- | --- | --- | --- | --- | --- | --- | --- | --- | --- | --- | --- | --- | --- | --- | --- | --- | --- | --- | --- | --- | --- | --- |
|  |  |  |  |  |  |  | Name | Smiles | MQ Score | Source | Network |  | Name | Smiles | Score | Source |  | Name | Smiles | Score | Formula | Source |  |  |
|  |  |  |  |  |  |  |  |  |  |  |  |  | *N*-(6-{6-oxo-7,11-diazatricyclo[7.3.1.0²,⁷]trideca-2,4-dien-11-yl}pyridin-3-yl)-3-phenylpropanamide | O=C1C=CC=C2N1CC3CN(C4=NC=C(C=C4)NC(=O)CCC=5C=CC=CC5)CC2C3 | 5.46 | COCONUT=CNP0398612, not found |  |  |  |  |  |  |  |  |
|  |  |  |  |  |  |  |  |  |  |  |  |  | 2-methyl-4-({[4-(propan-2-yl)phenyl]methylidene}amino)-4,7,17-triazatetracyclo[8.7.0.0²,⁷.0¹¹,¹⁶]heptadeca-1(10),11,13,15-tetraene-3,6-dione | O=C1N2CCC=3C=4C=CC=CC4NC3C2(C(=O)N(N=CC5=CC=C(C=C5)C(C)C)C1)C | 5.39 | COCONUT=CNP0331937, not found |  |  |  |  |  |  |  |  |
|  |  |  |  |  |  |  |  |  |  |  |  |  | *N*-(4-{6-oxo-11-[(pyridin-2-yl)methyl]-7,11-diazatricyclo[7.3.1.0²,⁷]trideca-2,4-dien-3-yl}phenyl)acetamide | O=C1C=CC(C2=CC=C(C=C2)NC(=O)C)=C3N1CC4CN(CC5=NC=CC=C5)CC3C4 | 5.27 | COCONUT=CNP0039765, not found |  |  |  |  |  |  |  |  |
|  |  |  |  |  |  |  |  |  |  |  |  |  | 2-(4-benzoylpiperazin-1-yl)-*N*-{1*H*,2*H*,3*H*-cyclopenta[b]quinolin-9-yl}acetamide | O=C(C=1C=CC=CC1)N2CCN(CC(=O)NC=3C=4C=CC=CC4N=C5C3CCC5)CC2 | 5.09 | COCONUT=CNP0054113, not found |  |  |  |  |  |  |  |  |

TABLE S3 cont.

| No | Parent mass | RT | Area | Adduct | Formula |  | FBMN | | | | |  | MSFINDER Annotation TOP 5 | | | |  | CFM-ID 3.0 | | | | |  | | Final Annotation |
| --- | --- | --- | --- | --- | --- | --- | --- | --- | --- | --- | --- | --- | --- | --- | --- | --- | --- | --- | --- | --- | --- | --- | --- | --- | --- |
|  |  |  |  |  |  |  | Name | Smiles | MQ Score | Source | Network |  | Name | Smiles | Score | Source |  | Name | Smiles | Score | Formula | Source |  | |  |
| 29 | 415.2105 | 4.15 | 68520 | [M+H]^+^ | C_25_H_26_N_4_O_2_ |  | **—** | **—** | **—** | **—** | 17 |  | *N*-[6-(6-oxo-7,11-diazatricyclo[7.3.1.02,7]trideca-2,4-dien-11-yl)pyridin-3-yl]-3-phenylpropanamide | O=C1C=CC=C2N1CC3CN(C4=NC=C(C=C4)NC(=O)CCC=5C=CC=CC5)CC2C3 | 5.68 | COCONUT=CNP0398612, not found |  | Methyl 2',15'-dimethyl-5,5'-dioxo-18'-oxaspiro[oxolane-2,14'-pentacyclo[8.8.0.0¹,¹⁷.0²,⁷.0¹¹,¹⁵]octadecan]-6'-ene-9'-carboxylate | CC12CCC(=O)C=C1CC(C3C24C(O4)CC5(C3CCC56CCC(=O)O6)C)C(=O)OC | 0.64 | C_24_H_30_O_6_ | COCONUT=CNP0164123, not found |  | *N*-[6-(6-oxo-7,11-diazatricyclo[7.3.1.02,7]trideca-2,4-dien-11-yl)pyridin-3-yl]-3-phenylpropanamide | |
|  |  |  |  |  |  |  |  |  |  |  |  |  | 2-({1-methyl-9*H*-pyrido[3,4-b]indol-3-yl}formamido)-*N*-(3-phenylpropyl)propanamide | O=C(NC(C(=O)NCCCC=1C=CC=CC1)C)C=2N=C(C=3NC=4C=CC=CC4C3C2)C | 5.26 | COCONUT=CNP0177901, not found |  |  |  |  |  |  |  | |  |
|  |  |  |  |  |  |  |  |  |  |  |  |  | 2-methyl-4-[(4-propan-2-ylphenyl)methylideneamino]-4,7,17-triazatetracyclo[8.7.0.02,7.011,16]heptadeca-1(10),11,13,15-tetraene-3,6-dione | O=C1N2CCC=3C=4C=CC=CC4NC3C2(C(=O)N(N=CC5=CC=C(C=C5)C(C)C)C1)C | 5.21 | COCONUT=CNP0331937, not found |  |  |  |  |  |  |  | |  |
|  |  |  |  |  |  |  |  |  |  |  |  |  | *N*-[3-(morpholin-4-yl)propyl]-1-phenyl-9*H*-pyrido[3,4-b]indole-3-carboxamide | O=C(NCCCN1CCOCC1)C=2N=C(C=3C=CC=CC3)C=4NC=5C=CC=CC5C4C2 | 5.19 | COCONUT=CNP0348000, not found |  |  |  |  |  |  |  | |  |
|  |  |  |  |  |  |  |  |  |  |  |  |  | *N*-[4-[6-oxo-11-(pyridin-2-ylmethyl)-7,11-diazatricyclo[7.3.1.02,7]trideca-2,4-dien-3-yl]phenyl]acetamide | O=C1C=CC(C2=CC=C(C=C2)NC(=O)C)=C3N1CC4CN(CC5=NC=CC=C5)CC3C4 | 5.18 | COCONUT=CNP0039765, not found |  |  |  |  |  |  |  | |  |

TABLE S3 cont.

| No | Parent mass | RT | Area | Adduct | Formula |  | FBMN | | | | |  | MSFINDER Annotation TOP 5 | | | |  | CFM-ID 3.0 | | | | |  | Final Annotation |
| --- | --- | --- | --- | --- | --- | --- | --- | --- | --- | --- | --- | --- | --- | --- | --- | --- | --- | --- | --- | --- | --- | --- | --- | --- |
|  |  |  |  |  |  |  | Name | Smiles | MQ Score | Source | Network |  | Name | Smiles | Score | Source |  | Name | Smiles | Score | Formula | Source |  |  |
| 30 | 318.3034 | 3.83 | 160846 | [M+H]^+^ | C_18_H_39_NO_3_ |  | **—** | **—** | **—** | **—** | 18 |  | Phytosphingosine (*) | OCC(N)C(O)C(O)CCCCCCCCCCCCCC (*) | 7.41 | Plantae |  | Phytosphingosine | CCCCCCCCCCCCCCC(C(C(CO)N)O)O | 1 | C_18_H_39_NO_3_ | Metazoa |  | Phytosphingosine |
|  |  |  |  |  |  |  |  |  |  |  |  |  | 2-aminooctadecane-1,3,6-triol | OCC(N)C(O)CCC(O)CCCCCCCCCCCC | 6.52 | Plantae |  |  |  |  |  |  |  |  |
| 31 | 318.2997 | 3.78 | 338354 | [M+H]^+^ | C_18_H_39_NO_3_ |  | **—** | **—** | **—** | **—** | 18 |  | Phytosphingosine (*) | OCC(N)C(O)C(O)CCCCCCCCCCCCCC (*) | 7.29 | Plantae |  | Phytosphingosine | CCCCCCCCCCCCCCC(C(C(CO)N)O)O | 1 | C_18_H_39_NO_3_ | Metazoa |  | Phytosphingosine |
|  |  |  |  |  |  |  |  |  |  |  |  |  | 2-aminooctadecane-1,3,6-triol | OCC(N)C(O)CCC(O)CCCCCCCCCCCC | 6.39 | Plantae |  |  |  |  |  |  |  |  |
| 32 | 484.2565 | 5.29 | 10404 | [M+Na]^+^ | C_28_H_35_N_3_O_3_ |  | **—** | **—** | **—** | **—** | 21 |  | Paraherquamide F | O=C1NC2=C3C=CC(OC3=CC=C2C14CC56N(C(=O)C7(N(CCC7C)C5)CC6C4(C)C)C)(C)C | 5.85 | Fungi |  | **—** | **—** | **—** | **—** | **—** |  | Paraherquamide F |
|  |  |  |  |  |  |  |  |  |  |  |  |  | LSM-14658 | O=C1C(=CC=C2N1CC3C2N(C)C(C(=O)N4CCC(CC=5C=CC=CC5)CC4)C3CO)C=CC | 5.43 | ChEBI=CHEBI:103314, not found |  |  |  |  |  |  |  |  |
|  |  |  |  |  |  |  |  |  |  |  |  |  | Mangrovamide C | O=C1C2=C(OC(C)(C)C1)C=CC3=C2NC4=C3CC56N(C(=O)C7(N(CC(C)C7)C5)CC6C4(C)C)C | 5.41 | Fungi |  |  |  |  |  |  |  |  |

TABLE S3 cont.

| No | Parent mass | RT | Area | Adduct | Formula |  | FBMN | | | | |  | MSFINDER Annotation TOP 5 | | | |  | CFM-ID 3.0 | | | | |  | Final Annotation |
| --- | --- | --- | --- | --- | --- | --- | --- | --- | --- | --- | --- | --- | --- | --- | --- | --- | --- | --- | --- | --- | --- | --- | --- | --- |
|  |  |  |  |  |  |  | Name | Smiles | MQ Score | Source | Network |  | Name | Smiles | Score | Source |  | Name | Smiles | Score | Formula | Source |  |  |
|  |  |  |  |  |  |  |  |  |  |  |  |  | Methyl 2-[(2-hydroxyethyl)amino]-4-[4-(propan-2-yl)phenyl]-1*H*,2*H*,3*H*,4*H*,6*H*,7*H*,12*H*,12b*H*-indolo[2,3-a]quinolizine-6-carboxylate | O=C(OC)C1N2C(C3=CC=C(C=C3)C(C)C)CC(NCCO)CC2C=4NC=5C=CC=CC5C4C1 | 5.34 | COCONUT=CNP0265312, not found |  |  |  |  |  |  |  |  |
|  |  |  |  |  |  |  |  |  |  |  |  |  | 3-ethyl-2-({6-hydroxy-1*H*,2*H*,3*H*,4*H*,9*H*-pyrido[3,4-b]indol-1-yl}methyl)-10-methoxy-1*H*,2*H*,3*H*,4*H*,6*H*,7*H*,11b*H*-pyrido[2,1-a]isoquinolin-9-ol | OC=1C=CC=2NC3=C(C2C1)CCNC3CC4CC5C6=CC(OC)=C(O)C=C6CCN5CC4CC | 5.31 | Plantae |  |  |  |  |  |  |  |  |
| 33 | 505.1835 | 5.29 | 38375 | [M+K]^+^ | C_24_H_34_O_9_ |  | **—** | **—** | **—** | **—** | 21 |  | T-2 Toxin | O=C(OCC12CC(OC(=O)CC(C)C)C(=CC2OC3C(O)C(OC(=O)C)C1(C)C43OC4)C)C | 5.96 | Fungi |  | **—** | **—** | **—** | **—** | **—** |  | T-2 Toxin |
|  |  |  |  |  |  |  |  |  |  |  |  |  | 8-*N*-Pentanoylneosolaniol | O=C(OCC12CC(OC(=O)CCCC)C(=CC2OC3C(O)C(OC(=O)C)C1(C)C43OC4)C)C | 5.65 | Fungi |  |  |  |  |  |  |  |  |
|  |  |  |  |  |  |  |  |  |  |  |  |  | Cardivin C | O=C(OC1C2OC(=O)C(=C)C2C(OC(=O)C(C)C)C(=O)C(C)CC(O)CC1(O)C)C(=CC)C | 5.63 | Plantae |  |  |  |  |  |  |  |  |

TABLE S3 cont.

| No | Parent mass | RT | Area | Adduct | Formula |  | FBMN | | | | |  | MSFINDER Annotation TOP 5 | | | |  | CFM-ID 3.0 | | | | |  | Final Annotation |
| --- | --- | --- | --- | --- | --- | --- | --- | --- | --- | --- | --- | --- | --- | --- | --- | --- | --- | --- | --- | --- | --- | --- | --- | --- |
|  |  |  |  |  |  |  | Name | Smiles | MQ Score | Source | Network |  | Name | Smiles | Score | Source |  | Name | Smiles | Score | Formula | Source |  |  |
|  |  |  |  |  |  |  |  |  |  |  |  |  | Ajugamarin C1 | O=C1OCC(=C1)C(O)CC2(C)C(C)CC(OC(=O)C)C3(COC(=O)C)C2C(O)CCC43OC4 | 5.55 | Plantae |  |  |  |  |  |  |  |  |
|  |  |  |  |  |  |  |  |  |  |  |  |  | Ajugaciliatin G | O=C1OCC(=C1)C2OC3CCC(O)(CO)C4(COC(=O)C)C(OC(=O)C)CC(C)C(C)(C2)C34 | 5.55 | Archaeplastida |  |  |  |  |  |  |  |  |
| 34 | 489.2091 | 5.27 | 253293 | [M+Na]^+^ | C_24_H_34_O_9_ |  | **—** | **—** | **—** | **—** | 21 |  | T-2 Toxin (*) | O=C(OCC12CC(OC(=O)CC(C)C)C(=CC2OC3C(O)C(OC(=O)C)C1(C)C43OC4)C)C (*) | 6.33 | Fungi |  | **—** | **—** | **—** | **—** | **—** |  | T-2 Toxin |
|  |  |  |  |  |  |  |  |  |  |  |  |  | 8-*N*-Pentanoylneosolaniol | O=C(OCC12CC(OC(=O)CCCC)C(=CC2OC3C(O)C(OC(=O)C)C1(C)C43OC4)C)C | 6.01 | Fungi |  |  |  |  |  |  |  |  |
|  |  |  |  |  |  |  |  |  |  |  |  |  | Cardivin C | O=C(OC1C2OC(=O)C(=C)C2C(OC(=O)C(C)C)C(=O)C(C)CC(O)CC1(O)C)C(=CC)C | 5.98 | Plantae |  |  |  |  |  |  |  |  |
|  |  |  |  |  |  |  |  |  |  |  |  |  | Ajugamarin C1 | O=C1OCC(=C1)C(O)CC2(C)C(C)CC(OC(=O)C)C3(COC(=O)C)C2C(O)CCC43OC4 | 5.91 | Plantae |  |  |  |  |  |  |  |  |
|  |  |  |  |  |  |  |  |  |  |  |  |  | Ajugaciliatin G | O=C1OCC(=C1)C2OC3CCC(O)(CO)C4(COC(=O)C)C(OC(=O)C)CC(C)C(C)(C2)C34 | 5.91 | Archaeplastida |  |  |  |  |  |  |  |  |

TABLE S3 cont.

| No | Parent mass | RT | Area | Adduct | Formula |  | FBMN | | | | |  | MSFINDER Annotation TOP 5 | | | |  | CFM-ID 3.0 | | | | |  | Final Annotation |
| --- | --- | --- | --- | --- | --- | --- | --- | --- | --- | --- | --- | --- | --- | --- | --- | --- | --- | --- | --- | --- | --- | --- | --- | --- |
|  |  |  |  |  |  |  | Name | Smiles | MQ Score | Source | Network |  | Name | Smiles | Score | Source |  | Name | Smiles | Score | Formula | Source |  |  |
| 35 | 302.1324 | 4.29 | 15774 | [M+H]^+^ | C_19_H_15_N_3_O |  | **—** | **—** | **—** | **—** | 22 |  | 21-methyl-3,13,21-triazapentacyclo[11.8.0.0²,¹⁰.0⁴,⁹.0¹⁵,²⁰]henicosa-1,3,5,7,9,15,17,19-octaen-14-one (*) | O=C1C=2C=CC=CC2N(C3=C4N=C5C=CC=CC5=C4CCN13)C (*) | 4.30 | Plantae |  | Crinamine | COC1CC2C3(C=C1)C(CN2CC4=CC5=C(C=C34)OCO5)O | 0.65 | C_17_H_19_NO_4_ | Plantae |  | 21-methyl-3,13,21-triazapentacyclo[11.8.0.0²,¹⁰.0⁴,⁹.0¹⁵,²⁰]henicosa-1,3,5,7,9,15,17,19-octaen-14-one |
|  |  |  |  |  |  |  |  |  |  |  |  |  | 3-methyl-7-(4-methylphenyl)-4-phenyl-[1,2]oxazolo[3,4-d]pyridazine | N=1N=C(C=2C=CC=CC2)C=3C(=NOC3C)C1C4=CC=C(C=C4)C | 4.28 | COCONUT=CNP0057236, not found |  |  |  |  |  |  |  |  |
|  |  |  |  |  |  |  |  |  |  |  |  |  | 18-methyl-3,13,17-triazapentacyclo[11.8.0.0²,¹⁰.0⁴,⁹.0¹⁵,²⁰]henicosa-1(21),2(10),4,6,8,15(20),16,18-octaen-14-one | O=C1C2=CN=C(C=C2C=C3C=4NC=5C=CC=CC5C4CCN13)C | 4.24 | Archaeplastida |  |  |  |  |  |  |  |  |
|  |  |  |  |  |  |  |  |  |  |  |  |  | 3-phenyl-1,4-*bis*(pyridin-4-yl)azetidin-2-one | O=C1N(C=2C=CN=CC2)C(C=3C=CN=CC3)C1C=4C=CC=CC4 | 3.97 | COCONUT=CNP0266816, not found |  |  |  |  |  |  |  |  |
|  |  |  |  |  |  |  |  |  |  |  |  |  | 3,6-diamino-10-phenyl-9,10-dihydroacridin-9-one | O=C1C2=CC=C(N)C=C2N(C=3C=CC=CC3)C4=CC(N)=CC=C14 | 3.96 | COCONUT=CNP0047537, not found |  |  |  |  |  |  |  |  |
| 36 | 304.1312 | 4.29 | 4491 | [M+Na]^+^ | C_18_H_19_NO_2_ |  | **—** | **—** | **—** | **—** | 22 |  | Floribundine | OC=1C=C2C3=C(C1OC)C4=CC=CC=C4CC3N(C)CC2 | 5.65 | Plantae |  | 3-{[(4,5-dihydro-1*H*-imidazol-2-yl)methyl](4-methylphenyl)amino}phenol | CC1=CC=C(C=C1)N(CC1=NCCN1)C1=CC(O)=CC=C1 | 1.03 | C_17_H_19_N_3_O | COCONUT=CNP0342259, not found |  | Floribundine |

TABLE S3 cont.

| No | Parent mass | RT | Area | Adduct | Formula |  | FBMN | | | | |  | MSFINDER Annotation TOP 5 | | | |  | CFM-ID 3.0 | | | | |  | Final Annotation |
| --- | --- | --- | --- | --- | --- | --- | --- | --- | --- | --- | --- | --- | --- | --- | --- | --- | --- | --- | --- | --- | --- | --- | --- | --- |
|  |  |  |  |  |  |  | Name | Smiles | MQ Score | Source | Network |  | Name | Smiles | Score | Source |  | Name | Smiles | Score | Formula | Source |  |  |
|  |  |  |  |  |  |  |  |  |  |  |  |  | Lirinidine | OC1=C(OC)C=C2C3=C1C4=CC=CC=C4CC3N(C)CC2 | 5.54 | Plantae |  |  |  |  |  |  |  |  |
|  |  |  |  |  |  |  |  |  |  |  |  |  | Bharatamine | OC=1C=C2C(=CC1OC)CCN3CC=4C=CC=CC4CC23 | 5.54 | Plantae |  |  |  |  |  |  |  |  |
|  |  |  |  |  |  |  |  |  |  |  |  |  | 4-methoxy-10-methyl-10-azatetracyclo[7.7.1.0²,⁷.0¹³,¹⁷]heptadeca-1(16),2(7),3,5,13(17),14-hexaen-5-ol | OC=1C=C2C(=CC1OC)C=3C=CC=C4C3C(N(C)CC4)C2 | 5.38 | Archaeplastida |  |  |  |  |  |  |  |  |
|  |  |  |  |  |  |  |  |  |  |  |  |  | 1-(dibenzylamino) cyclopropane-1-carboxylic acid | O=C(O)C1(N(CC=2C=CC=CC2)CC=3C=CC=CC3)CC1 | 5.35 | COCONUT=CNP0264501, not found |  |  |  |  |  |  |  |  |
| 37 | 425.2151 | 5.92 | 178100 | [M+H]^+^ | C_22_H_32_O_8_ |  | **—** | **—** | **—** | **—** | 37 |  | Didrovaltrate | O=C(OC1CC2C(=COC(OC(=O)CC(C)C)C2C31OC3)COC(=O)CC(C)C)C | 5.76 | Plantae |  | 1-(3-cyano-3,3-diphenylpropyl)-4-phenylpiperidine-4-carboxylic acid | C1CN(CCC1(C2=CC=CC=C2)C(=O)O)CCC(C#N)(C3=CC=CC=C3)C4=CC=CC=C4 | 0.93 | C_28_H_28_N_2_O_2_ | COCONUT=CNP0347622, not found |  | Toxin Ht 2 |
|  |  |  |  |  |  |  |  |  |  |  |  |  | Toxin Ht 2 | O=C(OCC12CC(OC(=O)CC(C)C)C(=CC2OC3C(O)C(O)C1(C)C43OC4)C)C | 5.65 | Fungi |  |  |  |  |  |  |  |  |
|  |  |  |  |  |  |  |  |  |  |  |  |  | Scutalbin C | O=C(OC1CC(C)C(C)(C2OC3OC(O)CC3C2)C4CC5OC(O)C14C6(OC6)C5)C | 5.57 | Plantae |  |  |  |  |  |  |  |  |

TABLE S3 cont.

| No | Parent mass | RT | Area | Adduct | Formula |  | FBMN | | | | |  | MSFINDER Annotation TOP 5 | | | |  | CFM-ID 3.0 | | | | |  | Final Annotation |
| --- | --- | --- | --- | --- | --- | --- | --- | --- | --- | --- | --- | --- | --- | --- | --- | --- | --- | --- | --- | --- | --- | --- | --- | --- |
|  |  |  |  |  |  |  | Name | Smiles | MQ Score | Source | Network |  | Name | Smiles | Score | Source |  | Name | Smiles | Score | Formula | Source |  |  |
|  |  |  |  |  |  |  |  |  |  |  |  |  | 1-ethoxy-12-hydroxy-2-(hydroxymethyl)-11-methyl-7-methylidene-6-oxo-5,14-dioxatricyclo[9.2.1.0⁴,⁸]tetradecan-9-yl 2-methylbut-2-enoate | O=C(OC1CC2(OC(OCC)(CC2O)C(CO)CC3OC(=O)C(=C)C31)C)C(=CC)C | 5.55 | COCONUT=CNP0229628, not found |  |  |  |  |  |  |  |  |
|  |  |  |  |  |  |  |  |  |  |  |  |  | 10-(hydroxymethyl)-6,7-dimethoxy-6-methyl-3-methylidene-2,9-dioxo-dodecahydrocyclodeca[b]furan-4-yl 2-methylbut-2-enoate | O=C(OC1CC(OC)(C)C(OC)CC(=O)C(CO)CC2OC(=O)C(=C)C21)C(=CC)C | 5.54 | Archaeplastida |  |  |  |  |  |  |  |  |
| 38 | 927.6614 | 0.81 | 113660 | [M+H]^+^ | C_59_H_90_O_8_ |  | **—** | **—** | **—** | **—** | 39 |  | **—** | **—** | **—** | **—** |  | Thermozeaxanthin-13 | CC1=C(C(CC(C1)O)(C)C)C=CC(=CC=CC(=CC=CC=C(C)C=CC=C(C)C=CC2=C(CC(CC2(C)C)OC3C(C(C(C(O3)COC(=O)CCCCCCCCCC(C)C)O)O)O)C)C)C | 0.9 | C_59_H_90_O_8_ | Bacterium |  | Thermozeaxanthin-13 |
