# Supplementary figures and images for: The screening for marine fungal strains with high potential in alkaloids production by in situ colony assay and LC-MS/MS based secondary metabolic profiling
Source: Front Microbiol. 2023 May 3;14:1144328. doi: 10.3389/fmicb.2023.1144328 (PMC10191116; doi:10.3389/fmicb.2023.1144328)

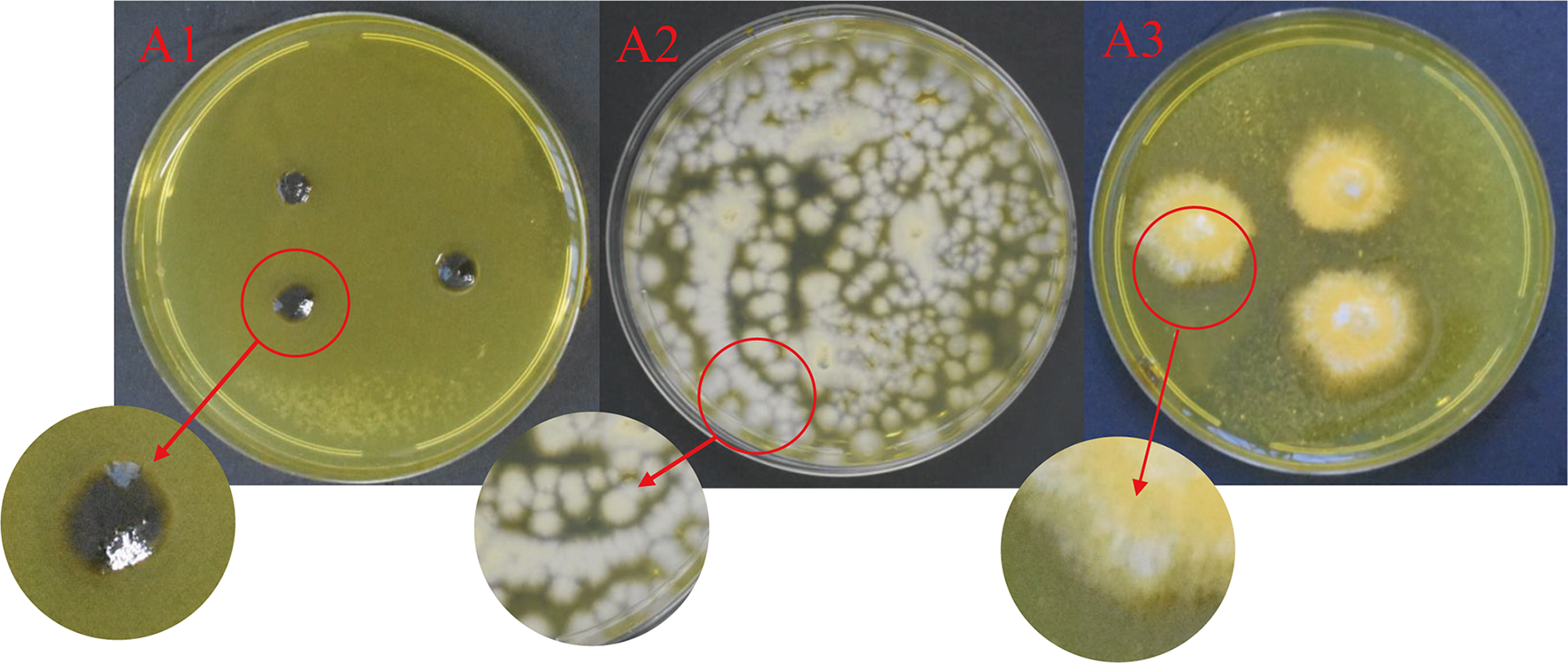

Supplement: Supplementary file 2 [file Image_1.TIF]

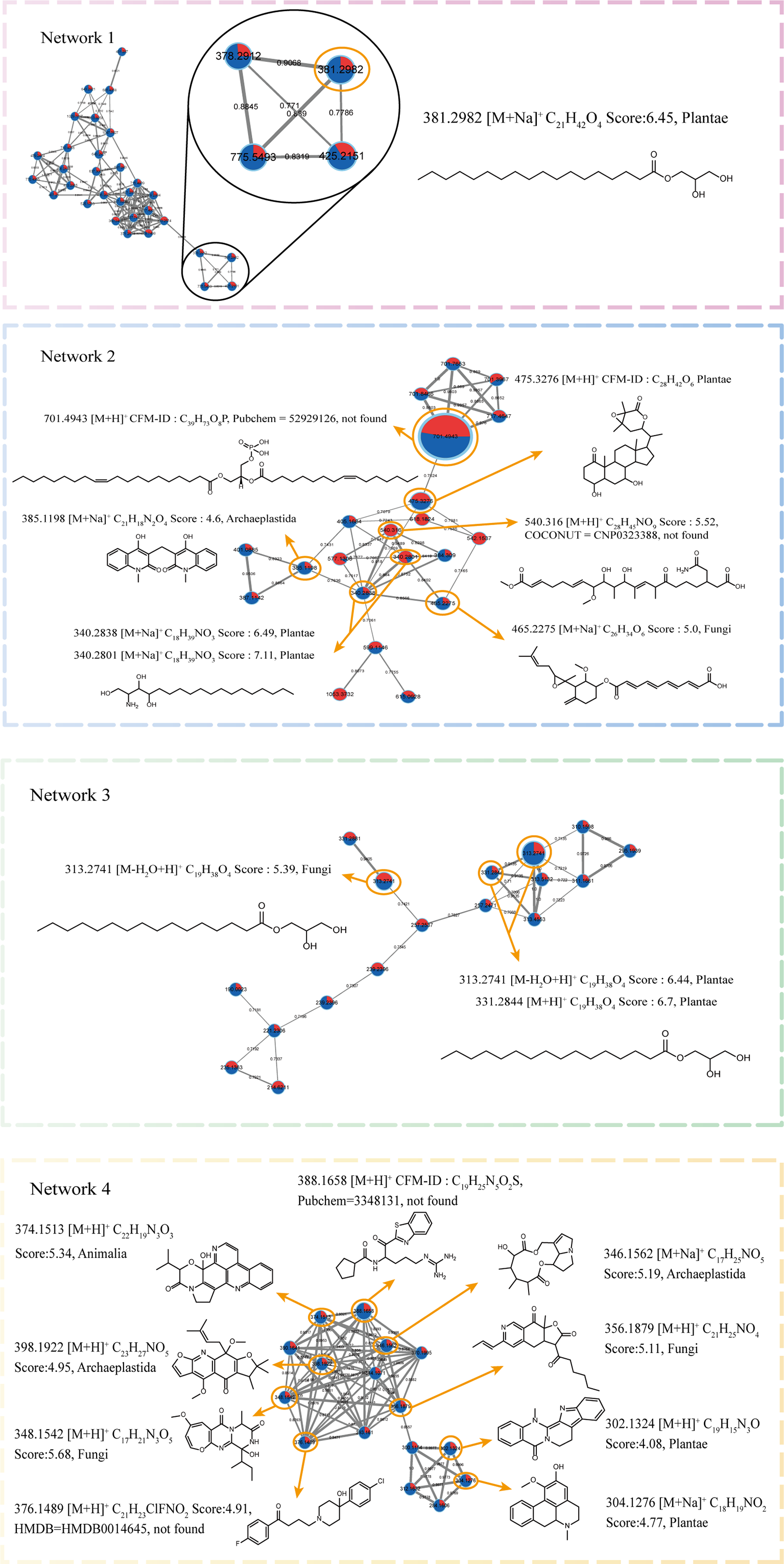

Supplement: Supplementary file 3 [file Image_2.TIF]

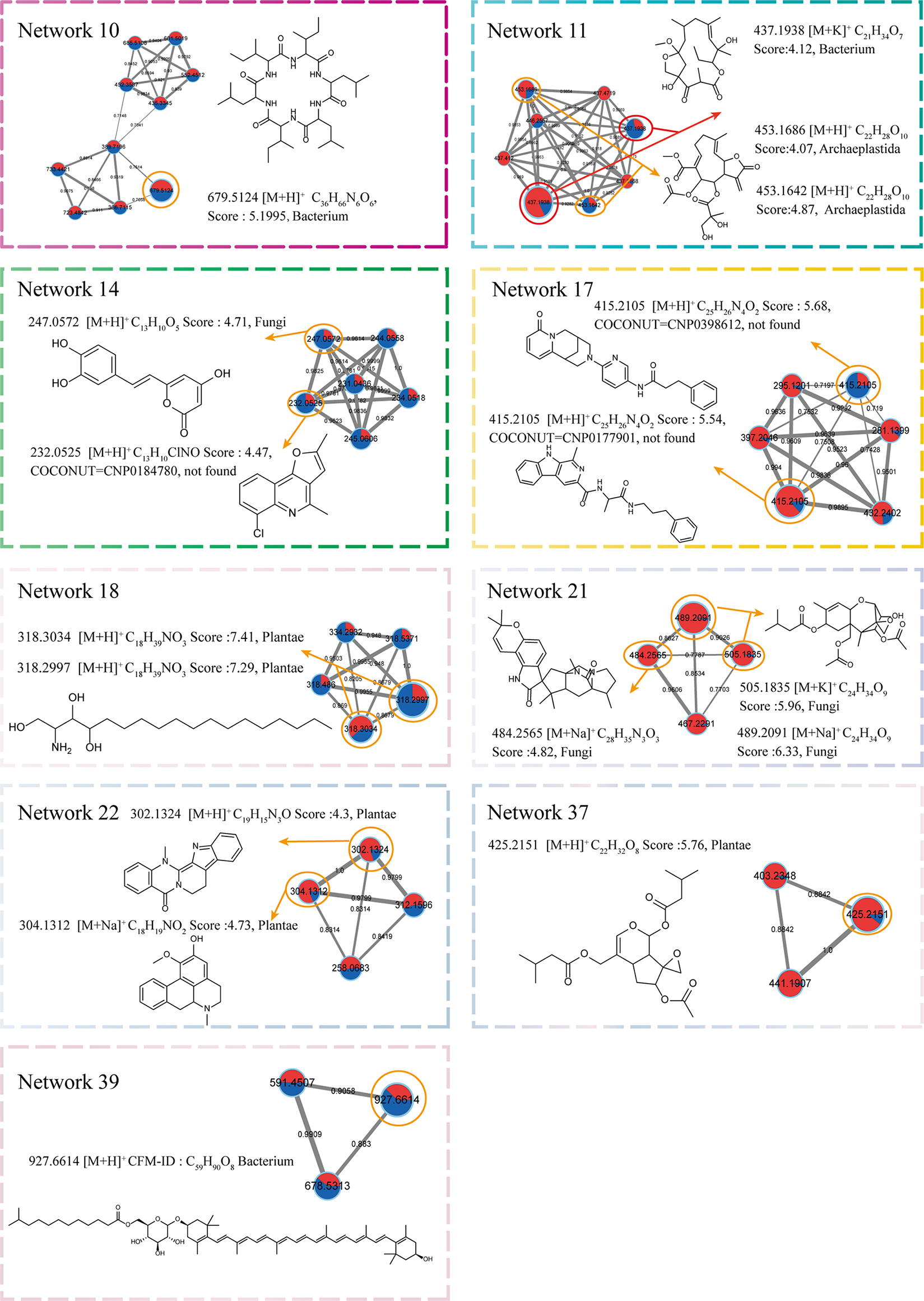

Supplement: Supplementary file 4 [file Image_3.TIF]
